# A developmental shift in venom toxin expression underpins a major prey switch in Australian brown snakes

**DOI:** 10.1186/s12915-026-02699-0

**Published:** 2026-07-25

**Authors:** Jory van Thiel, Cara F. Smith, Taline D. Kazandjian, Rohit N. Patel, India C. Cullen, Stephanie French, Rachael Da Silva, Laura-Oana Albulescu, Nathan Dunstan, Jeroen Kool, Michael K. Richardson, Stephen P. Mackessy, Nicholas R. Casewell, R. Manjunatha Kini, Anthony J. Saviola, Timothy N. W. Jackson, Cassandra M. Modahl

**Affiliations:** 1 Centre for Snakebite Research & Interventions, Liverpool School of Tropical Medicine, Liverpool, L3 5QA United Kingdom; 2 Institute of Biology Leiden, Leiden University, Leiden, 2333 BE The Netherlands; 3 Department of Biochemistry and Molecular Genetics, School of Medicine, University of Colorado, Aurora, 80045 United States of America; 4 Venom Supplies Pty. Ltd., Tanunda, SA 5352 Australia; 5 Division of Bioanalytical Chemistry, Department of Chemistry and Pharmaceutical Sciences, Faculty of Sciences, Amsterdam Institute of Molecular and Life Sciences (AIMMS), Vrije Universiteit Amsterdam, Amsterdam, 1081 HV The Netherlands; 6 Department of Biological Sciences, University of Northern Colorado, Greeley, CO 80639 United States of America; 7 Department of Biological Sciences, Faculty of Science, National University of Singapore, Singapore, 117558 Singapore; 8 Department of Pharmacology, Yong Loo Lin School of Medicine, National University of Singapore, Singapore, 117600 Singapore; 9 Department of Cellular, Molecular and Genetic Medicine, Virginia Commonwealth University, Richmond, 23298 United States of America; 10 Australian Venom Research Unit, Department of Pharmacology and Therapeutics, University of Melbourne, Melbourne, VIC 3010 Australia; 11 Present address: Howard Hughes Medical Institute and Department of Biology, University of Maryland, College Park, 20742 United States of America

**Keywords:** Gene regulation, Genotype–phenotype mapping, Life history traits, Extended phenotype, Phenotypic plasticity, Snake venom

## Abstract

**Background:**

Many animals change traits over the course of their life history. It has been proposed that some of these developmental changes evolve in concert with shifts in ecology, but there are relatively few clear examples, and identifying the underlying molecular changes is challenging. Australian brown snakes (*Pseudonaja* spp.), one of Australia's most venomous snakes, may provide such an example, as previous work has shown that these species undergo a dietary shift from reptiles to mammals, accompanied by differences in venom activity.

**Results:**

We show that the venom composition of adult brown snakes is distinguished by the collective expression of four procoagulant toxin families, which are absent in juvenile life stages, where venom composition is instead dominated by neurotoxins. The developmental onset of expression across all four procoagulant toxins correlates with a functional shift in venom activity to one that disrupts the blood-clotting system of mammalian prey. We further show that this developmental timing in both toxin expression and procoagulant venom activity coincides with a broader ecological transition in which Australian brown snakes begin to prey on mammals. In contrast, we found that the smallest brown snake species, a lizard specialist throughout its lifetime, only expresses neurotoxins and shows no comparable developmental shift in either procoagulant toxin expression or procoagulant activity.

**Conclusion:**

Our findings suggest that a coordinated shift in the expression of four procoagulant toxin gene families transitions the venom from a neurotoxic phenotype in lizard-feeding juveniles to a potent procoagulant phenotype in mammal-feeding adults. These results provide a clear example of how the concerted upregulation of multiple genes can drive a functional shift in a key ecological trait, linking changes in toxin gene expression to venom activity and, ultimately, to prey-capture strategy.

**Supplementary Information:**

The online version contains supplementary material available at https://doi.org/10.1186/s12915-026-02699-0.

## Background

Developmental changes in morphology, physiology, and behavior are common across the animal kingdom. When animals undergo developmental changes in their ecology, the use of different traits may increase their probability of survival, and ultimately successful reproduction. For example, herbivorous tadpoles use a distinct set of bitter taste receptors from insectivorous adults, enabling each life stage to detect different dietary poisons [1]. Similarly, in butterflies (and other winged insects) a major morphological transition occurs during the pupal stage, as wings develop and enable adults for long-distance movement and migration [2]. These developmental transitions are underlain by distinct patterns of gene activity, but linking changes in gene expression, phenotypes, and ecological conditions is challenging, especially across different life stages.

Venomous snakes offer opportunities to study these relationships. Venom proteins are ecologically important for venomous snakes as they facilitate prey capture, and their measurable genotype–phenotype relationship have allowed investigations to uncover co-evolutionary dynamics between predators and prey [3–8]. Snakes are also gape-limited predators, therefore a developmental increase in gape size allows for the ingestion of larger prey, resulting in a greater potential diversity of different prey types over their lifespan. Such dietary expansion may drive developmental shifts in venom composition and therefore, it is not surprising that many studies have reported evidence of ontogenetic changes in snake venom (for example, see [9–17]). Numerous studies have suggested links between venom ontogeny and prey, but there are not many clear examples of how developmental changes in gene expression transform the phenotype of the venom and how these phenotypic shifts relate to changes in prey.

One interesting case may involve Australian brown snakes (*Pseudonaja* spp.), which exhibit a change in ecological niche during development that coincides with transitions in morphology [18], behavior [19, 20], and venom activity [21, 22]. When most species of brown snake reach approximately 60 cm snout-vent length (SVL), they make an ecological shift from nocturnal foraging on small lizards, to diurnal foraging and a generalist diet that includes mammals [19, 20]. This shift in prey is facilitated by the accelerated growth of skull structures involved in feeding, enabling the ingestion and handling of larger prey (e.g. native mammals) [20]. Australian brown snake venom largely comprises a mixture of potent neurotoxins and hemotoxins, with the latter being responsible for one of the fastest blood-clotting effects among Australian venomous snakes [23]. Previous work has shown that the procoagulant activity of brown snake venom differs between hatchlings and adults, as plasma coagulation [21] and enzymatic assays [22] indicate its absence in hatchlings but presence in adults. This observation suggests that the developmental timing of the venom shift might coincide with the prey change from reptiles to mammals.

In this study, we have used a combination of diet, gene, protein, and functional analyses to determine whether the ontogenetic dietary shift in Australian brown snakes is correlated with developmental changes in venom composition and activity. We focused on two coagulopathic species that are known to prey upon mammals as adults, the Eastern brown snake (*P*. *textilis*) and strap-snouted brown snake (*P*. *aspidorhyncha*) [20, 22], and compare these species to the ringed brown snake (*P*. *modesta*), a lizard-specialist that has been shown to lack procoagulant venom activity [21, 24]. We found that four procoagulant toxin families are not expressed in *P*. *textilis* and *P*. *aspidorhyncha* juvenile venom glands but are highly expressed in adults. We next quantified changes in venom proteins across different developmental stages, demonstrating that their lethal procoagulant toxins are largely absent in lizard-eating life stages but become highly abundant in mammal-eating adults. Finally, we found the developmental timing of procoagulant toxin expression to correlate with a functional shift in venom activity, disrupting mammalian blood coagulation by activating a key component in the clotting cascade. The coincidence of an ontogenetic prey shift with a major venom change in Australian brown snakes illustrates elegantly how species can adapt to shifting ecological niches by altering a key trait over the course of their lifetime.

## Results

### Procoagulant toxins define venom characteristics of adult brown snakes

To profile differences in venom composition between hatchling and adult Australian brown snakes, we first identified toxin genes expressed in the venom glands using de novo transcriptomics. To validate all annotated toxin transcripts, we compared venom gland expression levels to that of liver tissue from the same individuals, confirming that toxin transcripts were significantly overexpressed in the venom gland (Additional file 1: Fig. S1). Additionally, we paired these custom de novo assembled venom gland transcriptomes with shotgun venom proteomics: trypsin digestion followed by high-performance liquid chromatography (HPLC) coupled with high-resolution tandem mass spectrometry (LC–MS/MS) analysis. This integrated approach identified a total of 18 venom gene families in *P*. *textilis*, *P*. *aspidorhyncha*, and *P*. *modesta*, with most toxin families being common to all three species (Additional file 1: Table. S1).

**Fig. 1:**
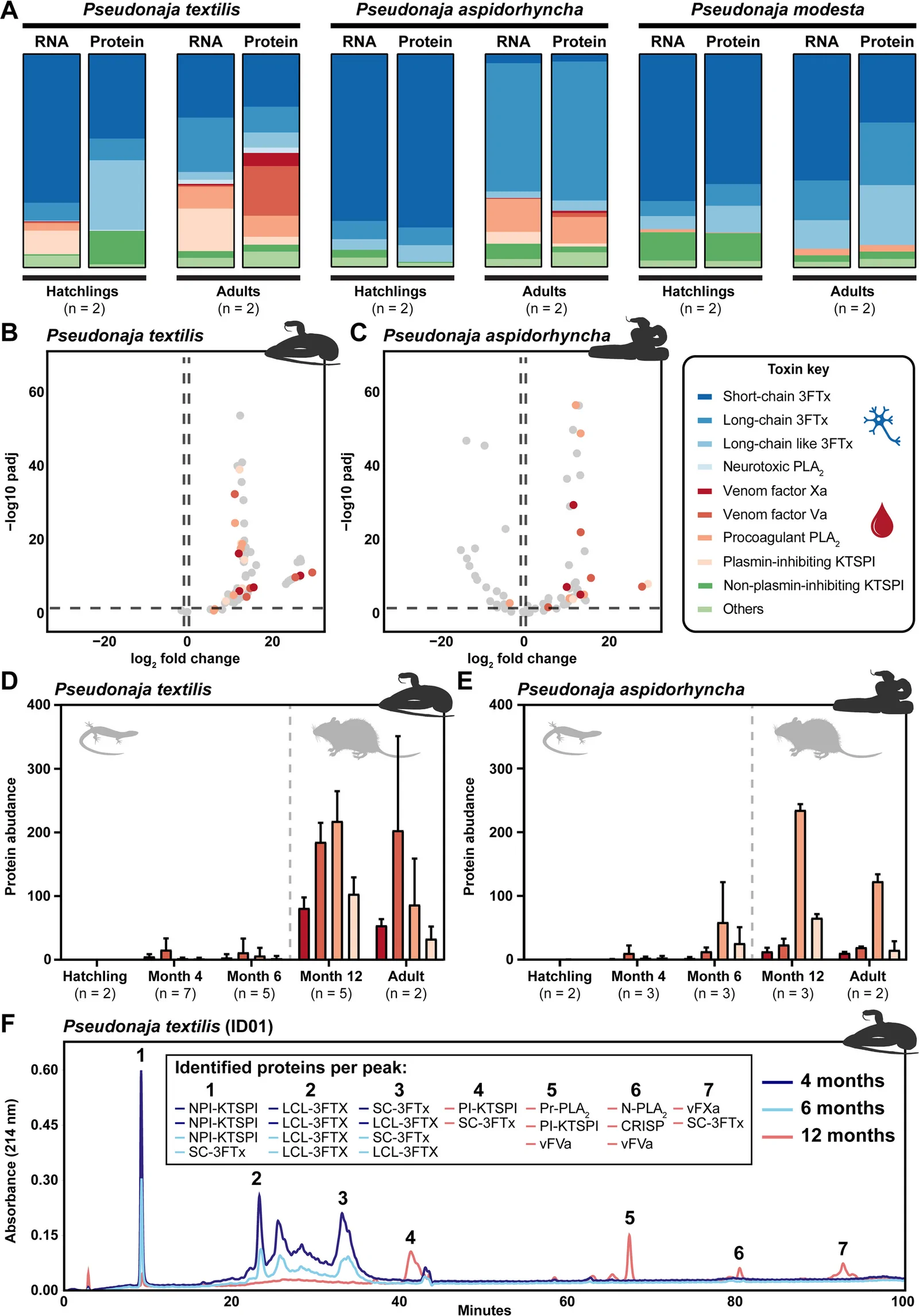
The expression of procoagulant toxins coincides with a dietary shift to mammalian prey. **A** Comparative transcriptomic and proteomic profiling between hatchlings and adult snakes. Both transcript per million (TPM) and intensity values were normalized by the total sum and displayed as percentages. TPM values of waprin and cobra venom factor were excluded from hatchling transcriptomes to enhance visualization given their absence in the corresponding proteome (*n* = 2). **B** and **C** Volcano plots reveal that almost all procoagulant toxin transcripts (displayed in shades of red) are upregulated in adults. The Log₂ fold-change cutoff is ± 1.0, with an adjusted *p*-value cutoff of 0.05. Only the major toxin families were included in these plots. **D** and **E** Total procoagulant toxin abundance levels show that four toxin classes are upregulated following the dietary shift. Each bar represents the average ± standard deviation for each life stage. The dashed grey lines indicate the developmental timing of the prey shift. **F** Reverse-phase chromatographic separation of *P*. *textilis* venom, followed by tandem mass spectrometry (LC–MS/MS), revealed that all four procoagulant toxin families are present after the dietary change. The peak numbers correspond to the two most abundant proteins detected in each peak. Only venom factor Va was also included due to its biological significance in brown snake venom. Furthermore, factor Xa and factor Va were not detected in any other life stages. Abbreviations: 3FTx, three-finger toxin; CRISP, cysteine-rich secretory protein; KTSPI, Kunitz-type serine protease inhibitor; LCL-3FTx, long-chain-like three-finger toxin; N-PLA_2_, neurotoxic phospholipase A_2_; NPI-KTSPI, non-plasmin-inhibiting Kunitz-type serine protease inhibitor; PI-KTSPI, plasmin-inhibiting Kunitz-type serine protease inhibitor; PLA_2_, phospholipase A_2_; Pr-PLA_2_, procoagulant phospholipase A_2_; SC-3FTx, short-chain three-finger toxin; vFVa, venom factor Va; vFXa, venom factor Xa

Venom gland transcriptomes and venom proteomes were composed of several toxins that disrupt coagulation in prey. In both the transcriptomic and proteomic datasets, we identified four toxin families known to contribute to the procoagulant phenotype, venom factor Xa (vFXa), venom factor Va (vFVa), group I phospholipase A_2_ (PLA_2_), and Kunitz-type serine protease inhibitors (KTSPI; Fig. 1A). These procoagulant toxin families have previously been isolated and functionally characterized in *P*. *textilis* venom [25–32]. vFXa and vFVa together form the prothrombinase complex, a potent converter of prothrombin to thrombin in mammalian blood [25–27, 32, 33]. Interestingly, vFVa and vFXa were not detected in the venom of *P*. *textilis* and *P*. *aspidorhyncha* hatchlings (Fig. 1A). Furthermore, we did not detect vFXa or vFVa transcripts or proteins in either hatchling or adult *P*. *modesta* (Fig. 1A). Procoagulant PLA_2_ isoforms [28] and KTSPI isoforms with inhibitory effects against the clot-degrading serine protease plasmin [29–31] were found exclusively in the venom of adult *P*. *textilis* and *P*. *aspidorhyncha*, but not in hatchlings (Fig. 1A). We concluded that our de novo assembled toxin transcripts, along with their matching venom proteins, are *bona fide* procoagulant toxins based on their near-identical sequences to previously confirmed brown snake procoagulant toxins (Additional file 1: Fig. S2–5) [25–32]. This conclusion was further supported by monophyletic clustering in Bayesian [34] and maximum likelihood [35] inferred phylogenetic analyses: vFXa (Bayesian posterior probability (BPP): 1.00; maximum likelihood ultrafast bootstrap approximation (UFBoot): 99; Additional file 1: Fig. S6–7), vFVa (BPP: 1.00; UFBoot: 100; Additional file 1: Fig. S8–9), procoagulant PLA_2_ (BPP: 1.00; UFBoot: 100; Additional file 1: Fig. S10–11), and plasmin-inhibiting KTSPI (BPP: 1.00; UFBoot: 94; Additional file 1: Fig. S12–13).

Aside from these procoagulant toxins, the venom gland transcriptomes and venom proteomes of all three brown snake species — in both hatchling and adult life stages — were dominated by three-finger toxins (3FTxs; Fig. 1A). These 3FTxs could be categorized into three subgroups based on structural characteristics: short-chain 3FTxs, long-chain 3FTxs, and long-chain-like 3FTxs, all of which are known brown snake neurotoxins that target acetylcholine receptors and cause neuromuscular paralysis (Additional file 1: Fig. S14) [36–38]. The 100% sequence identity of functionally confirmed neurotoxins, detected in both hatchling and adult venoms, demonstrates that both life stages possess neurotoxic activity (Additional file 1: Fig. S14). In one *P*. *textilis* adult (*n* = 2), we also detected four PLA_2_ proteins and transcripts (subunits A, B, C and D) that together form a presynaptic neurotoxin [39, 40]. Finally, the remaining 13 toxin gene families comprised only a minor part of the total number of transcripts (2.49–4.13%) and proteins (1.15–6.91%; Fig. 1A) across samples. Given the low abundance of these non-procoagulant toxin families, it seems unlikely that they play a significant role in the phenotypic venom shift observed.

### Timing of procoagulant toxin expression coincides with dietary change

The distinct procoagulant toxins identified in brown snake venoms raises the question of which toxin or combination of toxins underlies the prey shift from lizards in juveniles to mammals in adults. To test this, we first performed differential gene expression analysis between hatchling and adult venom glands [41]. Among the upregulated venom transcripts in adults, we show that all four procoagulant toxin families (i.e. vFXa, vFVa, procoagulant PLA_2_, and plasmin-inhibiting KTSPI) are significantly upregulated in the two procoagulant brown snake species (*P. textilis* and *P. aspidorhyncha*; adjusted *p*-value [Padj] < 0.05; Fig. 1B–C). In *P. textilis* adults, 18 (out of 22) procoagulant toxin transcripts were significantly upregulated (9.13–29.97 log2FoldChange; Additional file 1: Fig. S15 and Additional file 1: Table. S2) and 12 (out of 13) in *P*. *aspidorhyncha* adults (10.41–29.79 log2FoldChange; Additional file 1: Fig. S15 and Additional file 1: Table. S3). Interestingly, only one PLA_2_ transcript was downregulated in *P*. *aspidorhyncha*, and none were downregulated in *P*. *textilis* (Additional file 1: Fig. S15 and Additional file 1: Table. S2–3). In contrast to the two larger species, the only procoagulant toxins significantly upregulated in *P*. *modesta* adults were two procoagulant PLA_2_ transcripts (10.18–10.83 log2FoldChange; Additional file 1: Fig. S15 and Additional file 1: Table. S4).

Subsequently, we examined whether the timing of the procoagulant toxin shift coincides with dietary change using time-series proteome profiling. We focused only on venom proteins, as these are the primary trait used for prey capture. To achieve this, we collected venom samples of multiple brown snakes, including siblings, over developmental time (i.e. hatchlings, 4 months, 6 months, 12 months and adults). For each procoagulant species, our individuals had an average body size, measured as snout-vent length (SVL), of 37.2 ± 1.6 cm (*P*. *textilis*; *n* = 5) and 37.4 ± 2.8 cm (*P*. *aspidorhyncha*; *n* = 3) at six months old, and 71.2 cm ± 2.9 cm (*P*. *textilis*; *n* = 5) and 58.3 ± 2.7 cm (*P*. *aspidorhyncha*; *n* = 3) at 12 months old (Additional file 1: Table. S5). Previous dietary analysis has shown that the dietary shift occurs at around 60 cm SVL [19, 20], so we use months 6 to 12 in our species as a proxy for when brown snakes begin preying on mammals.

In both species with procoagulant adult venoms, our time-series proteome profiling revealed that all four procoagulant toxin families are absent or show low abundance at stages when these species prey on lizards but are highly abundant at stages when they prey on mammals (Fig. 1D–E). In *P*. *textilis*, we found a statistically significant association between life stages that prey on mammals and levels of procoagulant toxins: vFXa (*p* < 0.01), vFVa (*p* < 0.01), procoagulant PLA_2_s (*p* < 0.01), and plasmin-inhibiting KTSPIs (*p* < 0.01). In *P*. *aspidorhyncha*¸ we found statistical support for vFXa (*p* < 0.01) and procoagulant PLA_2_ (*p* = 0.01), but vFVa (*p* = 0.09) and plasmin-inhibiting KTSPI (*p* = 0.07; Mann–Whitney U test) were close to significant. Collectively, these findings provide evidence that the timing of dietary shift in procoagulant Australian brown snakes coincides with the upregulation of procoagulant toxins.

To detect developmental shifts in toxin isoforms within individual snakes, we performed LC–MS/MS to identify unique protein peaks before and after dietary change. In the venom of one representative *P*. *textilis* individual (ID01), we observed several unique peaks at 12 months that were absent at 4 or 6 months (Fig. 1F). MS/MS identification revealed that all unique peaks in month 12 are dominated by the four procoagulant toxins: vFXa, vFVa, procoagulant PLA_2_s, and plasmin-inhibiting KTSPIs (Fig. 1F). In contrast, the unique peaks in the venom from younger stages consisted of different neurotoxic 3FTx isoforms and non-plasmin-inhibiting KTSPIs (Fig. 1F). A similar trend was seen in the venom of one representative *P*. *aspidorhyncha* individual (ID02), but only procoagulant PLA_2_s and plasmin-inhibiting KTSPIs were identified at month 12 (Additional file 1: Fig. S16). Hatchling venom chromatogram profiles for all three species showed an absence of peaks corresponding to larger toxins (e.g. vFX or vFV) (Additional file 1: Fig. S17). Overall, this provides a second line of evidence supporting the association between these four procoagulant toxin families and the venom phenotypic shift.

To exclude the possibility that endogenous toxin inhibitors play a role in the regulation of the procoagulant venom shift, we identified venom gland expression levels of: i) tissue factor pathway inhibitor and antithrombin, both regulators of endogenous factor X activity [42, 43], ii) activated protein C, an endogenous regulator of factor V activity [44], and iii) PLA_2_ inhibitors, regulators of PLA_2_ activity [45]. Our analyses show no obvious patterns that suggest co-expression of endogenous inhibitors may govern phenotypic changes in venom activity (Additional file 1: Fig. S18–21). Furthermore, these toxin inhibitors were not detected in any of the hatchling venoms proteomes. Together, these analyses support the hypothesis that the observed procoagulant venom shift is driven by changes in toxin expression, rather than being regulated by endogenous toxin inhibitors.

### Timing of procoagulant venom activity coincides with dietary change

**Fig. 2:**
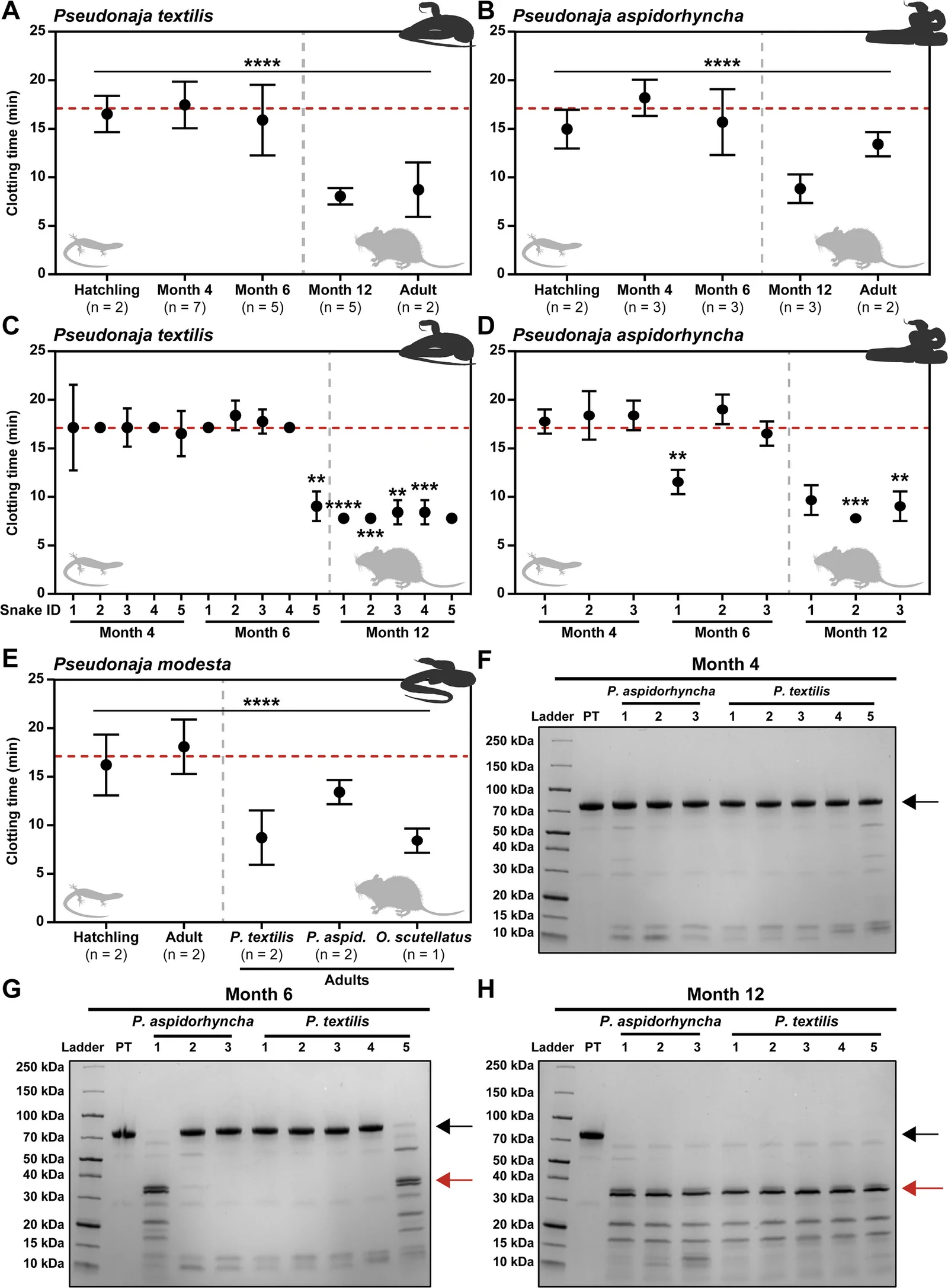
Functional venom shift coincides with a dietary shift to mammalian prey. **A** and **B** coagulation profiles for each life stage of the procoagulant brown snake species as measured by a plasma coagulation assay. Each data point represents the mean ± standard deviation of multiple measurements from the different individuals per life stage. The data shown corresponds to the maximum concentration used in the assay, namely 20 ng. The red dashed line represents the negative control (no venom added) and normal clotting in the presence of recalcification (*n* = 76). Grey dashed lines represents the change in diet from small lizards to mammals. The Mann–Whitney U test was used to assess statistical differences in clotting time between lizard-eating and mammalian-eating life stages. **C** and **D** coagulation profiles for individuals procoagulant brown snakes reveal a change in venom activity between months 6 and 12. Note that one individual in both species begins to display procoagulant venom activity at month 6. The Kruskal–Wallis test with multiple comparisons was used to assess statistical differences between life stages. **E** Comparative coagulation profiles of *P*. *modesta* hatchling and adult venoms show lack of procoagulant venom activity. **F**, **G**, and **H** Prothrombin cleavage profiles reveal a shift in thrombin release following the dietary shift. Mammalian prothrombin was preincubated with venom, and cleavage was visualized using SDS-PAGE gel electrophoresis. The black arrows indicate prothrombin (~ 72 kDa), and the red arrow indicates liberated thrombin (~ 36 kDa). Note that one individual in both species begins to activate prothrombin at month 6. Prothrombin alone was used as a negative control. Asterisks (*) denote significance levels: ** *P* ≤ 0.01, *** *P* ≤ 0.001, and **** *P* ≤ 0.0001. Abbreviations: kDa, kilodalton; Min, Minutes; PT, prothrombin

To confirm that the upregulation of procoagulant toxins underlies a phenotypic shift in venom activity, we measured the coagulopathic activity of the venoms from all developmental stages. Using a standardized plasma coagulation assay — an in vitro model for measuring how venom alters clotting — we generated dose–response curves for all venom samples (2.5 ng, 5 ng, 10 ng, and 20 ng; *n* = 5 independent repeats, with 2 replicates per repeat) (Additional file 1: Fig. S22–24) [46]. By comparing the effects of maximum venom doses (i.e., 20 ng) across developmental stages, we revealed that *P. textilis* and *P. aspidorhyncha* venoms shift from non-coagulant to procoagulant activity at month 12, the life stage at which they begin to prey on mammals (Fig. 2A–B). Interestingly, more detailed analysis of the individual siblings found that in both species, the largest individuals began to show evidence of mild coagulopathic venom activity at 6 months (i.e. *P*. *textilis* ID05 is 39.6 cm SVL and *P*. *aspidorhyncha* ID01 is 41.0 cm SVL; Fig. 2C–D). Our findings demonstrate that the coagulopathic shift in venom activity coincides with the timing of dietary change toward mammalian prey, and that this shift in venom activity may be initiated slightly before the dietary transition.

We further show that the venom of both hatchlings and adults of the lizard-specialist *P*. *modesta* lacked procoagulant venom activity (Fig. 2E). Interestingly, ancestral state estimations of adult body size in the monophyletic brown snake–taipan clade reveal a remarkable reduction in *P*. *modesta* (Additional file 1: Fig. S25). While all brown snake and taipan species grow to over 100 cm, *P*. *modesta* has undergone a substantial reduction in body size, with adults measuring only 35.9 cm SVL (males) and 41.2 cm SVL (females) on average [19], from inferred ancestral body sizes of 86.9 cm in males (95% CI: 67.8–106.0) and 85.4 cm in females (95% CI: 66.3–104.5) (Additional file 1: Fig. S25). This suggests that the evolutionary loss of procoagulant venom activity in adults [22, 24], supported by the absence of two major procoagulant toxins (vFXa and vFVa; Fig. 1A), is closely associated with small adult body size and a diet of lizards. This further corroborates the link between procoagulant venom and mammalian prey in Australian brown snakes.

Given that prothrombin is a key clotting factor in the coagulation cascade and is the molecular target of the major procoagulant toxin (vFXa:vFVa) in Australian brown snake venoms [25, 26, 32], we next performed a prothrombin activation assay. Australian brown snake venom (0.25 μg) from different age groups (4 months, 6 months, 12 months) was incubated with human prothrombin (2 μg) as a representative mammalian ortholog and prothrombin cleavage visualized by SDS-PAGE (Fig. 2F–H). Our analysis revealed that all *P. textilis* and *P. aspidorhyncha* venoms at month 4 lack cleavage activity, illustrated by the absence of liberated thrombin molecules at 36 kDa (Fig. 2F). Subsequently, most month six venoms lack activity too, except for one *P. textilis* (ID5) and one *P. aspidorhyncha* (ID1) that show a thrombin band (Fig. 2G). Notably, these are the same individuals that show mild procoagulant activity at month six in the plasma coagulation assay (Fig. 2C–D). Ultimately, all *P. textilis* and *P. aspidorhyncha* specimens transitioned to a prothrombin-cleaving venom by 12 months (Fig. 2H).

In summary, our results show: i) delayed expression of four procoagulant toxin families in the venom gland; ii) the presence of procoagulant toxins in the adult snake venoms; and iii) that procoagulant activities — at both the phenotypic and molecular target levels — are directly linked with a body-size-correlated prey shift towards capturing mammalian prey.

## Discussion

**Fig. 3:**
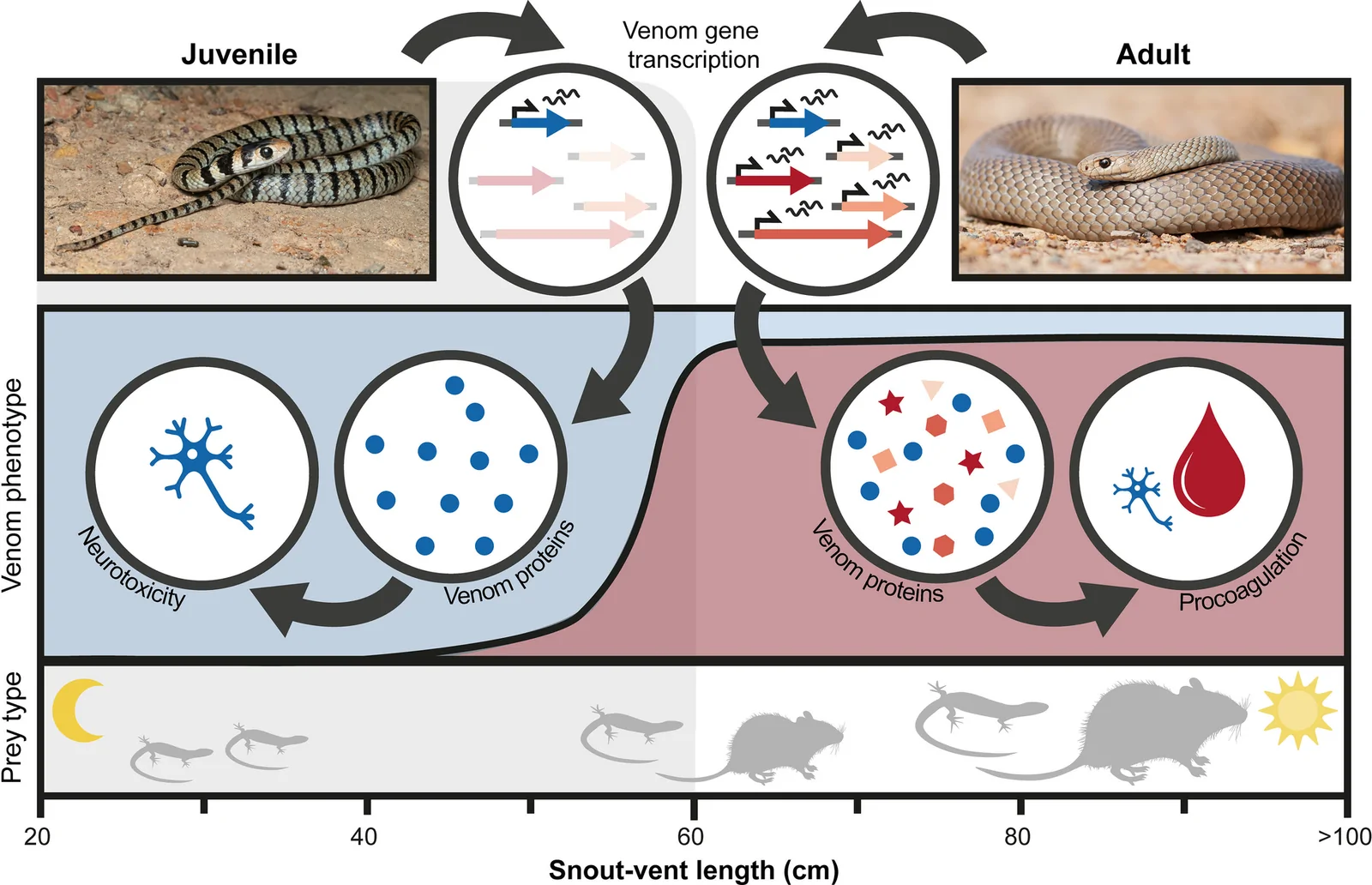
A developmental model of venom adaptations in Australian brown snakes to capture distinct types of prey. Juvenile venom glands are characterized by the expression of neurotoxins, whereas adult venom glands additionally express multiple procoagulant toxins. While juvenile brown snakes either lack or exhibit low levels of procoagulant toxins and corresponding blood-clotting activity, brown snakes over 60 cm in snout-vent length express high levels of procoagulant toxins, leading to rapid blood-clotting and circulatory collapse. The coordinated timing of the venom phenotypic shift corresponds with juvenile brown snakes foraging nocturnally on small lizards until they exceed 60 cm in snout-vent length, at which point they adopt a diurnal foraging strategy and begin incorporating mammals as prey. Photo credit: Ákos Lumnitzer

We have demonstrated that Australian brown snakes display a coordinated shift in venom composition and activity that coincides with a dietary shift from small reptiles to mammalian prey during development. When brown snakes are smaller than 60 cm SVL, they are nocturnal foragers on small lizards [19, 20]. During these early developmental stages, expression levels of the four procoagulant venom gene families are extremely low (Fig. 1), and these venoms consequently lack procoagulant activity (Fig. 2). Instead, juvenile snakes have a venom dominated by neurotoxins (Fig. 1 and Additional file 1: Fig. S14). When brown snakes surpass 60 cm in body size, they shift to a diurnal-foraging strategy which includes mammalian prey [19, 20]. The timing of this dietary shift is accompanied by the upregulation of four distinct procoagulant toxin families (Fig. 1), which underpins a clear shift in venom activity to a potent blood-clotting venom in adults (Fig. 2). The developmental relationships between venom gene expression, venom proteins, venom activity, and primary prey type are schematized in Fig. 3.

Many studies of ontogenetic venom shifts examine changes in snake venom composition (via proteomics and/or transcriptomics) and correlate these changes with differences in whole-venom activity, which are typically assessed through in vitro functional experiments and/or in vivo lethality assays [11, 12, 14, 16, 47, 48]. The fact that many venom toxins are encoded by large multigene families, coupled with limited functional characterization of many toxin variants, makes it difficult to identify the specific genes responsible for developmental shifts in venom phenotype and their relationship to prey use. Integrative systems that directly connect gene expression, toxin function, and ecological dynamics remain limited, particularly in Elapidae snakes. In this context, Australian brown snakes provide an excellent model system, as their venom and its individual toxins have been well characterized, revealing multiple procoagulant toxins [25–32] as well as several distinct neurotoxins [36–40]. The well-resolved biochemical activity of these individual toxins enables direct linkage between developmental changes in the expression of different procoagulant toxin genes (genotype), their relative abundance in venom (ecological trait), the corresponding procoagulant activity (phenotype), and their potential association with shifts in prey (ecological interaction). Strikingly, all four procoagulant toxin classes (vFXa, vFVa, procoagulant PLA₂s, and plasmin-inhibiting KTSPI) begin to be expressed when Australian brown snakes start predating on mammals. The key procoagulant toxin underlying this transition to mammalian prey is presumably the prothrombinase complex (vFXa:vFVa), which has been shown to cause near-instant cardiovascular collapse in mammals (i.e. rats and mice), followed by rapid death [49–51]. In contrast, significantly higher doses of procoagulant PLA_2_s [28] or plasmin-inhibiting KTSPI [52] appear to be non-lethal in mice. This suggests that while these toxins may help subdue prey through synergy with the other procoagulant component, the prothrombinase complex (vFXa:vFVa) seems to be the primary mammal-subjugation toxin.

The incredible ability of the venom prothrombinase complex to cause immediate cardiovascular collapse in rodents explains the high toxicity of *P*. *textilis* venom to lab mice (LD_50_ assay via subcutaneous injection) [53]. Another study reported similar toxicity levels but extended their lethality analysis to various frogs and lizards [54]. The authors found that injecting *P*. *textilis* venom at over 100 times the lethal dose for mice had no effect on Australian skink species (administered via intramuscular injection) [54]. This is surprising given that skinks are the dominant prey type of juvenile brown snakes [19, 20], and therefore raises the question of how early life stages effectively capture their lizard prey. We suggest that the absence of procoagulant venom activity in juvenile snakes results in a venom dominated by potent neurotoxins, which may facilitate more effective immobilization of small lizard prey (Fig. 1A) [36–38]. This is plausible, as neurotoxic 3FTxs are the predominant constituent of the venom proteomes in all lizard-eating life stages of species analyzed (i.e. *P*. *textilis* hatchling: 82.6% neurotoxic 3FTx; *P*. *aspidorhyncha* hatchling: 97.8%; *P*. *modesta* hatchling: 83.8%; and *P*. *modesta* adult: 89.6%; Fig. 1A).

Taipans (*Oxyuranus* spp.), the closest relatives of Australian brown snakes, also produce venoms with multiple procoagulant toxins (e.g. venom prothrombinase complex) [55] and neurotoxins (e.g. presynaptic PLA_2_s and postsynaptic 3FTx) [56, 57]. Toxicity assays with coastal taipan (*Oxyuranus scutellatus*) venom showed that the procoagulant effects are responsible for acute toxicity in mice before neurotoxic symptoms, such as paralysis, can manifest [58]. This indicates that while neurotoxins are consistently expressed across all brown snake life stages, the expression of procoagulant toxins in adult snakes enhances the venom’s potency toward mammalian prey. Interestingly, taipans are specialist predators that feed exclusively on mammals, and they retain procoagulant venom expression (including the venom prothrombinase complex) throughout all life stages [21, 59, 60]. This suggests that the developmental venom shift in Australian brown snakes is a lineage-specific adaptation to prey changes as they transition from juveniles to adults. The phenomenon of developmental venom adaptations to prey, as proposed in snakes [9, 10, 12, 16, 21, 22], also extends to other venomous lineages, as supported by evidence from Australian box jellyfish [61], cone snails [62], and sea anemones [63].

One future direction of this study is to investigate what genetic changes enable coordinated shifts in the expression of multiple, unrelated genes encoding the diverse procoagulant toxins in Australian brown snakes. A key implication of our findings is that similar functional outcomes, namely, prey-associated shifts in venom phenotype, have evolved across distantly related snake lineages, raising the question of how convergent ecological pressures shape venom systems built on distinct regulatory architectures. In viperids, ontogenetic venom shifts linked to dietary transitions have been associated with changes in transcriptional regulation and epigenetic state [17], suggesting that developmental timing of toxin gene expression can be modulated at multiple levels. These regulatory and epigenetic processes have been proposed to drive ontogenetic venom changes in the Eastern diamondback rattlesnake (*Crotalus adamanteus*) [17]. Another candidate mechanism involves small non-coding RNA molecules (microRNAs), which bind to mRNA transcripts and may prevent their translation into venom toxins. This post-transcriptional regulatory mechanism has been suggested to underlie developmental shifts in venom composition in the Central American rattlesnake (*Crotalus simus*) [13]. A similar mechanism may also underlie ontogenetic venom shifts in *P*. *textilis*, as previous work has identified several microRNAs predicted to target transcripts encoding vFXa, vFVa, and procoagulant PLA₂ toxins [64]. These distinct genetic mechanisms could be empirically validated using snake venom gland organoids [65], which provide a platform for the functional dissection of regulatory pathways controlling toxin gene expression.

## Conclusions

While developmental changes after birth are ubiquitous throughout the animal kingdom, their direct link to ecological changes and the underlying molecular changes is not always well understood. Our study provides a clear example of this relationship, demonstrating how developmental shifts in gene expression mediate a remarkable phenotypic change in the venom of one of Australia’s most venomous snakes in association with the capture of different prey. The coordinated expression of four procoagulant toxin families transforms a neurotoxic venom in juveniles, used for capturing small lizards, into a powerful procoagulant venom in adults, used for subduing large mammals. Therefore, our findings suggest an evolutionary adaptation to capture different types of prey across an animal’s lifespan.

## Methods

### Venom and tissues

The snake venoms, as well as venom gland and liver tissues, were provided by Venom Supplies Pty Ltd (Tanunda, SA, Australia). Full details of samples are displayed in Additional file 1: Table. S5. The venom samples from months 4, 6, and 12 of *P*. *textilis* and *P*. *aspidorhyncha* were collected from siblings to minimize intraspecific venom variation. All snakes used in this study were exclusively kept on a rodent diet.

### Tissue collection, RNA extraction and RNA-sequencing

Venom glands and livers were collected 4 days following manual venom extraction from two adult and two hatchling *Pseudonaja textilis, P. aspidorhyncha* and *P*. *modesta* that had been humanely euthanized. The hatchling and adult specimens of *P*. *textilis* and *P*. *aspidorhyncha* originated from different localities, but this does not affect their procoagulant venom phenotype [24, 49, 66]. All tissues were stored in RNAlater reagent (Thermo Fisher Scientific Inc., Waltham, MA, USA) after dissection. Tissues were homogenized, and total RNA isolated with TRIzol reagent (Thermo Fisher Scientific Inc., Waltham, MA, USA), following the manufacturer’s protocol. RNA was quantified by Nanodrop (Thermo Fisher Scientific Inc., Waltham, MA, USA) and Qubit fluorometer (Thermo Fisher Scientific Inc., Waltham, MA, USA), and quality evaluated on an Agilent 2100 Bioanalyzer (Agilent Technologies, Palo Alto, CA, USA) to confirm a minimum RIN of seven. One µg of total RNA from livers, adult venom glands, and hatchling venom glands (two hatchling individuals from each species were pooled to increase RNA amounts) was used as input for poly(A) enrichment using a NEBNext Poly(A) mRNA Magnetic Isolation Module (New England BioLabs Inc., Ipswich, MA, USA). mRNA was then fragmented to sizes of approximately 200–400 base pairs, first and second strand cDNA synthesis carried out, cDNA purified with Axygen® AxyPrep MAG PCR clean-up (Corning Life Sciences, Cambridge, UK) and end repair, dA-tailing, and T-A ligation of adaptors performed with a NEBNext Ultra RNA Library Prep Kit for Illumina sequencing kit (New England BioLabs Inc., Ipswich, MA, USA). Libraires were amplified by PCR for 11 cycles using P5 and P7 primers for Illumina sequencing, followed by Axygen® AxyPrep MAG PCR clean-up and final RNA-seq libraries validated on an Agilent 2100 Bioanalyzer and quantified by Qubit. RNA-seq libraries were then pooled in equal concentrations and sequenced on a HiSeq 2500 instrument to obtain at least 10 million 150 bp paired-end reads per library. Library preparation, sequencing, demultiplexing, and initial quality control was performed by GENEWIZ (Suzhou, Jiangsu, China).

### *De novo* transcriptome assembly, annotation, and differential transcript expression

Read quality was evaluated with the Java program FastQC, and low-quality reads (Phred + 33 score < 30) and contaminating adaptor sequences removed using Trimmomatic [67] v0.39 with a sliding window of 4 bps. Venom gland transcriptomes were first assembled de novo with Trinity v2.4.0 [68]. A second de novo assembled transcriptome was completed using Extender (k-mer size 100 and 10,000 starting seeds) [69]. Reads were first merged with PEAR [70] (Paired-End read mergeR v0.9.6; default parameters) and used as input for the Extender assembly. Trinity and Extender assemblies were concatenated for each individual snake (except hatchlings for each species where the RNA from two individuals had been pooled), and redundancy and contigs less than 150 base pairs removed with CD-HIT v4.6.8 [71, 72]. These contig sets were used as input into ToxCodAn [73] to identify toxin coding sequences (CDS) for each individual snake or hatchling group, and a final nonredundant (processed with CD-HIT) toxin and putative toxin CDS set was obtained for each species. RSEM v1.3.0 [74] with Bowtie2 [75] v2.2.6 was used to align reads to this CDS set to determine transcript abundance. These CDS sets were then manually curated to identify toxins with an abundance of at least 10 transcripts per million (TPM), a full length CDS, and the presence of a signal peptide [76]. CD-HIT v4.6.8 [71, 72] was used to cluster amino acid sequences with 100% identity to determine toxin conservation between individuals and species. Venom gland and liver reads were then mapped with RSEM v1.3.0 [74] and Bowtie2 to the curated toxin contig set for each species and the species’ curated contig set plus annotated P*. textilis* genome (assembly EBS10Xv2-PRI) for non-toxin abundance determinations. Abundance values (TPM) were then used as input into DESeq2 [41] to identify differentially expressed transcripts in the venom glands of hatchling and adult snakes.

### Shotgun proteomics

Lyophilized venom samples were reconstituted at an approximate concentration of 4.0 mg/mL in Millipore-filtered water, vortexed, and centrifuged for 5 min at 9,500 × g. Protein concentration was then determined in triplicate with a Nanodrop using the Absorbance 280 protocol, and 260/280 ratio was recorded. Reconstituted stock samples were frozen at −20 °C until use and then thawed and centrifuged at 9,500 × g for 5 min to pellet cellular debris. Approximately 30 μg of venom were resuspended in 8 M urea/0.1 M Tris (pH 8.5) and reduced with 5 mM TCEP (tris (2-carboxyethyl) phosphine) for 20 min at room temperature. Samples were alkylated with 50 mM 2-chloroacetamide for 15 min in the dark at room temperature and then diluted fourfold with 100 mM Tris–HCl (pH 8.5) and trypsin digested at an enzyme/substrate mass ratio of 1:20 overnight at 37 °C. To stop the reaction, samples were acidified with formic acid (FA), and digested peptides were purified with Pierce™ C18 Spin Tips (Thermo Fisher Scientific Inc., Waltham, MA, USA) according to the manufacturer's protocol. Samples were dried in a speed vacuum and resuspended in 0.1% FA. Liquid chromatography tandem mass spectrometry (LC–MS/MS) was performed using an Easy-nLC 1000 instrument coupled with a Q Exactive™ HF Mass Spectrometer (both from Thermo Fisher Scientific Inc., Waltham, MA, USA). Peptides were loaded on a C18 column (100 μM inner diameter × 20 cm) packed in-house with 2.7 μm Cortecs C18 resin, and separated at a flow rate of 0.4 μl/minute with solution A (0.1% FA) and solution B (0.1% FA in ACN) under the following conditions: isocratic at 4% B for 3 min, followed by 4%−32% B for 102 min, 32%−55% B for 5 min, 55%−95% B for 1 min and isocratic at 95% B for 9 min. Data-dependent acquisition (DDA) was performed, and the top 15 most abundant precursors were considered for MS/MS analysis. Fragmentation spectra were searched against an in-house *Pseudonaja* database containing translated sequences derived from our venom gland transcriptomes of all three species and the *P*. *textilis* global transcriptome (assembly EBS10Xv2-PRI) using the MSFragger-based FragPipe computational platform [77, 78]. The global transcriptome was included to ensure that no venom proteins were missing. Reverse decoys and contaminants were included in the search database. Cysteine carbamidomethylation was selected as a fixed modification, oxidation of methionine was selected as a variable modification, and precursor-ion mass tolerance and fragment-ion mass tolerance were set at 20 ppm and 0.4 Da, respectively. Up to 2 missed tryptic cleavages were allowed and the protein-level false discovery rate (FDR) was set to < 1%. The relative abundance of major toxin families was compared across samples using total spectral intensity [79]. 

### Reversed-phase high performance liquid chromatography

Approximately 100 ug of venom was loaded on a Waters HPLC system (Waters Corporation, Milford, MA, USA). The separation was done using a Jupiter C4 column (4.6 × 150 mm; Phenomenex, Inc., Torrance, CA, USA) with a flow rate of 1 mL/minute and the following gradient of acetonitrile in 0.1% trifluoroacetic acid; 0–15%/5 min; 15–40%/100 min; 50–80%/5 min; 80–95%/5 min. Elution was monitored at 214 nm with a Waters 2487 Dual λ Absorbance Detector (Waters Corporation, Milford, MA, USA). Fractions corresponding to each peak were collected and frozen at − 80 °C before lyophilization. Fractions were then loaded into wells of a NuPAGE Novex Bis–Tris 12% acrylamide Mini Gel (Life Technologies, Inc., Grand Island, NY, USA) and electrophoresed in MES (2-(N-morpholino)ethanesulfonic acid) buffer under reducing conditions for 45 min at 175 V. Electrophoretic protein bands subjected to liquid chromatography-tandem mass spectrometry (LC–MS/MS) for identification were first excised from Coomassie-stained gels, destained, and subjected to in-gel reduction, alkylation, and overnight trypsin digestion as previously described [80]. Following the overnight digestion, samples were acidified with 5% FA and tryptic peptides were extracted in 30 μl of 50% acetonitrile/1% FA. Digests were dried in a vacuum centrifuge and redissolved in 0.1% FA for mass spectrometry as described above.

For separations of hatchling venoms, a GE Health Sciences ÅKTApurifier P-900 FPLC system was used with proteins/peptides were monitored at 280 nm. Approximately 100 µg of venom in 100 µL PBS was injected onto a Phenomenex 4.6 × 150 mm Jupiter C_4_ column (300 Å pore, 5 µm). Solvent A was 0.1% TFA in 18.2 MΩ Millipore-filtered distilled water, and B was 0.1% TFA in acetonitrile; flow rate was 1.0 mL/minute. The gradient consisted of 5% B for 5 min, 5–15% over 3 min, and then a linear gradient over 100 min (15%−40% B) was used to elute samples. A rapid gradient (5 min) of 40–100% B was followed by a wash of the column with 100% B for 5 min and then flow returned to 5% B over 5 min.

### Phylogenetic analyses

Amino acid sequences for factor X, factor V, group I PLA_2_, KTSPI were retrieved from the UniProt and NCBI database and supplemented with our sequenced transcripts. Homologous sequences of the eastern fence lizard (*Sceloporus undulatus*) were used as outgroups. Incomplete sequences and sequences with assembling or several sequence errors were excluded. The signal peptides were manually removed based on the SignalP algorithm [76]. In some cases, the removal of signal peptides resulted in sequences becoming 100% identical, with one of the two being removed to enhance tree quality. Subsequently, amino acid sequences were aligned using the Multiple Sequence Comparison by Log-Expectation (MUSCLE) algorithm [81] in AliView [82]. The resulting alignments for each toxin family were used to estimate phylogenetic relationships using Bayesian and maximum likelihood inferred analysis. First, the amino acid substitution models were selected according to the Akaike Information Criterion (AIC) using 6 discrete gamma categories in ModelGenerator v.0.85 [83]. The best fitting model for each toxin family was: vFXa, Jones–Taylor–Thornton (JTT) + G; vFVa, JTT + G + F; group I PLA_2_, Whelan and Goldman (WAG) + G; KTSPI, WAG + G. Bayesian phylogenetics was performed using MrBayes v.3.2.7 [34]. The Markov chain Monte Carlo algorithm was set to four simultaneous runs with four chains (three hot, one cold) for 15,000,000 generations with sampling frequency of 100. The temperature parameter for heating the chains was set to 0.2. The average standard deviation of split frequencies was set with a stop value of 0.01. A majority rule consensus tree was calculated from the set of remaining trees after discarding 25% as burn-in. Maximum likelihood phylogenetics was performed using IQ-TREE v.2.2.2.6 [35]. The branch support was tested by ultrafast bootstrap approximation (UFBoot) using 10.000 iterations [84]. Then, the output trees were edited using iTol [85].

### Ancestral state reconstruction

The ancestral state of adult body sizes was estimated using a previously constructed phylogeny [86–89]. The *Vermicella*-*Neelaps* clade was used as the sister clade to root the ancestral state estimation [90]. Snout–vent length (SVL) data for adult males and females from each species and locality (where available) were collected from the literature (overview is shown in Additional file 1: Table. S6) [19, 91–95]. Ancestral states were estimated via maximum likelihood using the contMap function in the “phytools” package under default settings [96]. ACE values were extracted using the “ace” function of the APE package [97]. The analysis was performed separately on male and female SVL data to account for sexual dimorphism. All analysis were performed in R studio version 2022.12.0.353.

### Procoagulant assays

To assess which brown snake venoms exhibited procoagulant activity, we performed a 384-well plate plasma-based absorbance assay. First, a reaction solution was created using a 2:2:1 ratio of CaCl_2_ (Sigma-Aldrich Corp., St. Louis, MO, USA), phospholipid solution (“Reagent 1” from the C. K. Prest 5 kit, Diagnostica Stago Inc., Asnières-sur-Seine, Île-de-France, France) and Owren Koller buffer (OK; Diagnostica Stago Inc., Asnières-sur-Seine, Île-de-France, France). Phospholipids and calcium were added because procoagulant toxins, including factor X and factor V, use these cofactors for activity (for example, see [26, 98, 99]). Citrated bovine plasma (VWR International, Radnor, PA, USA) was defrosted in a water bath at 37 °C and then centrifuged at 2000 RPM for 5 min to remove any precipitant and kept on ice until further use. To each well, 10 µL of venom (20 ng, 10 ng, 5 ng, or 2.5 ng) was added, followed by 25 µL of the reaction solution, then 15µL of plasma added using a Multidrop™ Combi reagent dispenser (Thermo Fisher Scientific Inc., Waltham, MA, USA) and immediately read by the CLARIOstar® Plus plate reader (BMG Labtechusing, Ortenberg, Hesse, Germany) under the following settings: i) temperature: 37 °C, ii) kinetic assay, iii) 595 nm wavelength, iv) 20 flashes per well, v) horizontal reading direction (left to right), and vi) cycle time 187 s (minimum required) for 12 cycles. The negative control consisted of OK buffer only, while the positive control was a representative procoagulant venom from the coastal taipan (*Oxyuranus scutellatus*) [24, 60, 98]. Seven plates were run, with each sample measured in duplicate per plate. Two repeats were excluded using a Pearson’s r correlation matrix with 0.7 as a cutoff value, performed in Graphpad Prism 10.3.1. The clotting period (i.e., the time between clot initiation and full clotting in the negative controls) was determined to be between cycle 3 and 8 (6.2 and 21.8 min respectively; *n* = 5) using the first derivative of absorbance against cycle time in GraphPad Prism 10.3.1. The Area Under the Curve (AUC) for each measurement was calculated for the clotting period using GraphPad Prism 10.3.1, which were compared to evaluate spontaneous coagulation to venom-induced coagulation activity. This dataset was not normally distributed, and thus we used non-parametric statistical tests. The Mann–Whitney U test was used to assess statistical differences between lizard-eating and mammalian-eating life stages. The Kruskal–Wallis test with multiple comparisons was used to assess statistical differences between life stages.

### Prothrombin activation assay

Venoms from 4-, 6- and 12-month-old *P*. *textilis* and *P*. *aspidorhyncha* were examined for their ability to degrade prothrombin using reducing SDS-PAGE. Venom samples (0.25 µg) and prothrombin (2 µg) (Haematological Technologies, Inc., Essex Junction, VT, USA) were made up to a volume of 10 µL with PBS and incubated at 37 °C for 60 min in a water bath. The reaction was stopped by the addition of 2 µL 6X loading dye (New England BioLabs Inc., Ipswich, MA, USA) plus 15% β-mercaptoethanol, then samples were heated for 5 min at 100 °C using a heating block. Electrophoresis was performed with pre-cast, 4–20%, stain-free polyacrylamide gels (Bio-Rad Laboratories Inc., Hercules, CA, USA) using a Tris–glycine buffer. Next, the gels were stained with Coomassie Brilliant Blue R250 (Sigma-Aldrich Corp., St. Louis, MO, USA), followed by destaining in distilled water for 1 h before imaging. These gels were left in distilled water for 5 days and imaged daily.

## Supplementary Information

Supplementary Material 1. Fig. S1 to S25. Fig. S1. Comparative expression levels of identified venom toxin transcripts in liver and venom gland tissues. Fig. S2. Venom factor X sequence alignment. Fig. S3. Venom factor V sequence alignment. Fig. S4. Group I phospholipase A_2_ sequence alignment. Fig. S5. Kunitz type serine protease inhibitor sequence alignment. Fig. S6. Bayesian inference-derived amino acid phylogeny of factor X sequences. Fig. S7. Maximum-likelihood estimation-derived amino acid phylogeny of factor X sequences. Fig. S8. Bayesian inference-derived amino acid phylogeny of factor V sequences. Fig. S9. Maximum-likelihood estimation-derived amino acid phylogeny of factor V sequences. Fig. S10. Bayesian inference-derived phylogeny of group I phospholipase A_2_ sequences from elapid venoms. Fig. S11. Maximum-likelihood estimation-derived phylogeny of group I phospholipase A_2_ sequences from elapid venoms. Fig. S12. Bayesian inference-derived phylogeny of Kunitz-type serine protease inhibitor sequences from elapid venoms. Fig. S13. Maximum-likelihood estimation-derived phylogeny of Kunitz-type serine protease inhibitor sequences from elapid venoms. Fig. S14. Sequence alignment of three-finger toxins with identical, functionally confirmed neurotoxins. Fig. S15. Log2FoldChange of procoagulant toxin transcripts in adult venom gland tissue of Australian brown snakes. Fig. S16. Chromatographic venom profiles of Strap-snouted brown snakeindividual number 02. Fig. S17. Comparative chromatographic venom profiles of hatchling *Pseudonaja* species. Fig. S18. Comparative expression levels of tissue factor pathway inhibitors in venom glands and livers. Fig. S19. Comparative expression levels of antithrombin in venom glands and livers. Fig. S20. Comparative expression levels of activated protein C in venom glands and livers. Fig. S21. Comparative expression levels of phospholipase A_2_ inhibitors in venom glands and livers. Fig. S22. Coagulation profiles for all Eastern brown snakevenoms included in this study. Fig. S23. Coagulation profiles for all strap-snouted brown snakevenoms included in this study. Fig. S24. Coagulation profiles for all ringed brown snakevenoms included in this study. Fig. S25. Ancestral state estimation of adult body size in the monophyletic *Oxyuranus*-*Pseudonaja* clade reveals a significant reduction in the ringed brown snake. Table S1-S5. Table S1. Presence and absence of venom gene families in our Australian brown snake species. Table S2. Overview of differential venom gene expression analysis between hatchling and adults venom glands of Eastern brown snakeidentified by DESeq2 algorithm. Table S3. Overview of differential venom gene expression analysis between hatchling and adults venom glands of strap-snouted brown snakeidentified by DESeq2 algorithm. Table S4. Overview of differential venom gene expression analysis between hatchling and adults venom glands of ringed brown snakeidentified by DESeq2 algorithm. Table S5. Overview of *venom and tissue *samples included in this study. Table S6. Overview of snout–vent length data for ancestral state estimation analysis.

Supplementary Material 2. Original, uncropped gel image of prothrombin cleavage profiles for 4-month venoms

Supplementary Material 3. Original, uncropped gel image of prothrombin cleavage profiles for 6-month venoms

Supplementary Material 4. Original, uncropped gel image of prothrombin cleavage profiles for 12-month venoms

## Data Availability

All data generated or analysed during this study are included in this published article, its supplementary information files and publicly available repositories. The raw RNA sequencing (RNA-seq) data have been deposited in the National Center for Biotechnology Information (NCBI) Sequence Read Archive under the BioProject ID PRJNA1249225. The mass spectrometry proteomics data have been deposited to the ProteomeXchange Consortium via the PRIDE partner repository with the dataset identifier PXD063001.

## Abbreviations

3FTxs: Three-finger toxins
BPP: Bayesian posterior probability
CI: Confidence interval
HPLC: High-performance liquid chromatography
KTSPI: Kunitz-type serine protease inhibitors
LC–MS/MS: High-performance liquid chromatography coupled with high-resolution tandem mass spectrometry
PLA₂: Group I phospholipase A₂
SVL: Snout–vent length
UFBoot: Ultrafast bootstrap approximation
vFXa: Venom factor Xa
vFVa: Venom factor Va
