# Supplementary material for: A developmental shift in venom toxin expression underpins a major prey switch in Australian brown snakes
Source: BMC Biol. 2026 Jul 25;24:222. doi: 10.1186/s12915-026-02699-0 (PMC13700484; doi:10.1186/s12915-026-02699-0)
Supplement: Supplementary file 1 [file 12915_2026_2699_MOESM1_ESM.pdf]

Supplementary file 1

A developmental shift in venom toxin expression underpins a major prey switch in Australian brown snakes

Jory van Thiel, Cara F. Smith, Taline D. Kazandjian, Rohit N. Patel, India C. Cullen, Stephanie French, Rachael Da Silva, Laura-Oana Albulescu, Nathan Dunstan, Jeroen Kool, Michael K. Richardson, Stephen P. Mackessy, Nicholas R. Casewell, R. Manjunatha Kini, Anthony J. Saviola, Timothy N.W. Jackson[1], and Cassandra M. Modahl

This PDF file includes:

Fig. S1 to S25

- Fig. S1. Comparative expression levels of identified venom toxin transcripts in liver and venom gland tissues.
- Fig. S2. Venom factor X sequence alignment.
- Fig. S3. Venom factor V sequence alignment.
- Fig. S4. Group I phospholipase A_2 sequence alignment.
- Fig. S5. Kunitz type serine protease inhibitor sequence alignment.
- Fig. S6. Bayesian inference-derived amino acid phylogeny of factor X sequences.
- Fig. S7. Maximum-likelihood estimation-derived amino acid phylogeny of factor X sequences.
- Fig. S8. Bayesian inference-derived amino acid phylogeny of factor V sequences.
- Fig. S9. Maximum-likelihood estimation-derived amino acid phylogeny of factor V sequences.
- Fig. S10. Bayesian inference-derived phylogeny of group I phospholipase A_2 sequences from elapid venoms.

- Fig. S11. Maximum-likelihood estimation-derived phylogeny of group I phospholipase A_2 sequences from elapid venoms.
- Fig. S12. Bayesian inference-derived phylogeny of Kunitz-type serine protease inhibitor sequences from elapid venoms.
- Fig. S13. Maximum-likelihood estimation-derived phylogeny of Kunitz-type serine protease inhibitor sequences from elapid venoms.
- Fig. S14. Sequence alignment of three-finger toxins with identical, functionally confirmed neurotoxins.
- Fig. S15. Log2FoldChange of procoagulant toxin transcripts in adult venom gland tissue of Australian brown snakes (*Pseudonaja* spp.).
- Fig. S16. Chromatographic venom profiles of Strap-snouted brown snake (*Pseudonaja aspidorhyncha*) individual number 02
- Fig. S17. Comparative chromatographic venom profiles of hatchling *Pseudonaja* species.
- Fig. S18. Comparative expression levels of tissue factor pathway inhibitors in venom glands and livers.
- Fig. S19. Comparative expression levels of antithrombin in venom glands and livers.
- Fig. S20. Comparative expression levels of activated protein C in venom glands and livers.
- Fig. S21. Comparative expression levels of phospholipase A_2 inhibitors in venom glands and livers.
- Fig. S22. Coagulation profiles for all Eastern brown snake (*Pseuodnaja textilis*) venoms included in this study.
- Fig. S23. Coagulation profiles for all strap-snouted brown snake (*Pseudonaja aspidorhyncha*) venoms included in this study.
- Fig. S24. Coagulation profiles for all ringed brown snake (*Pseudonaja modesta*) venoms included in this study.
- Fig. S25. Ancestral state estimation of adult body size in the monophyletic *Oxyuranus-Pseudonaja* clade reveals a significant reduction in the ringed brown snake (*Pseudonaja modesta*).

Table S1-S5

- Table S1. Presence and absence of venom gene families in our Australian brown snake species (*Pseudonaja* spp.).
- Table S2. Overview of differential venom gene expression analysis between hatchling and adults venom glands of Eastern brown snake (*Pseudonaja textilis*) identified by DESeq2 algorithm.
- Table S3. Overview of differential venom gene expression analysis between hatchling and adults venom glands of strap-snouted brown snake (*Pseudonaja aspidorhyncha*) identified by DESeq2 algorithm.
- Table S4. Overview of differential venom gene expression analysis between hatchling and adults venom glands of ringed brown snake (*Pseudonaja modesta*) identified by DESeq2 algorithm.
- Table S5. Overview of *venom and tissue* samples included in this study.
- Table S6. Overview of snout–vent length data for ancestral state estimation analysis.

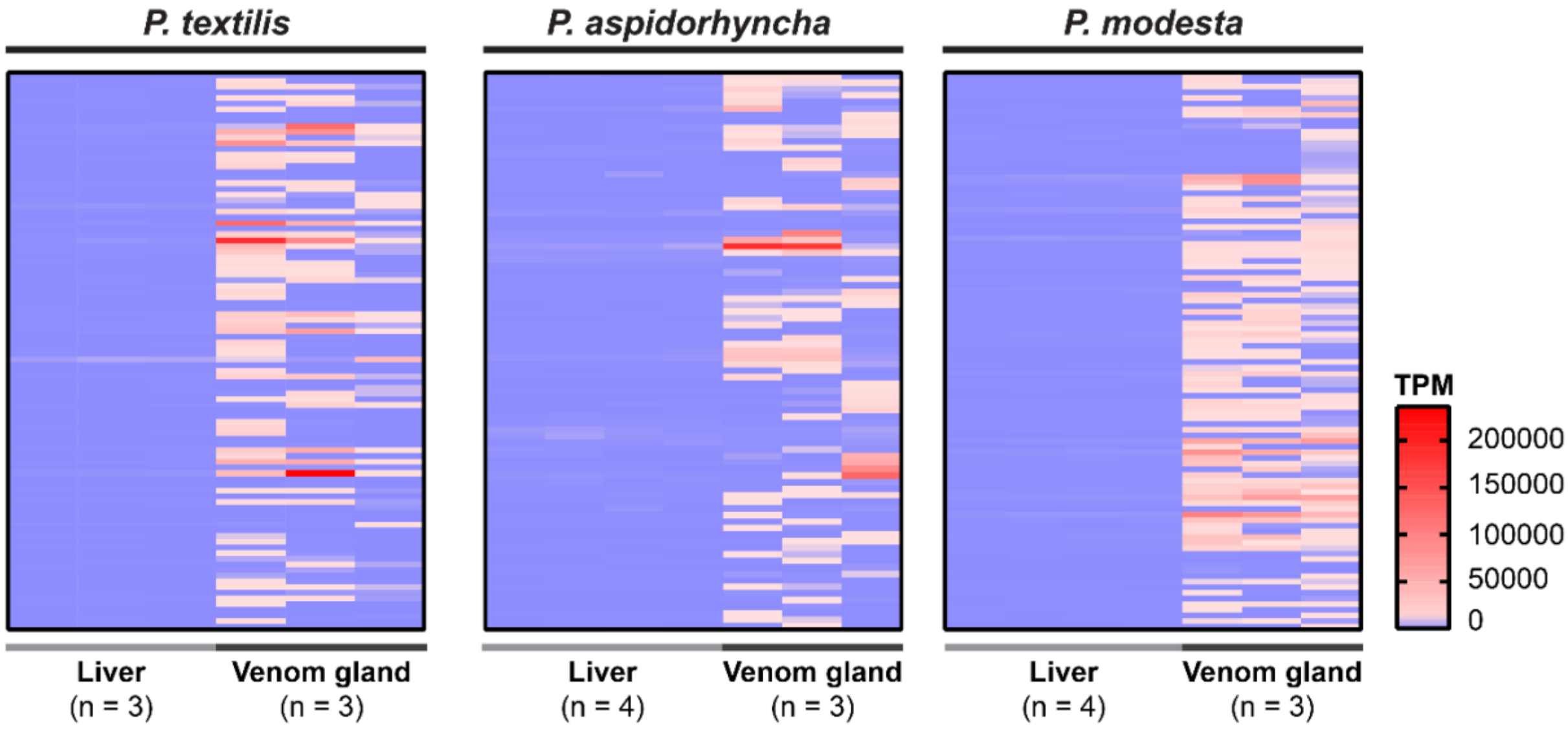

Fig. S1. Comparative expression levels of identified venom toxin transcripts in liver and venom gland tissues. Abbreviation: TPM, transcripts per million.

```
                           10         20         30         40         50         60         70         80
                   ....|....| ....|....| ....|....| ....|....| ....|....| ....|....| ....|....| ....|....|
REF.LFX1.Q1L659    SNVFLKSKVA NRFLQRTKRA NSLFEEFKSG NIERECIEER CSKEEAREAF EDDEKTETFW NVYVDGDQCS SNPCHYGGTC
REF.LFX2.Q1L658    .......... .......... ...V...... .......... .......... .......... .......... ......R.I.
REF.VFX.Q56VR3     .......... .......... ...V...... .......... ........V. .......... .......... ......R.I.
PTE.VFX.ID04       .......... .......... ...V...... .......... ........V. .......... .......... ......R...
PTE.VFX.ID05       .......... .......... ...V...... .......... .......... .......... .......... ......R.I.
PTE.VFX.ID07       .......... .......... ...V...... .......... ........V. .......... .......... ......R...
PTE.VFX.ID06       .......... .......... ...V...... .......... ........V. .......... .......... ......R...
PAS.VFX.ID03       .......... .......... ...V...... .......... .......... .......... .......... ......R.I.
PAS.VFX.ID02       .......... .......... ...V...... .......... .......... .......... .......... ......R.I.
PAS.VFX.ID01       .......... .......... ...V...... .......... .......... .......... .......... ......R.I.
PTE.VFX.ID03       .......... .......... ...V...... .......... .......... .......... .......... ......R.I.
PTE.VFX.ID01       .......... .......... ...V...... .......... .......... .......... .......... ......R.I.
PTE.VFX.ID02       .......... .......... ...V...... .......... .......... .......... .......... ......R.I.
OMI.Q58L95         .......... .......... ......R... .......... ........V. .......... .......... ......R...
OSC.Q58L96         .......... .......... ...Y..R... .......... ........V. .......... .......... ......R...
CNI.B5G6G5         .......... .........S ..I....A.. .........K ........V. ..N....... .......... ......R...
PPO.Q58L93         .......E.. .........S ......RP.. .........K ........I. K.N....A.. .......... ..........
NSC.Q58L94         .......... .........S ......IRP. .........K ........V. ..N....... .......... ......R...
HST.P83370         .......... .........S ......IRP. .........K ........V. ..N....... .......... ......H...
NSC.P82807         .......... .........S ......IRP. .........K ........V. ..N....... .......... ......R...
TCA.P81428         .......... .........S ......IRP. .........K ........V. ..N....... .......... ......R...
DVE.A6MFK7         .......N.. .......... ..G...IYPA .F....V... ........V. .......A.. T........L ..........
DVE.A6MFK8         .......N.. .......... ..I...IRP. ......V..K ........V. Q.N....A.. T........L ......R...

                           90        100        110        120        130        140        150        160
                   ....|....| ....|....| ....|....| ....|....| ....|....| ....|....| ....|....| ....|....|
REF.LFX1.Q1L659    KDGIGSYTCT CLSGYEGKNC EYVLYKSCRV DNGDCWHFCK PVQNGIQCSC AESYLLGEDG HSCVAGGDFS CGRNIKTRNK
REF.LFX2.Q1L658    .......... .......... .R........ ...N...... H...D..... ..G....... .......N.. ..........
REF.VFX.Q56VR3     .......... .......... .R........ ...N...... S...D..... ..G....... .......N.. ..........
PTE.VFX.ID04       .......... .......... .R........ ...N...... S...D..... ..G....... .......N.. ..........
PTE.VFX.ID05       .......... .......... .R........ ...N...... S...D..... ..G....... .......N.. ..........
PTE.VFX.ID07       .......... .......... .R........ ...N...... S...D..... ..G....... .......N.. ..........
PTE.VFX.ID06       .......... .......... .R........ ...N...... S...D..... ..G....... .......N.. ..........
PAS.VFX.ID03       .......... .......... .R........ ...N...... S...D..... ..G....... .......N.. ..........
PAS.VFX.ID02       .......... .......... .R........ ...N...... S...D..... ..G....... .......N.. ..........
PAS.VFX.ID01       .......... .......... .R........ ...N...... S...N..... ..G....... .......N.. ......I...
PTE.VFX.ID03       .......... .......... .R........ ...N...... S...D..... ..G....... .......N.. ..........
PTE.VFX.ID01       .......... .......... .R........ ...N...... S...D..... ..G....... .......N.. ..........
PTE.VFX.ID02       .......... .......... .R........ ...N...... S...D..... ..G....... .......N.. ..........
OMI.Q58L95         .......... ..F....... .R........ ...N...... ....D..... ..G....... .......N.. ..........
OSC.Q58L96         .......... .......... .R........ ...N...... ....D..... ..G....... .......N.. ..........
CNI.B5G6G5         .......... ..PN...... .K..FE.... F..N...... S...E..... ....R..D.. .....E.... ......A...
PPO.Q58L93         .......... ..PN...... .HL.F....F F..N...... ....DT.... ....R..D.. .....E.... ......A...
NSC.Q58L94         .......... ..PN...... .K..F....A F..N...... R..SET.... .......V.. .....E.... ......A...
HST.P83370         .......... ..PN...... .K..F....A F..N...... R..SET.... ....R..V.. .....E.... ......A...
NSC.P82807         .......... ..PN...... .K...Q.... ...N...... R..SET.... ....R..V.. .....E.... ......A...
TCA.P81428         .......... ..PN...... .K...Q.... ...N...... R..SET.... ....R..V.. .....E.... ......A...
DVE.A6MFK7         .......... ..A....... .HD.L..... ...N...... ....DT.... ..G.R..DN. F..I.E.E.. ......S...
DVE.A6MFK8         .......... ..P....... .H.VV....L F..N...... T...DT.... ..G.R..V.. F..I.E.... ...I..S...

                          170        180        190        200        210        220        230        240
                   ....|....| ....|....| ....|....| ....|....| ....|....| ....|....| ....|....| ....|....|
REF.LFX1.Q1L659    REANLPDFQT DFSDDYDEID ENNFVETPTN FSGLVLTVQS QNATLLKKSD NPSPDIRVVN GTDCKLGECP WQALLLNDEG
REF.LFX2.Q1L658    ........-- ---------- ---------- -------... .......... .......I.. .M........ ...A.VDEKE
REF.VFX.Q56VR3     ...S....-- ---------- ---------- -------... H......... .......I.. .M........ ...A.VD.KK
PTE.VFX.ID04       ...S....-- ---------- ---------- -------... .......... .......I.. .M........ ...T.VDEKE
PTE.VFX.ID05       ...S....-- ---------- ---------- -------... .......... .......I.. .M........ ...T.VDEKE
PTE.VFX.ID07       ...S....-- ---------- ---------- -------... .......... .......I.. .M........ ...T.VDEKE
PTE.VFX.ID06       ...S....-- ---------- ---------- -------... .......... .......I.. .M........ ...T.VDEKE
PAS.VFX.ID03       ...S...L-- ---------- ---------- -------... ...P...I.. .......I.. .M........ ...A.VD.KK
PAS.VFX.ID02       ...S....-- ---------- ---------- -------... ...P...I.. .......I.. .M........ ...A.VD.KK
PAS.VFX.ID01       ...S....-- ---------- ---------- -------... ...P...I.. .......I.. .M........ ...A.VD.KK
PTE.VFX.ID03       ...S....-- ---------- ---------- -------... ..TP...I.. .......I.. .M........ ...A.VD.KK
PTE.VFX.ID01       ...S....-- ---------- ---------- -------... ...P...I.. .......I.. .M........ ...A.VD.KK
PTE.VFX.ID02       ...S....-- ---------- ---------- -------... ...P...I.. .......I.. .M........ ...A.VD.KK
OMI.Q58L95         ...S....-- ---------- ---------- -------... ...I...... .......I.. .M........ ...V.VDEKE
OSC.Q58L96         ...S....-- ---------- ---------- -------... ...I...... .......I.. .M........ ...V.VDEKE
CNI.B5G6G5         ...S....-- ---------- ---------- -------... .K.I...... .......II. .M........ ...V..DEDD
PPO.Q58L93         ...S....-- ---------- ---------- -------... .......... .......II. .M........ ...V..DK..
NSC.Q58L94         ...S....-- ---------- ---------- -------... .K..V..... .......I.. .M........ ...V.I.EK.
HST.P83370         ...S....-- ---------- ---------- -------... .K........ .......I.. .M.S...... ...V.I.EK.
NSC.P82807         ...S....-- ---------- ---------- -------... .K........ .......I.. .M........ ...V.I.EK.
TCA.P81428         ...S....-- ---------- ---------- -------... .K........ .......I.. .M........ ...V.I.EK.
DVE.A6MFK7         ...S...... .......A.. ...LI..--- -------... .S........ ..N....I.. .L........ ...V.IDEK.
DVE.A6MFK8         ...S....H- -......A.. ...L...--- -------... .S........ .......I.S .L........ ...V.IDEH.
```

```
                        250        260        270        280        290        300        310        320
                ....|....| ....|....| ....|....| ....|....| ....|....| ....|....| ....|....| ....|....|
REF.LFX1.Q1L659 DGFCGGTILS PIYVLTAAHC INQTKYITVV VGEIDISSKK TGRLHSVDKI YVHQKFVPA- ---------- --TYDYDIAI
REF.LFX2.Q1L658 GV........ .......... ..E.ET.S.. .....K.RIE ..P.L..... ...K....PQ KAY----KFD LAA.......
REF.VFX.Q56VR3  GV........ .......... ..E.ET.S.. .....R.RAE ..P.L....V ...K....PK KSQEFYEKFD LVS.......
PTE.VFX.ID04    GV........ .......... F.E.ET.S.. .....K.RVE ..P.L..... ...K....PK KSQEFYEKFD LVS.......
PTE.VFX.ID05    GV........ .......... F.E.ET.S.. .....K.RVE ..P.L..... ...K....PK RGYKFYEKFD LVS.......
PTE.VFX.ID07    GV........ .......... F.E.ET.S.. .....K.RVE ..P.L....- ----....PK RGYKFYEKFD LVS.......
PTE.VFX.ID06    GV........ .......... F.E.ET.S.. .....K.RVE ..P.L..... ...K....PK RGYKFYEKFD LVS.......
PAS.VFX.ID03    GV........ .......... ..E.ET.S.. .....R.RAE ..P.L....V ...K....PK KSQEFYEKFD LVS.......
PAS.VFX.ID02    GV........ .......... ..E.ET.S.. .....R.RAE ..P.L....V ...K....XK KSQEFYEKFD LVS.......
PAS.VFX.ID01    GV........ .......... ..E.ET.S.. .....R.RAE ..P.L....V ...K....PK KSQEFYEKFD LVS.......
PTE.VFX.ID03    GV........ .......... ..E.ET.S.. .....R.RAE ..P.L....V ...K....PK KSQEFYEKFD LVS.......
PTE.VFX.ID01    GV........ .......... ..E.ET.S.. .....R.RAE ..P.L....V ...K....PK KSQEFYEKFD LVS.......
PTE.VFX.ID02    GV........ .......... ..E.ET.S.. .....R.RAE ..P.L....V ...K....PK KSQEFYEKFD LVS.......
OMI.Q58L95      GV........ .......... ....EK.S.. .....K.RVE ..H.L..... ...K....PK KGYKFYEKFD LVS.......
OSC.Q58L96      .A........ .......... .....M.S.. ....N..R.N P...L..... ........PK KGYEFYEKFD LVS.......
CNI.B5G6G5      .V........ S.H....... .....N.S.I .......R.E .I..LP...V ...T....PS YLY-GHQNVD RK........
PPO.Q58L93      .V........ .......... .T.S.H.S.. .......R.E .RH.L....A ...T...L.- ---------- --........
NSC.Q58L94      EV........ ..H....... .....SVS.I .......R.E .R..L..... ...K....P- --NSYYQNID RFA.......
HST.P83370      EV........ ..H....... .....SVS.I .......R.E .R..L..... ...T....PN YYY-GHQNFD RVA.......
NSC.P82807      EV........ ..H....... .....SVS.I .......R.E .R..L..... ...T....PN YYY-VHQNFD RVA.......
TCA.P81428      EV........ ..H....... .....SVS.I .......R.E .R..L..... ...T....PN YYY-VHQNFD RVA.......
DVE.A6MFK7      TA.G...... .YF....... ..K..S.A.. ..QV...R.E .R..L....V .T.P.Y.HV- ---------- --.N......
DVE.A6MFK8      KA.G...... .YF....... L....S.A.. ..QV...R.E .RH.LH...A .M.S.Y.R.- ---------- --...H....

                        330        340        350        360        370        380        390        400
                ....|....| ....|....| ....|....| ....|....| ....|....| ....|....| ....|....| ....|....|
REF.LFX1.Q1L659 IQLKT-PIQF SENVVPACLP TADFANQVLM KQNFGIVSGF GRTRERGKTS NTLKVVTLPY VDRHTCMLSS NFPITQNMFC
REF.LFX2.Q1L658 ..M..-.... .......... .......... ..D....... ..IF.K.PK. K....LKV.. .......V.. ET...P....
REF.VFX.Q56VR3  ..M..S.... .......... .......... ..D....... .GIF...PN. K....LKV.. .......... .....PT...
PTE.VFX.ID04    .....S.... .......... .......... ..D....... .HIF...PK. K....LKV.. .......... ..........
PTE.VFX.ID05    .....S.... .......... .......... ..D....... .HIF...PK. K....LKV.. .......... ..........
PTE.VFX.ID07    .....S.... .......... .......... ..D....... .HIF...PK. K....LKV.. .......... ..........
PTE.VFX.ID06    .....S.... .......... .......... ..D....... .HIF...PK. K....LKV.. .......... ..........
PAS.VFX.ID03    .....S.... .......... .......... ..D....... .GIF...PN. K....LKV.. .......V.. ET...PT...
PAS.VFX.ID02    .....S.... .......... .......... ..D....... .GIF...PN. K....LKV.. .......V.. ET...PT...
PAS.VFX.ID01    .....S.... .......... .......... ..D....... .GIF...PN. K....LKV.. .......V.. ET...PT...
PTE.VFX.ID03    ..M..S.... .......... .......... ..D....... .GIF...PN. K....LKV.. .......... .....PT...
PTE.VFX.ID01    ..M..S.... .......... .......... ..D....... .GIF...PN. K....LKV.. .......... .....PT...
PTE.VFX.ID02    ..M..S.... .......... .......... ..D....... .GIF...PN. K....LKV.. .......... .....PT...
OMI.Q58L95      ..M..-.... .......... .......... ..D..I.... ..IF.K.PK. .....LKV.. .......V.. ES...PT...
OSC.Q58L96      L.M..-.... .......... .......... ..D....... ..IF.K.PQ. K....LKV.. .......... ES...PT...
CNI.B5G6G5      .RM..-.... .......... ....VK.... ..D....... ...QY..Q.. .....I.V.. ...R...... D....P....
PPO.Q58L93      .....-.... .......... .......... ..D..I.... .H..SG.Q.. .......I.. .......... D.R..P....
NSC.Q58L94      .RM..-.... .......... .....KE... ..DS...... ...QSI.Y.. .I...I.V.. .......... ..R.......
HST.P83370      .RM..-.... .......... ......E... ..DS...... ..I.FKEP.. .....I.V.. .......... D.R.......
NSC.P82807      .RM..-.... .......... ......E... ..DS...... ..I.FKEP.. .....I.V.. .......... D.R.......
TCA.P81428      .RM..-.... .......... ......E... ..DS...... ..IQFKQP.. .....I.V.. .......... D.R.......
DVE.A6MFK7      .....-.... .......... ......H... ..D....... ..IE.K.P.. .I....MV.. ......I..T KI...R....
DVE.A6MFK8      LR.R.-.... .......... .....DE... ..D....... ..LH...S.. DI...IRV.. ...Y...... .YR..PS...

                        410        420        430        440        450        460        470
                ....|....| ....|....| ....|....| ....|....| ....|....| ....|....| ....|....| ....|.
REF.LFX1.Q1L659 AGYDTLPQDA CQGDSGGPHI TAYRDTHFIT GIVSWGEGCA QTGKYGVYTK VSKFILWIKR IIRQKQPSTE SSTGRL Liver
REF.LFX2.Q1L658 .......R.. .........T .V........ ....S..... RN...I.... L....P.... .M...L.... ...... Liver
REF.VFX.Q56VR3  .......... .......... .......... .......... RK.R..I... L....P.... .M...L.... ...... Venom
PTE.VFX.ID04    .......... .......... .......... .......... .......... .....P.... .M...L.... ...... 95.75%
PTE.VFX.ID05    .......... .......... .......... .......... .......... .....P.... .M...L.... ...... 94.85%
PTE.VFX.ID07    .......... .......... .......... .......... .......... .....P.... .M...L.... ...... 93.96%
PTE.VFX.ID06    .......... .......... .......... .......... .......... .....P.... .M...L.... ...... 94.85%
PAS.VFX.ID03    .......R.. .......... .......... .......... RK.R..I... L....P.... .M...L.... ...... 97.76%
PAS.VFX.ID02    .......R.. .......... .......... .......... RK.R..I... L....P.... .M...L.... ...... 97.76%
PAS.VFX.ID01    .......R.. .......... .......... .......... RK.R..I... L....P.... .M...L.... ...... 97.54%
PTE.VFX.ID03    .......... .......... .......... .......... RK.R..I... L....P.... .M...L.... ...... 98.88%
PTE.VFX.ID01    .......... .....X.... .......... .......... RK.R..I... L....P.... .M...L.... ...... 98.88%
PTE.VFX.ID02    .......... .......... .......... .......... RK.R..I... L....P.... .M...L.... ...... 99.11%
OMI.Q58L95      .......R.. .......... .......... .......... KK....I... .......... .M...L.... ...... 79.79%
OSC.Q58L96      .......R.. .......... .......... .......... .......... .......... .M...L.... ...... 81.05%
CNI.B5G6G5      .......... .......... ...G...... ..N....... KK........ ..N..P...A .MSL.----- ------ 74.68%
PPO.Q58L93      .......R.. .......... .......... ..I....... KK........ ..N..P...A VM.KH..... ...... 79.87%
NSC.Q58L94      .......... .......... ...G....V. ..I....... RK........ ..N..P...K .MSL.----- ------ 74.58%
HST.P83370      .......... .E........ ...G...... .......... RK........ ..R..P...K .MSL.----- ------ 74.58%
NSC.P82807      .......... .......... .......... ..I....... RK........ ..R..P...K .MSL.----- ------ 75.11%
TCA.P81428      .......... .......... .......... ..I....... RK........ .....P...K .MSL.----- ------ 75.11%
DVE.A6MFK7      ...GNQ.E.. .E........ ...K....L. .........G RD....I... ..N.LP...T .M.R...... ...... 77.71%
DVE.A6MFK8      ...GNQ.... .......... ...G...... ..I......G RK....I... ..N..P...T .M.RN..... ...... 75.54%
```

Fig. S2. Venom factor X sequence alignment. Our de novo transcripts were aligned with reference sequences from *Pseudonaja textilis* and other Australian elapid snakes. In the sequence alignment, dots (.) indicate identical residues with the reference (UniProt ID: Q1L659), while dashes (-) represent gaps. The percentages reflect the similarity between our transcripts and the *P. textilis* venom isoform (UniProt ID: Q56VR3), which was used as the reference.

```
                        10         20         30         40         50         60         70         80
                ....|....| ....|....| ....|....| ....|....| ....|....| ....|....| ....|....| ....|....|
REF.LFV.Q593B6  AQLREYHIAA QLEDWDYNPQ PEELSRLSES DLTFKKIVYR EYELDFKQEK PRDELSGLLG PTLRGEVGDI LIIYFKNFAT
REF.VFV.Q7SZN0  .......... .......... .......... .......... .........E ...A...... .........S ..........
PTE.VFV.ID01    .......... .......... .......... .......... .......... ...A...... .........S ..........
PTE.VFV.ID02    .......... .......... .......... .......... .......... ...A...... .........S ..........
PTE.VFV.ID03    .......... .......... .......... .......... .......... ...A...... .........S ..........
PTE.VFV.ID04    .......... .......... .......... .......... .......... ...A...... .........S ..........
PTE.VFV.ID05    .......... .......... .......... .......... .......... ...A...... .........S ..........
PAS.VFV.ID01    .......... .......... ....P...K. .......... .......... ...A...... .........S ..........
PAS.VFV.ID02    .......... .......... ....P...K. .......... .......... ...A...... .........S ..........
PAS.VFV.ID03    .......... .......... ....P...K. .......... .......... ...A...... .........S ..........
PAS.VFV.ID04    .......... .......... ....P...K. .......... .......... ...A...... .........S ..........
OMI.Q58L90      ......R... .......... .......... E......... .......... .......... .......... ..........
OSC.Q58L91      ......RL.. .......... .......... .......... .......... .......... .........S ..........
OSC.AAY47065    ......R... .......... .......... .......... .......... .......... .........S ..........

                        90        100        110        120        130        140        150        160
                ....|....| ....|....| ....|....| ....|....| ....|....| ....|....| ....|....| ....|....|
REF.LFV.Q593B6  QPVSIHPQSA VYNKWSEGSS YSDGTSDVER LDDAVPPGQS FKYVWNITAE IGPKKADPPC LTYAYYSHVN MVRDFNSGLI
REF.VFV.Q7SZN0  .......... .......... .......... .......... .......... .......... .......... ..........
PTE.VFV.ID01    .......... .......... .......... .......... .......... .......... .......... ..........
PTE.VFV.ID02    .......... .......... .......... .......... .......... .......... .......... ..........
PTE.VFV.ID03    .......... .......... .......... .......... .......... .......... .......... ..........
PTE.VFV.ID04    .......... .......... .......... .......... .......... .......... .......... ..........
PTE.VFV.ID05    .......... .......... .......... .......... .......... .......... .......... ..........
PAS.VFV.ID01    .......... M......... .......... .......... .......... .......... .......... ..........
PAS.VFV.ID02    .......... M......... .......... .......... .......... .......... .......... ..........
PAS.VFV.ID03    .......... .......... .......... .......... .......... .......... .......... ..........
PAS.VFV.ID04    .......... .......... .......... .......... .......... .......... .......... ..........
OMI.Q58L90      .......... .......... .......... .......... .......... .......... .......... ..........
OSC.Q58L91      .......... .......... .......... .......... .......... .......... .......... ..........
OSC.AAY47065    .......... .......... .......... .......... .......... .......... .......... ..........

                       170        180        190        200        210        220        230        240
                ....|....| ....|....| ....|....| ....|....| ....|....| ....|....| ....|....| ....|....|
REF.LFV.Q593B6  GALLICKEGS LNANGSQKFF NREYVLMFSV FDESKNWYRK PSLQYTINGF ANGTLPDVQA CAYDHISWHL IGMSSSPEIF
REF.VFV.Q7SZN0  .......... .......... .......... .......... .......... .......... .......... ..........
PTE.VFV.ID01    .......... .......... .......... .......... .......... .......... ......T... ..........
PTE.VFV.ID02    .......... .......... .......... .......... .......... .......... ......T... ..........
PTE.VFV.ID03    .......K.. .....A.... .......... .......... .......... .......... ......T... ..........
PTE.VFV.ID04    .......K.. .....A.... .......... .......... .......... .......... ......T... ..........
PTE.VFV.ID05    .......K.. .....A.... .......... .......... .......... .......... ......T... ..........
PAS.VFV.ID01    .......K.. .....A.... .......... .......... .......... .......... .......... ..........
PAS.VFV.ID02    .......K.. .....A.... .......... .......... .......... .......... .......... ..........
PAS.VFV.ID03    .......K.. .....A.... .......... .......... .......... .......... .......... ..........
PAS.VFV.ID04    .......K.. .....A.... .......... .......... .......... .......... .......... ..........
OMI.Q58L90      .......... .....A.... .......... .......... .......... .......... .......... ..........
OSC.Q58L91      .......... ...D.A.... .......... .......... .......... .......... .......... ..........
OSC.AAY47065    .......... ...D.A.... .......... .......... .......... .......... .......... ..........

                       250        260        270        280        290        300        310        320
                ....|....| ....|....| ....|....| ....|....| ....|....| ....|....| ....|....| ....|....|
REF.LFV.Q593B6  SVHFNGQTLE QNHYKVSTIN LVGGASVTAN MSVSRTGKWL ISSLVAKHLQ AGMYGYLNIK DCGNPDTLTR KLSFRELRRI
REF.VFV.Q7SZN0  .......... .......... .........D .......... .......... .......... .......... .......MK.
PTE.VFV.ID01    .......... .......... .......... .......... .......... .......... .......... .......MK.
PTE.VFV.ID02    .......... .......... .......... .......... .......... .......... .......... .......MK.
PTE.VFV.ID03    .......... .......... .......... .......... .......... .......... .......... ...LKQW.K.
PTE.VFV.ID04    .......... .......... .......... .......... .......... .......... .......... ...LKQW.K.
PTE.VFV.ID05    .......... .......... .......... .......... .......... .......... .......... ...LKQW.K.
PAS.VFV.ID01    .......... .......... .......... .......... .......... .......... .......... ...LKQR.K.
PAS.VFV.ID02    .......... .......... .......... .......... .......... .......... .......... ...LKQR.K.
PAS.VFV.ID03    .......... .......... .......... .......... .......... .......... .......... ...LKQR.K.
PAS.VFV.ID04    .......... .......... .......... .......... .......... .......... .......... ...LKQR.K.
OMI.Q58L90      .......... .......... .......... .......... .......... .......... ...H.N.... ..........
OSC.Q58L91      .......... .......... .......... .......... .......... .......... .......... ......R...
OSC.AAY47065    .......... .......... .......... .......... .......... .......... .......... ......W...

                       330        340        350        360        370        380        390        400
                ....|....| ....|....| ....|....| ....|....| ....|....| ....|....| ....|....| ....|....|
REF.LFV.Q593B6  MNWEYFIAAE EITWDYAPEI PSSVDRRYKA QYLDNFSNFI GKKYKKAVFR QYKDSNFTKP TYAIWPKERG ILGPVIRAKV
REF.VFV.Q7SZN0  K......... .......... .......... .......... .......... ..E.G..... .......... ......K...
PTE.VFV.ID01    K......... .......... .......... .......... .......... ..E.G..... ...T...... ......K...
PTE.VFV.ID02    K......... .......... .......... .......... .......... ..E.G..... ...T...... ......K...
PTE.VFV.ID03    K......... .......... .....S.... .......... .......... ..E.G..... ...T...... ......K...
PTE.VFV.ID04    K......... .......... .....S.... .......... .......... ..E.G..... ...T...... ......K...
PTE.VFV.ID05    K......... .......... .....S.... .......... .......... ..E.G..... ...T...... ......K...
PAS.VFV.ID01    K......... .......... .....S.... .......... .......... ..E.G..... .......... ......K...
PAS.VFV.ID02    K......... .......... .....S.... .......... .......... ..E.G..... .......... ......K...
PAS.VFV.ID03    K......... .......... .....S.... .......... .......... ..E.G..... .......... ......K...
PAS.VFV.ID04    K......... .......... .....S.... .......... .......... ..E.G..... .......... ......K...
OMI.Q58L90      .......... .......... .......... .......... .......... ..E.G..... .......... ......K...
OSC.Q58L91      .K........ .......... .......... .......... .......... ..E....... .......... ......K...
OSC.AAY47065    .K........ .......... .......... ....-..... .......... ..E....... .......... ......K...
```

```
                       410        420        430        440        450        460        470        480
                ....|....| ....|....| ....|....| ....|....| ....|....| ....|....| ....|....| ....|....|
REF.LFV.Q593B6  RDTISIVFKN LASRPYSIYV HGVSVSKDAE GAIYPSDPKE NITHGKAVEP GQVYTYKWTV LDTDEPTVKD SECITKLYHS
REF.VFV.Q7SZN0  ...VT..... .......... .......... .......... .......... .......... .......... ..........
PTE.VFV.ID01    ...VT..... .......... .......... .......... .......... .......... .......... ..........
PTE.VFV.ID02    ...VT..... .......... .......... .......... .......... .......... .......... ..........
PTE.VFV.ID03    ...VT..... .......... .......... .......... .......... .......... .......... ..........
PTE.VFV.ID04    ...VT..... .......... .......... .......... .......... .......... .......... ..........
PTE.VFV.ID05    ...VT..... .......... .......... .......... .......... .......... .......... ..........
PAS.VFV.ID01    ...VT..... .......... .......... .......... ....D..... .......... .......... ..........
PAS.VFV.ID02    ...VT..... .......... .......... .......... ....D..... .......... .......... ..........
PAS.VFV.ID03    ...VT..... .......... .......... .......... .......... .......... .......... ..........
PAS.VFV.ID04    ...VT..... .......... .......... .......... .......... .......... .......... ..........
OMI.Q58L90      ...VT..... .......... .......... .......... .......... .......... .......... ..........
OSC.Q58L91      ...VT..... .......... .......... ..V....... .......... .......... .......... ..........
OSC.AAY47065    ...VT..... .......... .......... ..V....... .......... .......... .......... ..........

                       490        500        510        520        530        540        550        560
                ....|....| ....|....| ....|....| ....|....| ....|....| ....|....| ....|....| ....|....|
REF.LFV.Q593B6  AVDMTRDIAS GLIGPLLVCK HKALSVKGVQ NKADVEQHAV FAVFDENKSW YLEDNIKKYC SNPSTVKKDD PKFYKSNVMY
REF.VFV.Q7SZN0  .......... .......... .......... .......... .......... .......... ....A..... ..........
PTE.VFV.ID01    .......... .......... .......... .......... .......... .......... .......... ..........
PTE.VFV.ID02    .......... .......... .......... .......... .......... .......... .......... ..........
PTE.VFV.ID03    .......... .......... R......... .S........ .......... .......... .......... ..........
PTE.VFV.ID04    .......... .......... R......... .S........ .......... .......... .......... ..........
PTE.VFV.ID05    .......... .......... R......... .S........ .......... .......... .......... ..........
PAS.VFV.ID01    .......... .......... R......... .S........ .......... .......... .......... ..........
PAS.VFV.ID02    .......... .......... R......... .S........ .......... .......... .......... ..........
PAS.VFV.ID03    .......... .......... R......... .S........ .......... .......... .......... ..........
PAS.VFV.ID04    .......... .......... R......... .S........ .......... .......... .......... ..........
OMI.Q58L90      .......... .......... L......... .......... .......... .......... ....S..... ..........
OSC.Q58L91      .......... .....P.... R....IR... .......... .......... .......... ....S..... ..........
OSC.AAY47065    .......... .......... R....IR... .......... .......... .......... ....S..... ..........

                       570        580        590        600        610        620        630        640
                ....|....| ....|....| ....|....| ....|....| ....|....| ....|....| ....|....| ....|....|
REF.LFV.Q593B6  TLNGYASDRT EVLGFHQSEV VEWHLTSVGT VDEIVPVHLS GHTFLSKGKH QDILNLFPMS GESATVTMDN LGTWLLSSWG
REF.VFV.Q7SZN0  .......... ...R...... .Q........ .......... .......... .......... .......... ..........
PTE.VFV.ID01    .......... ...R...... .Q........ .......... .......... .......... .......... ..........
PTE.VFV.ID02    .......... ...R...... .Q........ .......... .......... .......... .......... ..........
PTE.VFV.ID03    .......... ...R...... .Q........ .......... .......... .......... .......... ..........
PTE.VFV.ID04    .......... ...R...... .Q........ .......... .......... .......... .......... ..........
PTE.VFV.ID05    .......... ...R...... .Q........ .......... .......... .......... .......... ..........
PAS.VFV.ID01    .......... ...R...... .Q........ .......... .......... .......... .......... ..........
PAS.VFV.ID02    .......... ...R...... .Q........ .......... .......... .......... .......... ..........
PAS.VFV.ID03    .......... ...R...... .Q........ .......... .......... .......... .......... ..........
PAS.VFV.ID04    .......... ...R...... .Q........ .......... .......... .......... .......... ..........
OMI.Q58L90      .......... .......... .Q........ .......... .......... .......... .......... ..........
OSC.Q58L91      .......... ..W....... .......... .......... .......... .......... .......... ..........
OSC.AAY47065    .......... ..W....... .......... .......... .......... .......... .......... ..........

                       650        660        670        680        690        700        710        720
                ....|....| ....|....| ....|....| ....|....| ....|....| ....|....| ....|....| ....|....|
REF.LFV.Q593B6  SCEMSNGMRL RFLDANYDDE DEGNEEEEED DGDIFADIFI PPEVVKKKEK DPVNFVSDPE SDKIAKELGL LDDEDNQ-EE
REF.VFV.Q7SZN0  .......... .......... .......... .......... .S.......E V.....P... ..AL...... I...G.PIIQ
PTE.VFV.ID01    .......... .......... .......... .......... .S.......E V.....P... ..AL...... I...G.PIIQ
PTE.VFV.ID02    .......... .......... .......... .......... .S.......E V.....P... ..AL...... I...G.PIIQ
PTE.VFV.ID03    .......... .......... .......... .......... .S.......E V.....P... ..AL...... I...G.PIIQ
PTE.VFV.ID04    .......... .......... .......... .......... .S.......E V.....P... ..AL...... I...G.PIIQ
PTE.VFV.ID05    .......... .......... .......... .......... .S.......E V.....P... ..AL...... I...G.PIIQ
PAS.VFV.ID01    .......... .......... .......... .......... .S.......E V.....P... ..AL...... I...G.PIIQ
PAS.VFV.ID02    .......... .......... .......... .......... .S.......E V.....P... ..AL...... I...G.PIIQ
PAS.VFV.ID03    .......... .......... .......... .......... .S.......E V.....P... .EAL...... I...G.PIIQ
PAS.VFV.ID04    .......... .......... .......... .......... .S.......E V.....P... .EAL...... I...G.PIIQ
OMI.Q58L90      .......... .......... .......... .........S .........E V.....P... ..AL...... ......P-.Q
OSC.Q58L91      .......... .......... .......... .........N .....I...E V.....P... ..AL...... F.....P-KQ
OSC.AAY47065    .......... .......... .......... .........N .....I...E V.....P... ..AL...... F.....P-KQ

                       730        740        750        760        770        780        790        800
                ....|....| ....|....| ....|....| ....|....| ....|....| ....|....| ....|....| ....|....|
REF.LFV.Q593B6  SHNVQTEDDE EQLMIATMLG FRSFKGSVAE EELNLTALAL EEDAHASDPR IDSNSARNPD DIAGRYLRTI NRGNKRRYYI
REF.VFV.Q7SZN0  PRRE...... ....K.S... L......... ...KH..... .......... .......... .......... ..........
PTE.VFV.ID01    PRRE...... ....K.S... L......... ...KH..... .......... .......... .......... ..........
PTE.VFV.ID02    PRRE...... ....K.S... L......... ...KH..... .......... .......... .......... ..........
PTE.VFV.ID03    PRRE...... ....K.S... L......... ...KH..... .......... .......... .......... ..........
PTE.VFV.ID04    PRRE...... ....K.S... L......... ...KH..... .......... .......... .......... ..........
PTE.VFV.ID05    PRRE...... ....K.S... L......... ...KH..... .......... .......... .......... ..........
PAS.VFV.ID01    PRRE...... ....K.S... L......... ...KH..... .......... .......... .......... ..........
PAS.VFV.ID02    PRRE...... ....K.S... L......... ...KH..... .......... .......... .......... ..........
PAS.VFV.ID03    PRRE...... ....K.S... L......... ...KH..... .......... ..R....... .......... ..........
PAS.VFV.ID04    PRRE...... ....K.S... L......... ...KH..... .......... ..R....... .......... ..........
OMI.Q58L90      .RSE...... ......SV.. L......... ...KH..... .......... ........S. .......... ..R.......
OSC.Q58L91      .RSE...... ......S... L......... ...KH..... .......... ......H.S. .......... ..R.......
OSC.AAY47065    .RSE...... ......S... L......... ...KH..... .......... ......H.S. .......... Y.R.......
```

```
                   810        820        830        840        850        860        870        880
                   ....|....| ....|....| ....|....| ....|....| ....|....| ....|....| ....|....| ....|....|
REF.LFV.Q593B6     AAEEVLWDYS PIG------- ---------- ---------- -------KSQ VRSRAAKTTF KKAIFRSYLD DTFQTPSTGG
REF.VFV.Q7SZN0     .......... ...------- ---------- ---------- -------... .......... .......... ..........
PTE.VFV.ID01       .......... ...------- ---------- ---------- -------... .......... .......... ..........
PTE.VFV.ID02       .......... ...KSQKLFW KIEESELESR KRIEKDKYIY SEENIKK... .......... .......... ..........
PTE.VFV.ID03       .......... ...KSQKLFW KIEESELESR KRIEKDKYIY SEENIKK... .......... .......... ..........
PTE.VFV.ID04       .......... ...------- ---------- ---------- -------... .......... .......... ..........
PTE.VFV.ID05       .......... ...------- ---------- ---------- -------... .......... .......... ..........
PAS.VFV.ID01       .......... ...------- ---------- ---------- -------... .......... .......... ..........
PAS.VFV.ID02       .......... ...KSQKLFW KIEESELESR KRIEKDKYIY SEENIKK... .......... .......... ..........
PAS.VFV.ID03       .......... ...------- ---------- ---------- -------... .......... .......... ..........
PAS.VFV.ID04       .......... ...KSQKLFW KIEESELESR KRIEKDKYIY SEENIKK... .......... .......... ..........
OMI.Q58L90         .......... ...------- ---------- ---------- -------... ...LP..... .......... ..........
OSC.Q58L91         .......... ...------- ---------- ---------- -------... ...LP..... .......... ..........
OSC.AAY47065       .......... ...------- ---------- ---------- -------... ...LP..... .......... ..........

                   890        900        910        920        930        940        950        960
                   ....|....| ....|....| ....|....| ....|....| ....|....| ....|....| ....|....| ....|....|
REF.LFV.Q593B6     EYEKHLGILG PIIRAEVDDV IEVQFRNLAS RPYSLHAHGL LYEKSSEGRS YDDKSPELFK KDDAIMPNGT YTYVWQVPPR
REF.VFV.Q7SZN0     .......... .......... ..I..K.... .......... .......... .......... .......... ..........
PTE.VFV.ID01       .......... .......... ..I....... .......... .......... .......... .......... ..........
PTE.VFV.ID02       .......... .......... ..I....... .......... .......... .......... .......... ..........
PTE.VFV.ID03       .......... .......... ..I....... .......... .......... .......... .......... ..........
PTE.VFV.ID04       .......... .......... ..I....... .......... .......... .......... .......... ..........
PTE.VFV.ID05       .......... .......... ..I....... .......... .......... .......... .......... ..........
PAS.VFV.ID01       .......... .......... ..I....... .......... .......... .......... .......... ..........
PAS.VFV.ID02       .......... .......... ..I....... .......... .......... .......... .......... ..........
PAS.VFV.ID03       .......... .......... ..I....... .......... .......... .......... .......... ..........
PAS.VFV.ID04       .......... .......... ..I....... .......... .......... .......... .......... ..........
OMI.Q58L90         .......... .......... .......... .......... .......... ...N...... .......... ..........
OSC.Q58L91         .......... .......... .......... .......... .......... ...N...... .......... ..........
OSC.AAY47065       .......... .......... .......... .......... .......... ...N...... .......... ..........

                   970        980        990        1000       1110       1120       1130       1140
                   ....|....| ....|....| ....|....| ....|....| ....|....| ....|....| ....|....| ....|....|
REF.LFV.Q593B6     SGPTDNTEKC KSWAYYSGVN PEKDIHSGLI GPILICQKGM IDKYNRTIDI REFVLFFMVF DEEKSWYFPK SDKSTRAEKL
REF.VFV.Q7SZN0     .......... .......... .......... .......... .......... .......... .......... .....CE...
PTE.VFV.ID01       .......... .......... .......... .......... .......... .......... .......... .....CE...
PTE.VFV.ID02       .......... .......... .......... .......... .......... .......... .......... .....CE...
PTE.VFV.ID03       .......... .......... .......... .......... .......... .......... .......... .....CE...
PTE.VFV.ID04       .......... .......... .......... .......... .......... .......... .......... .....CE...
PTE.VFV.ID05       .......... .......... .......... .......... .......... .......... .......... .....CE...
PAS.VFV.ID01       .......... .......... .......... .......... .......... .......... .......... .....CE...
PAS.VFV.ID02       .......... .......... .......... .......... .......... .......... .......... .....CE...
PAS.VFV.ID03       .......... .......... .......... .......... .......... .......... .......... .....CE...
PAS.VFV.ID04       .......... .......... .......... .......... .......... .......... .......... .....CE...
OMI.Q58L90         .......... .......... .......... .......... .......... .......... .......... .....CE...
OSC.Q58L91         .......... .......... .......... .......... .......... .......... .......... .....CE...
OSC.AAY47065       .......... .......... .......... .......... .......... .......... .......... .....CE...

                   1150       1160       1170       1180       1190       1200       1210       1220
                   ....|....| ....|....| ....|....| ....|....| ....|....| ....|....| ....|....| ....|....|
REF.LFV.Q593B6     IGVQ-SRHTF PAINGIPYQL QGLTMYKDEN VHWHLLNMGG PKDIHVVNFH GQTFTEEGRE DNQLGVLPLL PGTFASIKMK
REF.VFV.Q7SZN0     ....-.L... .......... .......... .......... .......... .......... .......... ..........
PTE.VFV.ID01       ....-.L... .......... .......... .......... .......... .......... .......... ..........
PTE.VFV.ID02       ....-.L... .......... .......... .......... .......... .......... .......... ..........
PTE.VFV.ID03       ....-.L... .......... .......... .......... .......... .......... .Y........ ..........
PTE.VFV.ID04       ....-.L... .......... .......... .......... .......... .......... .Y........ ..........
PTE.VFV.ID05       ....-.L... .......... .......... .......... .......... .......... .Y........ ..........
PAS.VFV.ID01       ...R-.L... .......... .......... .......... .......... .......... .Y........ ..........
PAS.VFV.ID02       ...R-.L... .......... .......... .......... .......... .......... .Y........ ..........
PAS.VFV.ID03       ...R-.L... .......... .......... .......... .......... .......... .Y........ ..........
PAS.VFV.ID04       ...R-.L... .......... .......... .......... .......... .......... .Y........ ..........
OMI.Q58L90         ....S.H... .......... ..M....... .......... .......... .......... .......... ..........
OSC.Q58L91         ....-..... .......... ..M....... .......... ...V...... .......... .......... ..........
OSC.AAY47065       ....-..... .......... ..M....... .......... ...V...... .......... .......... ..........

                   1230       1240       1250       1260       1270       1280       1290       1300
                   ....|....| ....|....| ....|....| ....|....| ....|....| ....|....| ....|....| ....|....|
REF.LFV.Q593B6     PSKIGTWLLE TEVGENQERG MQALFTVIDK DCKLPMGLAS GIIQDSQISA SGHVGYWEPK LARLNNTGKY NAWSIIKKEH
REF.VFV.Q7SZN0     .......... .......... .......... .......... .......... .......... .......... ..........
PTE.VFV.ID01       .......... .......... .......... .......... .......... .......... .......AIF ..........
PTE.VFV.ID02       .......... .......... .......... .......... .......... .......... .......AIF ..........
PTE.VFV.ID03       .......... .......... .......... .......... .......... .......... .......AIF ..........
PTE.VFV.ID04       .......... .......... .......... .......... .......... .......... .......AIF ..........
PTE.VFV.ID05       .......... .......... .......... .......... .......... .......... .......AIF ..........
PAS.VFV.ID01       .......... .......... .......... .......... .......... .......... .......AIF ..........
PAS.VFV.ID02       .......... .......... .......... .......... .......... .......... .......AIF ..........
PAS.VFV.ID03       .......... .......... .......... .......... .......... .......... .....S.AIF ..........
PAS.VFV.ID04       .......... .......... .......... .......... .......... .......... .....S.AIF ..........
OMI.Q58L90         .......... .......... .......... .......... .......... ....E..... ........MF ..........
OSC.Q58L91         .......... .......... .......... .......... .......... .......... ........MF ..........
OSC.AAY47065       .......... .......... .......... .......... .......... .......... ........MF ..........
```

```
                      1310       1320       1330       1340       1350       1360       1370       1380
                ....|....| ....|....| ....|....| ....|....| ....|....| ....|....| ....|....| ....|....|
REF.LFV.Q593B6  EHPWIQIDLQ RQVVITGIQT QGAMQLLKHL YTVEYFFTYS KDGQNWITFK GRHSETQMHF EGNSDGTTVK ENHIDPPIIA
REF.VFV.Q7SZN0  .......... .......... ..TV...Q.S ......V... E......... .......... .......... ..........
PTE.VFV.ID01    .......... .......... ..TV.....S ......V... E......... .......... .......... ..........
PTE.VFV.ID02    .......... .......... ..TV.....S ......V... E......... .......... .......... ..........
PTE.VFV.ID03    .......... .......... ..TV.....S ......V... E......... .......... .......... ..........
PTE.VFV.ID04    .......... .......... ..TV.....S ......V... E......... .......... .......... ..........
PTE.VFV.ID05    .......... .......... ..TV.....S ......V... E......... .......... .......... ..........
PAS.VFV.ID01    .......... .......... ..TV.....S ......V... E......... .......... .......... ..........
PAS.VFV.ID02    .......... .......... ..TV.....S ......V... E......... .......... .......... ..........
PAS.VFV.ID03    .......... .......... ..TV...... ......V... E......... .......... .......... ..........
PAS.VFV.ID04    .......... .......... ..TV...... ......V... E......... .......... .......... ..........
OMI.Q58L90      .......... .......... ..TV.....S ......V... .......... .......... .......... ..........
OSC.Q58L91      .......... .......... ..TV.....S ......V... .......... ....K..... .......... ..........
OSC.AAY47065    .......... .......... ..TVH....S ......V... .......... ....K..... .......... ..........

                      1390       1400       1410       1420       1430       1440       1450       1460
                ....|....| ....|....| ....|....| ....|....| ....|....| ....|....| ....|....| ....|....|
REF.LFV.Q593B6  RYIRLHPTKF HNRPTFRIEL LGCEVEGCSV PLGMESGAIK NSEITASSYK KTWWSSWEPS LARLNLKGRT NAWQPKVNNK
REF.VFV.Q7SZN0  .......... Y......... .......... .......... .......... .......... ......E.G. .....E....
PTE.VFV.ID01    .......... Y......... .......... .......... .......... .........F ......E.G. .....E....
PTE.VFV.ID02    .......... Y......... .......... .......... .......... .........F ......E.G. .....E....
PTE.VFV.ID03    .......... Y......... .......... .......... .......... .........F ......E.G. .....E....
PTE.VFV.ID04    .......... Y......... .......... .......... .......... .........F ......E.G. .....E....
PTE.VFV.ID05    .......... Y......... .......... .......... .......... .........F ......E.G. .....E....
PAS.VFV.ID01    .......... Y......... .......... .......... .......... .........F ......E.G. .....E....
PAS.VFV.ID02    .......... Y......... .......... .......... .......... .........F ......E.G. .....E....
PAS.VFV.ID03    .......... Y......... .......... .......... .......... .........F ......E.G. .....E....
PAS.VFV.ID04    .......... Y......... .......... .......... .......... .........F ......E.G. .....E....
OMI.Q58L90      .......... Y.T....... .......... .......... .......... .........F ......E.G. .....E....
OSC.Q58L91      .......... Y.T....... .......... .......... D......... .........F .......... ..........
OSC.AAY47065    .......... Y.T....... .......... .......... D......... .........F .......... ..........

                      1470       1480       1490       1500       1510       1520       1530       1540
                ....|....| ....|....| ....|....| ....|....| ....|....| ....|....| ....|....| ....|....|
REF.LFV.Q593B6  DQWLQIDLQH LTKITSIITQ GATSMTTSMY VKTFSIHYTD DNSTWKPYLD VRTSMEKVFT GNINSDGHVK HFFKPPILSR
REF.VFV.Q7SZN0  .......... .......... .......... .......... .......... .......... .......... ..........
PTE.VFV.ID01    .......... .......... .......... .......... .......... .......... .......... ..........
PTE.VFV.ID02    .......... .......... .......... .......... .......... .......... .......... ..........
PTE.VFV.ID03    .......... .......... .......... .......... .......... .......... .......... ..........
PTE.VFV.ID04    .......... .......... .......... .......... .......... .......... .......... ..........
PTE.VFV.ID05    .......... .......... .......... .......... .......... .......... .......... ..........
PAS.VFV.ID01    .......... .......... .......... .......... .......... .H........ .......... ..........
PAS.VFV.ID02    .......... .......... .......... .......... .......... .H........ .......... ..........
PAS.VFV.ID03    .......... .......... .......... .......... .......... .......... .......... ..........
PAS.VFV.ID04    .......... .......... .......... .......... .......... .......... .......... ..........
OMI.Q58L90      .......... .......... .......A.. .......... .......... .......... .......... ..........
OSC.Q58L91      .......... .......... .......... .......... .......... .......... .......... ...N......
OSC.AAY47065    .......... .......... .......... .......... .......... .......... .......... ...N......

                      1550       1560
                ....|....| ....|....| ....|
REF.LFV.Q593B6  FIRIIPKTWN QYIALRIELF GCEVF Liver
REF.VFV.Q7SZN0  .......... .......... ..... Venom
PTE.VFV.ID01    .......... .......... ..... 99.23%
PTE.VFV.ID02    .......... .......... ..... 96.93%
PTE.VFV.ID03    .......... .......... ..... 96.17%
PTE.VFV.ID04    .......... .......... ..... 98.46%
PTE.VFV.ID05    .......... .......... ..... 98.46%
PAS.VFV.ID01    .......... .......... ..... 98.18%
PAS.VFV.ID02    .......... .......... ..... 95.90%
PAS.VFV.ID03    .......... .......... ..... 98.11%
PAS.VFV.ID04    .......... .......... ..... 95.83%
OMI.Q58L90      .......... .......... ..... 97.00%
OSC.Q58L91      .......... .......... ..... 96.15%
OSC.AAY47065    .......... .......... ..... 96.08%
```

Fig. S3. Venom factor V sequence alignment. Our de novo transcripts were aligned with reference sequences from Australian brown snakes (*Pseudonaja* species) and taipans (*Oxyuranus* species). In the sequence alignment, dots (.) indicate identical residues with the reference (UniProt ID: Q593B6), while dashes (-) represent gaps.

The percentages reflect the similarity between our transcripts and the *P. textilis* venom isoform (UniProt ID: Q7SZN0), which was used as the reference.

```
                          10         20         30         40         50         60
                  ....|....| ....|....| ....|....| ....|....| ....|....| ....|....|
REF.PLA2.Q9W7J4   SLDDFSNLIT CANRGSRSWL DYAHYGCFCG SGGSGTPVDD LDRCCQVHDN CFGDAEKLPA
PAF.UNW14929      ...N...... .......N.W .......... .........E ......A... ..........
PAF.UNW14930      ...N...... .......N.W .......... .........E ......A... ..........
PNU.UNW14933      ...N..S... .......N.W .......... .........E ......E... ..........
PAS.PLA2.ID05     ...N...... .......N.W .......... .........E .......... ..........
PTE.PLA2.ID07     ...N...... .......N.W .......... .........E ......A... ..........
PTE.PLA2.ID08     .......... .......... .......... .......... .......... ..........
PMO.PLA2.ID01     D.I....... .......... .......... .......... .......... .Y........
PTE.PLA2.ID09     .......... .......... .......... .......... .......... ..........
REF.PLA2.Q9W7J3   ..VE.RI..K ...HN..NV. ...D...Y.. K........E ......A..Y .YD.......
PAS.PLA2.ID03     ..VE.RI..K ...HN..NV. ...D...Y.. K........E ......A..Y .YD.......
PAS.PLA2.ID04     ..VE.RI..K ...HN..NV. ...D...Y.. K..R.....E ......A..Y .YD.......
PTE.PLA2.ID06     ..VE.RI..K ...HN..NV. ...D...Y.. K........E ......A..Y .YD.......
PTE.PLA2.ID05     ..VE.RI..K ...HN..NV. ...D...Y.. K........E ......A..Y .YD.......
PTE.PLA2.ID04     ..VE.RI..K ...HN..NV. ...D...Y.. K........E ......A..Y .YD.......
PTE.PLA2.ID03     ..VE.RI..K ...HN..NV. ...D...Y.. K........E ......A..Y .YD.......
PAS.PLA2.ID02     ..VE.RI..K ...HN..NV. ...D...Y.. K..R.....E ......A..Y .YD.......
PAS.PLA2.ID01     ..LQ.RI..K ...HN..DV. ...N...Y.. K........E ......A..Y .YD.......
PMO.PLA2.ID02     ..VE.RS..K ...HN..NVF ..VD...Y.. K........E ......A..Y .YD.......
OSC.Q4VRI5        ..LN.A...E ...H.T..A. A..D...Y.. K..R...L.. .....H...D .Y.E......
OSC.AAZ22633      ..LN.A...E ...H.T..A. A..D...Y.. K..R...L.. .........D .Y.E......
OMI.AAZ22639      ..L..A...E ...H.T..A. A.V....Y.. K..R.....E .........D .Y.E......
                          70         80         90        100        110        120
                  ....|....| ....|....| ....|....| ....|....| ....|....| ....|....|
REF.PLA2.Q9W7J4   CNYLFSGPYW NPYSYKCNEG EITCTDDNDE CAAFICNCDR TAAICFAGAT YNDENFMVTI
PAF.UNW14929      ...R...... .......... .......... .K........ .......... .......I..
PAF.UNW14930      ...R...... .......... .......... .K........ .........P .......I..
PNU.UNW14933      ...R...... .....E.... .......K.. .K........ .......... ..........
PAS.PLA2.ID05     .......... .......... .......K.. .K........ .......... .......I.T
PTE.PLA2.ID07     ...R...... .......... .V........ .K........ .........P .......I.T
PTE.PLA2.ID08     .......... .......... .......... .......... .......... ..........
PMO.PLA2.ID01     ...Q..S... .......... .......K.. .K........ .......R.. .K.....IST
PTE.PLA2.ID09     .......... .......... .V........ .......... .......... ..........
REF.PLA2.Q9W7J3   ...R...... .......... .V........ .K........ .........P .......I..
PAS.PLA2.ID03     ...R...... .......... .V.....K.. .K........ .........P .......I.T
PAS.PLA2.ID04     ...R...... .......... .V.....K.. .K........ .........P .......I.T
PTE.PLA2.ID06     ...R...... .......... .V........ .K........ .........P .......I.T
PTE.PLA2.ID05     ...R...... .......... .V........ .K........ .........P .......I.T
PTE.PLA2.ID04     .......... .......... .V........ .K........ .........P .......I..
PTE.PLA2.ID03     .......... .......... .......... .......... .......... ..........
PAS.PLA2.ID02     ...R...A.Y .S........ K......K.. .K........ .........P .......I.T
PAS.PLA2.ID01     ...R...A.Y .S........ .......K.. .K........ .........P .......I.T
PMO.PLA2.ID02     ...W...A.Y .S........ .......... .Q........ .......R.P .DKD.WNINT
OSC.Q4VRI5        ....M.S..F .S........ KV........ .K........ .......... .......ISK
OSC.AAZ22633      ....M.S..F .S........ KV........ .K........ .......... .......ISK
OMI.AAZ22639      ....M.S..F .S........ KV........ .K........ .......... .......ISK

                  ....|..
REF.PLA2.Q9W7J4   KKKNICQ Phospholipase A2 1
PAF.UNW14929      ....... 93.70%
PAF.UNW14930      ....... 92.91%
PNU.UNW14933      ....... 92.13%
PAS.PLA2.ID05     ....... 93.70%
PTE.PLA2.ID07     ....... 91.34%
PTE.PLA2.ID08     ....... 100.00%
PMO.PLA2.ID01     ..N.... 89.76%
PTE.PLA2.ID09     ....... 99.21%
REF.PLA2.Q9W7J3   ....... Phospholipase A2 2
PAS.PLA2.ID03     ....... 98.43%
PAS.PLA2.ID04     ....... 97.64%
PTE.PLA2.ID06     ....... 99.21%
PTE.PLA2.ID05     ....... 99.21%
PTE.PLA2.ID04     ....... 99.21%
PTE.PLA2.ID03     ....... 96.06%
PAS.PLA2.ID02     ....... 93.70%
PAS.PLA2.ID01     ....... 92.13%
PMO.PLA2.ID02     ETD--.- 81.89%
OSC.Q4VRI5        .RND... 76.38%
OSC.AAZ22633      .RND... 77.17%
OMI.AAZ22639      .RND... 77.17%
```

Fig. S4. Group I phospholipase A_2 sequence alignment. Our *de novo* transcripts, which phylogenetically clustered with known procoagulant phospholipase A_2 (PLA_2) isoforms, were aligned with reference sequences from Australian brown snakes (*Pseudonaja* species) and taipans (*Oxyuranus* species). In the sequence alignment, dots (.) indicate identical residues with the reference (UniProt IDs: Q9W7J4), while dashes (-) represent gaps. The percentages indicate the similarity to either of the *P. textilis* procoagulant PLA_2 isoforms (UniProt IDs: Q9W7J4 and Q9W7J3).

```
                             10         20         30         40         50         60
                    ....|....| ....|....| ....|....| ....|....| ....|....| ....|....|
REF.VTK2.Q90WA0     KDRPELCELP PDTGPCRVRF PSFYYNPDEQ KCLEFIYGGC EGNANNFITK EECESTCAA- Textilinin 2
PTE.KTSPI.ID08      ....DF.... A......... .........K .......... G....K.N.L .........- 86.44%
PTE.KTSPI.ID05      .......... .......... .........K .......... .......... ........RK 95.00%
PTE.KTSPI.ID06      .......... .......... .......... .......... .......... .........- 100.00%
PTE.KTSPI.ID07      ....K..... .......... .......... .......... .......... .........- 98.31
REF.VTK1.Q90WA1     ....DF.... A......... .........K .......... .......... .........- Textilinin 1
OSC.Q6ITB7          ....DF.... A.......G. .........K .......... ..S....... .........- 96.61%
PAU.Q6ITB9          ....DF.... A.......G. .........K .......... Q......... .........- 96.61%
OMI.Q6ITB5          ....D..... A.......G. .........K .......... .......... .........- 96.61%
OMI.Q6ITB4          ....DF.... A.......G. .........K .......... .......... .........- 98.31%
OSC.Q6ITB6          ....DF.... A.......G. .........K .......... .......... .........- 98.31%
PNU.UNW14913        N...DF.... A.......K. .........K .......... .......... .........- 96.61%
PAS.KTSPI.ID03      ....DF.... A......... .........K .......... .......... ........GK 96.67%
PTE.KTSPI.ID11      ....DF.... A......... ......L..K .......... .......... .........- 98.31%
PTE.KTSPI.ID09      ....DF.... A......... .........K .......... .......... .........- 100.00%
PTE.KTSPI.ID10      ....DF.... A......... .........K .......... .......... .........- 100.00%
PAS.KTSPI.ID04      ....DF.... A......... .........K .......... .......... .........- 100.00%
PMO.KTSPI.ID07      ....DF.... A......... .........K .......... .......... .........- 100.00%
REF.VTK6.Q90W96     ....KF.... A.I...DDFT GA.H.S.R.H E.I....... K......N.Q .........- Textilinin 6
PTE.KTSPI.ID12      ....KF.... A.I...DDFT GA.H.S.R.H E.I....... K......N.Q .....A...- 98.31%
PTE.KTSPI.ID13      ....NF.... A.M...DDFT GA.H.S.R.H E.I....... G....K.N.L .........- 91.53%
PTE.KTSPI.ID14      ....DF.... A.M...DDFT GA.H.S.R.H E.I....... G....K.N.L .........- 91.53%
REF.VTK5.Q90W97     ....KF...L ....S.EDFT GA.H.STRDR E.I....... G......... .........- Textilinin 5
REF.VTK7.Q90W96     ....KF...L ....S.EDFT GA.H.STRDR E.I....... G......K.L .........- Textilinin 7
PAS.KTSPI.ID01      ....NF.... A.M...DDFT GA.H.STRDR E.I....... G....K.N.L .....A..P- 83.05%
PMO.KTSPI.ID08      ....NF.... A.M...DDFT GA.H.STRDR E.I....... G....K.N.L .....A..P- 83.05%
PAS.KTSPI.ID02      ....NF.... A.M...DDFT GA.H.STRDR E.I....... G....K.N.Q .....A..P- 81.36%
PTE.KTSPI.ID15      ....KF...L ......DDFT GA.H.STRDR E.I....... K......N.Q .....A...- 89.83%
PTE.KTSPI.ID16      ....KF...L ......DDFT GA.H.STRDR E.I....... G....K.N.L ........P- 91.53%
PTE.KTSPI.ID17      ....KF...L ......DDFT GA.H.STRDR E.I....... G....K.N.L ........RK 90.00%
REF.VTK4.Q90W98     ..H.KF.... A...S.KGNV .R....A.HH Q..K...... G......K.I ...K.....- Textilinin 4
PAS.KTSPI.ID06      ....KF.... A...S.KGNV .R....A.HH Q..K...... G......K.I ...K....GK 95.00%
PAS.KTSPI.ID05      .....F.... A...S.KGNV .R....A.HH Q..K...... G......K.I ...K....GK 93.33%
PTE.KTSPI.ID02      .....F.... A...S.KGNV .R....A.HH Q..K...... G......K.I ...K.....- 96.61%
PTE.KTSPI.ID01      ..H.KF.... A...S.KGNV .R....A.HH Q..K...... G......K.I ...K.....- 100.00%
PAS.KTSPI.ID07      ..H.KF.... A...S.KGNV .R....A.HH Q..K...... G......K.I ...K.....- 100.00%
PAS.KTSPI.ID08      ....KF.... A...S.KGNV .R....A.HH Q..K...... G......K.I ...K.....- 98.31%
REF.VTK3.Q90W99     ....NF.K.. AE..R.NAKI .R.....RQH Q.I..L.... G......K.I K........- Textilinin 3
PTE.KTSPI.ID04      ....NF.K.. AE..R.NAKI .R.....RQH Q.I..L.... G....K.N.L .........- 84.75%
PMO.KTSPI.ID01      ....NF.... AE..L.NGHI TR.....RQH Q.IK.L.... G......K.I G..K...I.- 93.22%
PTE.KTSPI.ID03      ....NF.K.. AE..R.NAKI .R.....RQH Q.I..L.... G......K.I K........- 100.00%
PAS.KTSPI.ID09      ....NF.K.. AE..R.NAKI .R.....RQH Q.I..L.... G......K.I K..K...I.- 96.61%
PMO.KTSPI.ID02      ..P.NF.K.. AE..R.NGHI TR.....RQH Q.I....... G......K.I K..K...I.- 88.14%
PAS.KTSPI.ID11      ..P.NF.K.. AE..R.NGKI TR.....RQH Q.I..L.... G......K.I K..K...I.- 91.53%
PMO.KTSPI.ID04      ..P.NF.K.. AE..R.NGKI TR.....RQH Q.I..L.... G......K.I K..K...I.- 91.53%
PMO.KTSPI.ID03      ..P.NF.K.. AE..R.NGKI TR.....RQH Q.I..L.... G......K.I ...K...I.- 89.83%
PMO.KTSPI.ID05      ..P.NF.K.. AE..R.NGKI TR.....RQH Q.I..L.... G......K.I K..K...IGK 88.33%
PAS.KTSPI.ID10      ..P.NF.K.. AE..R.NGKI TR.....RQH Q.I..L.... G......K.I ...K....GK 88.33%
PMO.KTSPI.ID06      ..P.NF.K.. AE..R.NGKI TR.....RQH Q.I..L.... G......K.I ...K...IGK 86.67%
```

Plasmin-inhibiting KTSPI

Non-plasmin-inhibiting KTSPI

Fig. S5. Kunitz type serine protease inhibitor sequence alignment. Our *de novo* transcripts were aligned with both plasmin-inhibiting Kunitz type serine protease inhibitor (KTSPI) isoforms and non-plasmin-inhibiting KTSPI isoforms. In the sequence alignment, dots (.) indicate identical residues with the reference (UniProt IDs: Q90WA0), while dashes (-) represent gaps. The plasmin-inhibiting isoforms (in the literature commonly referred to as “textilinin-1” or “textilinin-2”) are characterized by two key amino acids for plasmin inhibition and specificity, namely arginine, 17R; and valine, 18V (both highlighted in bold). The other *Pseudonaja textilis* KTSPI isoforms have shown to lack any plasmin-inhibition. The percentages indicate similarity to one

of the seven previously identified *Pseudonaja textilis* KTSPI genes (UniProt IDs: Q90WA1, Q90WA0, Q90W99, Q90W98, Q90W97, Q90W96 & B5L5Q1).

Factor X

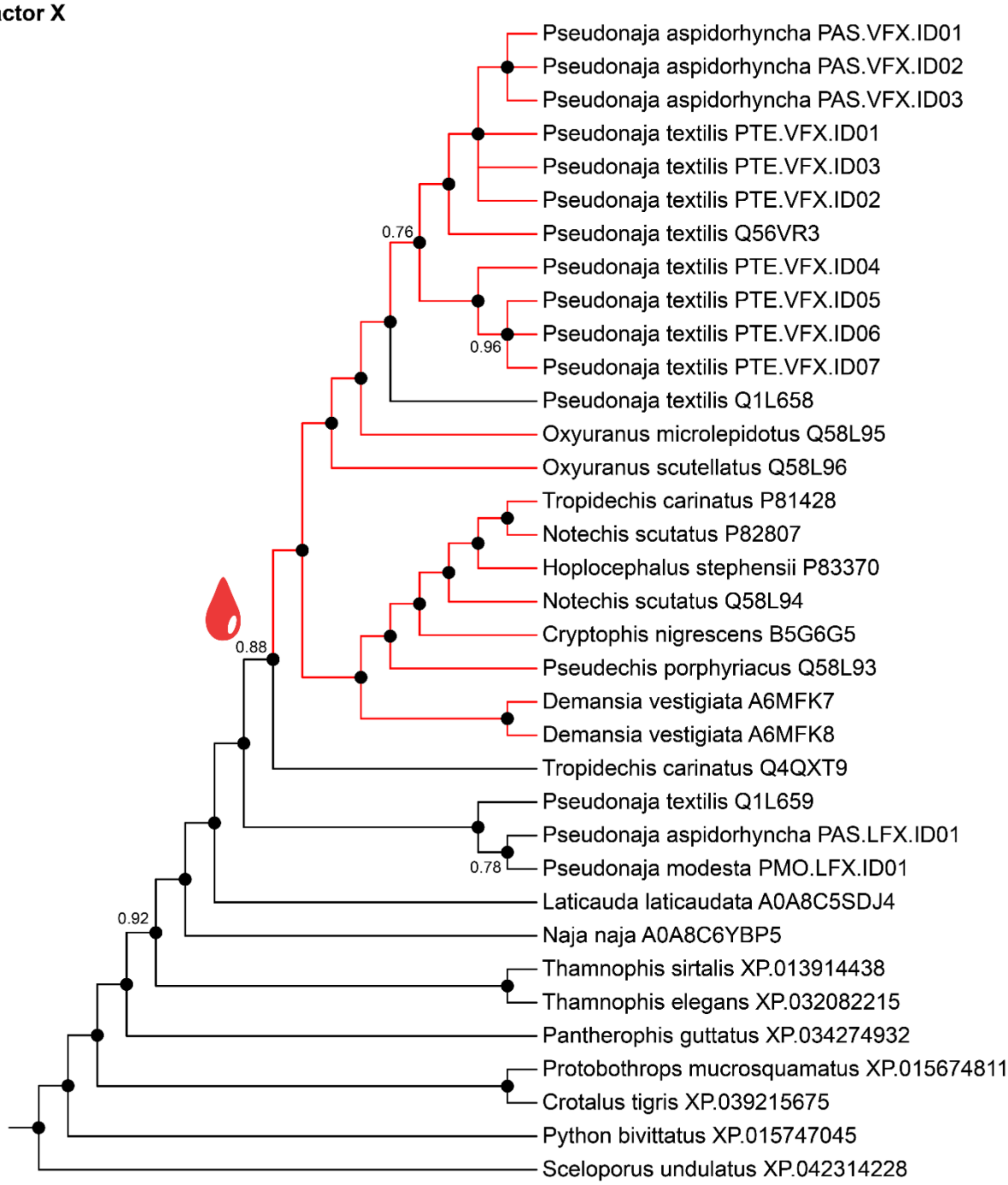

Fig. S6. Bayesian inference-derived amino acid phylogeny of factor X sequences. Only Bayesian posterior probabilities below 1.0 are displayed. Black branches indicate liver-specific genes and red branches indicate venom-specific genes. The tree was rooted using *Sceloporus undulatus* (eastern fence lizard) as a representative outgroup.

Factor X

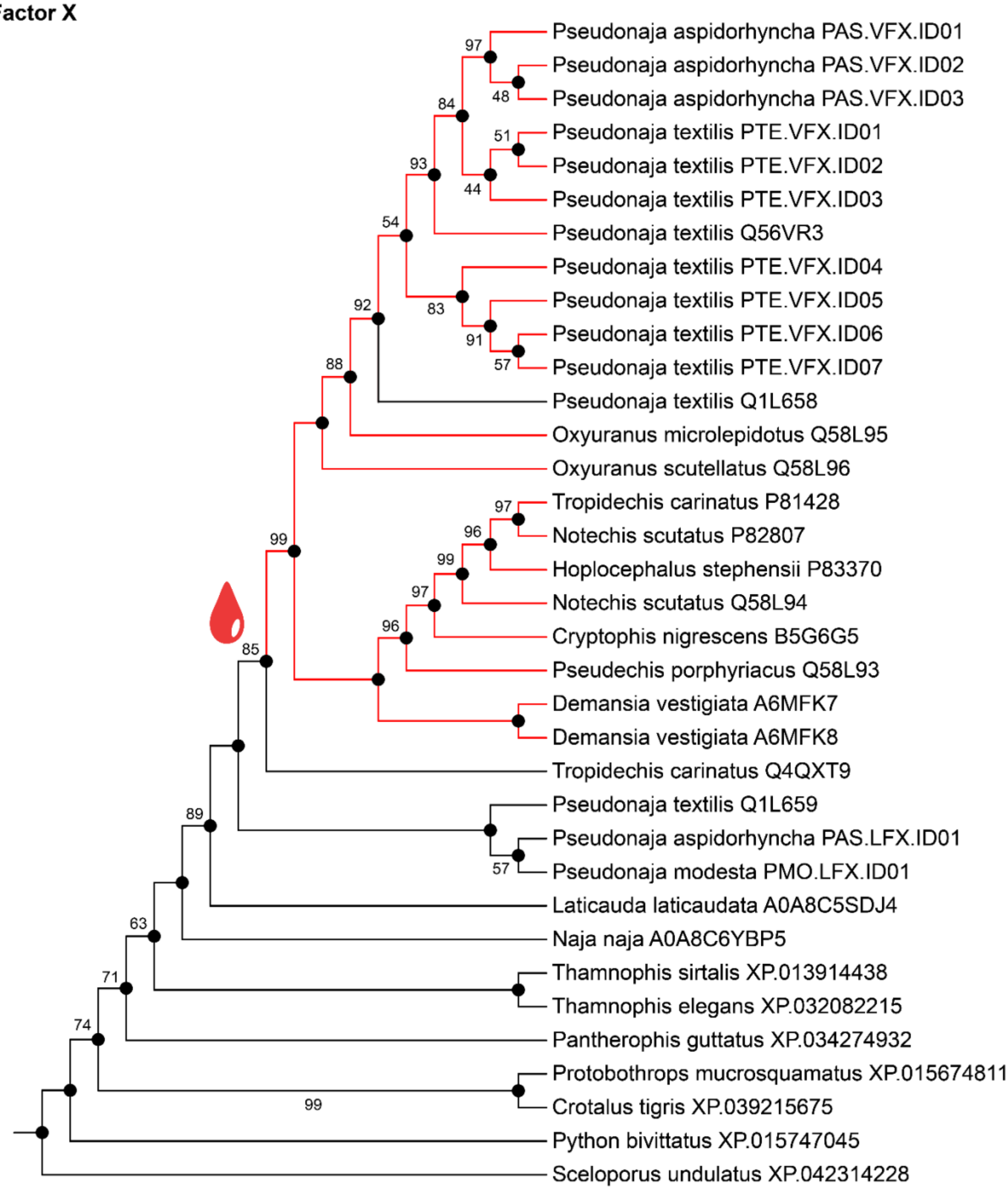

Fig. S7. Maximum-likelihood estimation-derived amino acid phylogeny of factor X sequences. Only bootstrap values below 100 are displayed. Black branches indicate liver-specific genes and red branches indicate venom-specific genes. The tree was rooted using *Sceloporus undulatus* (eastern fence lizard) as a representative outgroup.

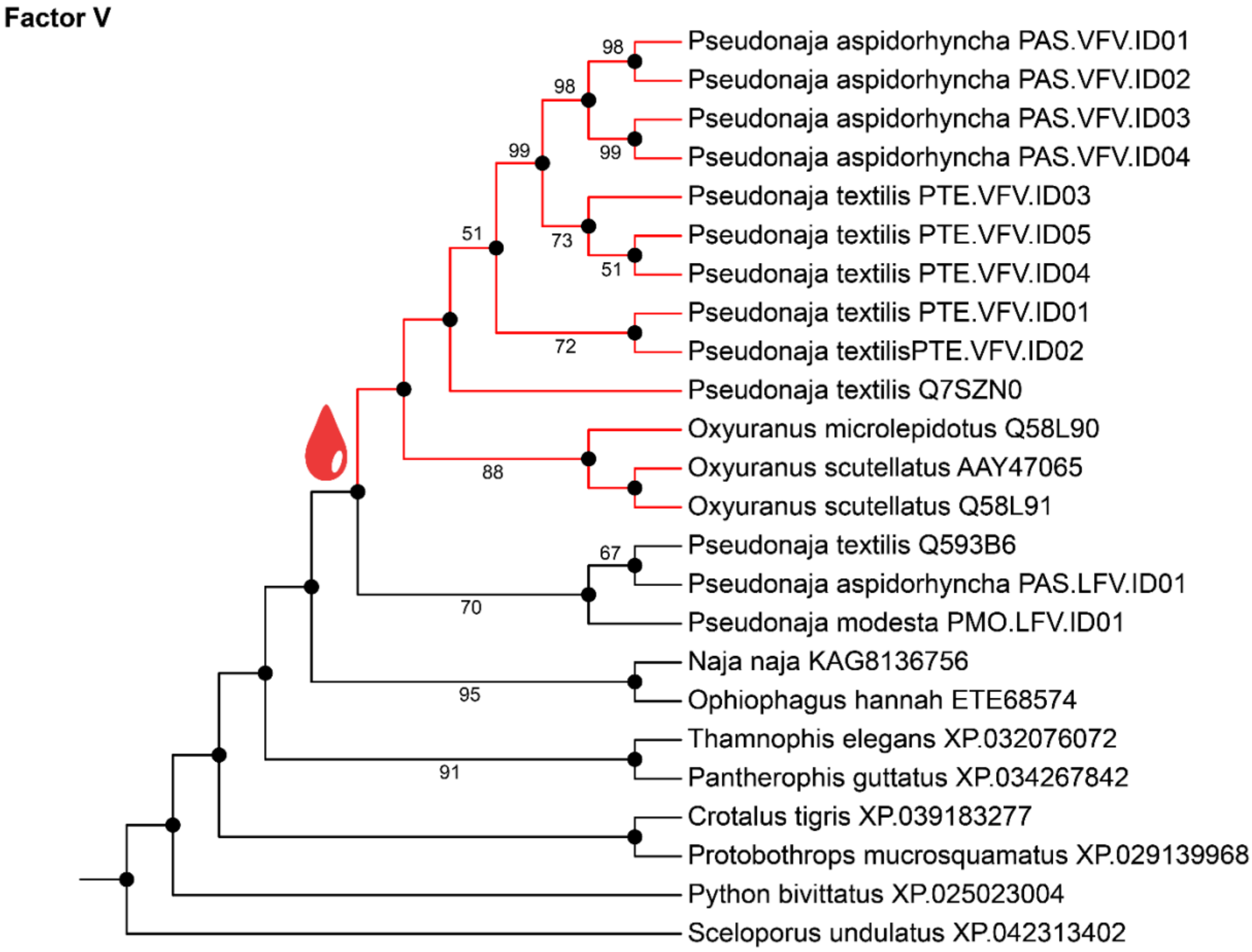

Fig. S8. Bayesian inference-derived amino acid phylogeny of factor V sequences. Only Bayesian posterior probabilities below 1.0 are displayed. Black branches indicate liver-specific genes and red branches indicate venom-specific genes. The tree was rooted using *Sceloporus undulatus* (eastern fence lizard) as a representative outgroup.

Factor V

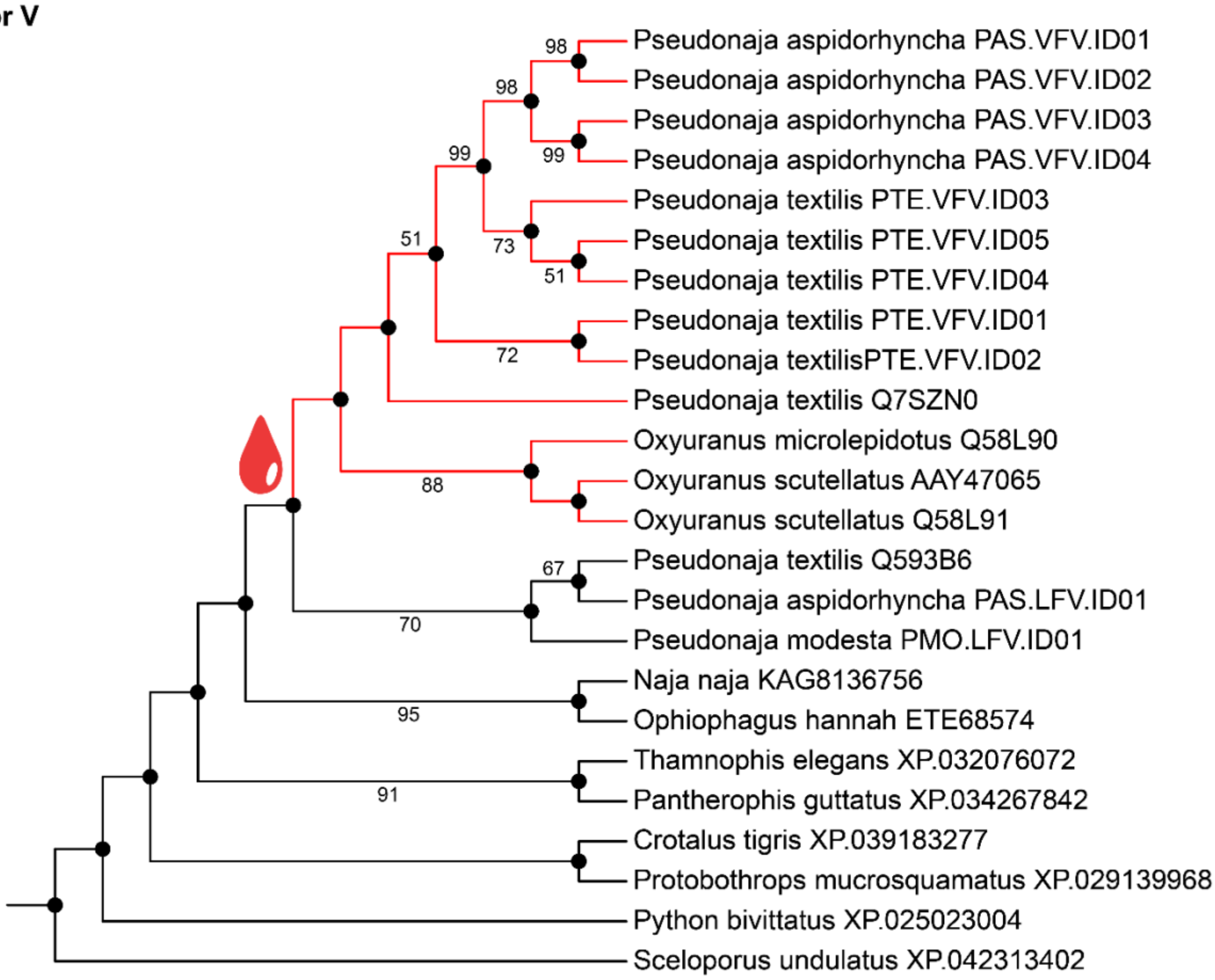

Fig. S9. Maximum-likelihood estimation-derived amino acid phylogeny of factor V sequences. Only Bootstrap values below 100 are displayed. Black branches indicate liver-specific genes and red branches indicate venom-specific genes. The tree was rooted using *Sceloporus undulatus* (eastern fence lizard) as a representative outgroup.

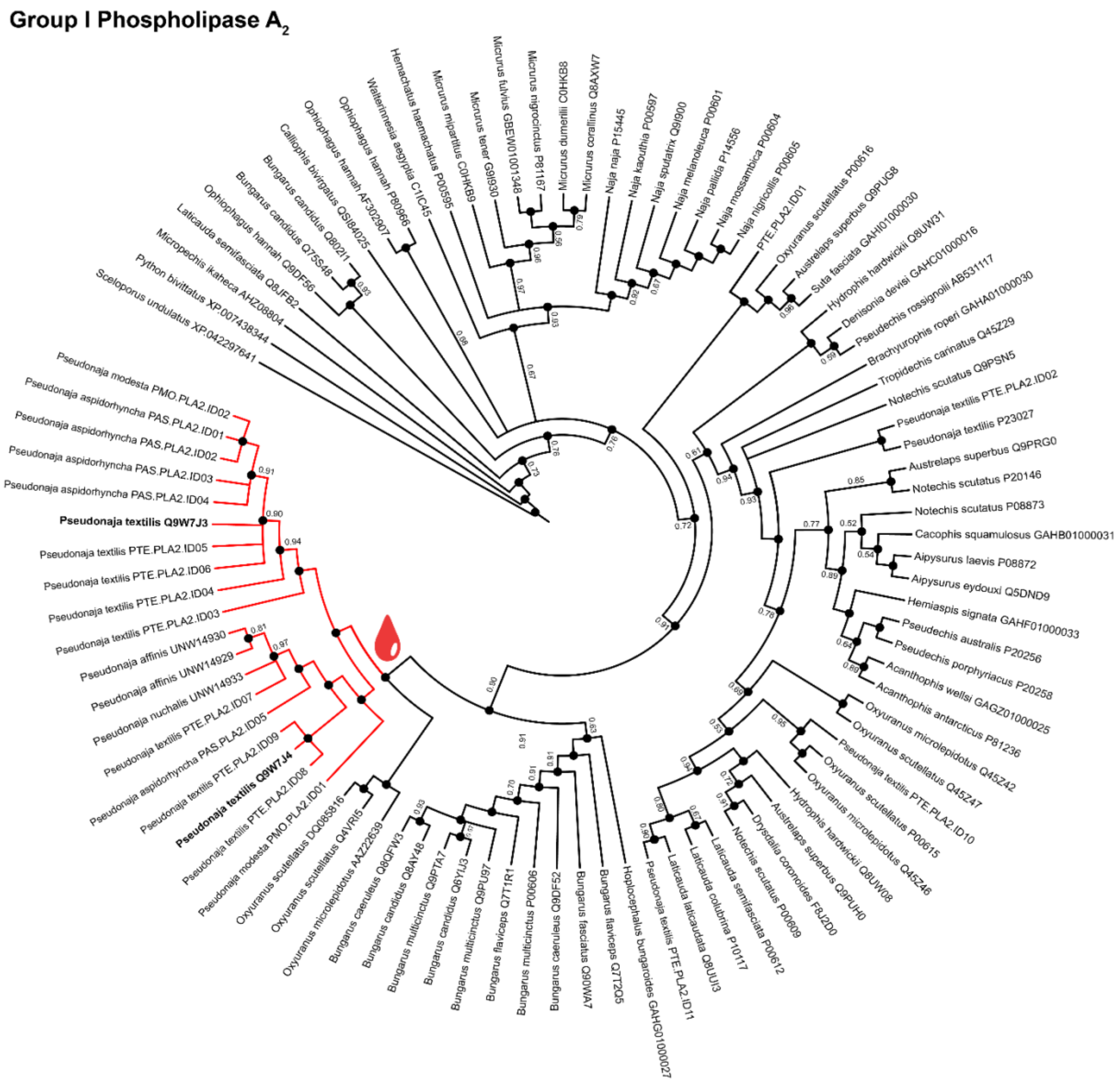

Fig. S10. Bayesian inference-derived phylogeny of group I phospholipase A_2 sequences from elapid venoms. The majority of our group I phospholipase A_2 (PLA_2) transcripts clusters with procoagulant isoforms (Q9W7J3 and Q9W7J4). Red branches indicate the inferred procoagulant PLA_2 cluster. Only Bayesian posterior probabilities below 1.0 are displayed. The tree was rooted using *Sceloporus undulatus* (eastern fence lizard) as a representative outgroup.

Group I Phospholipase A_2

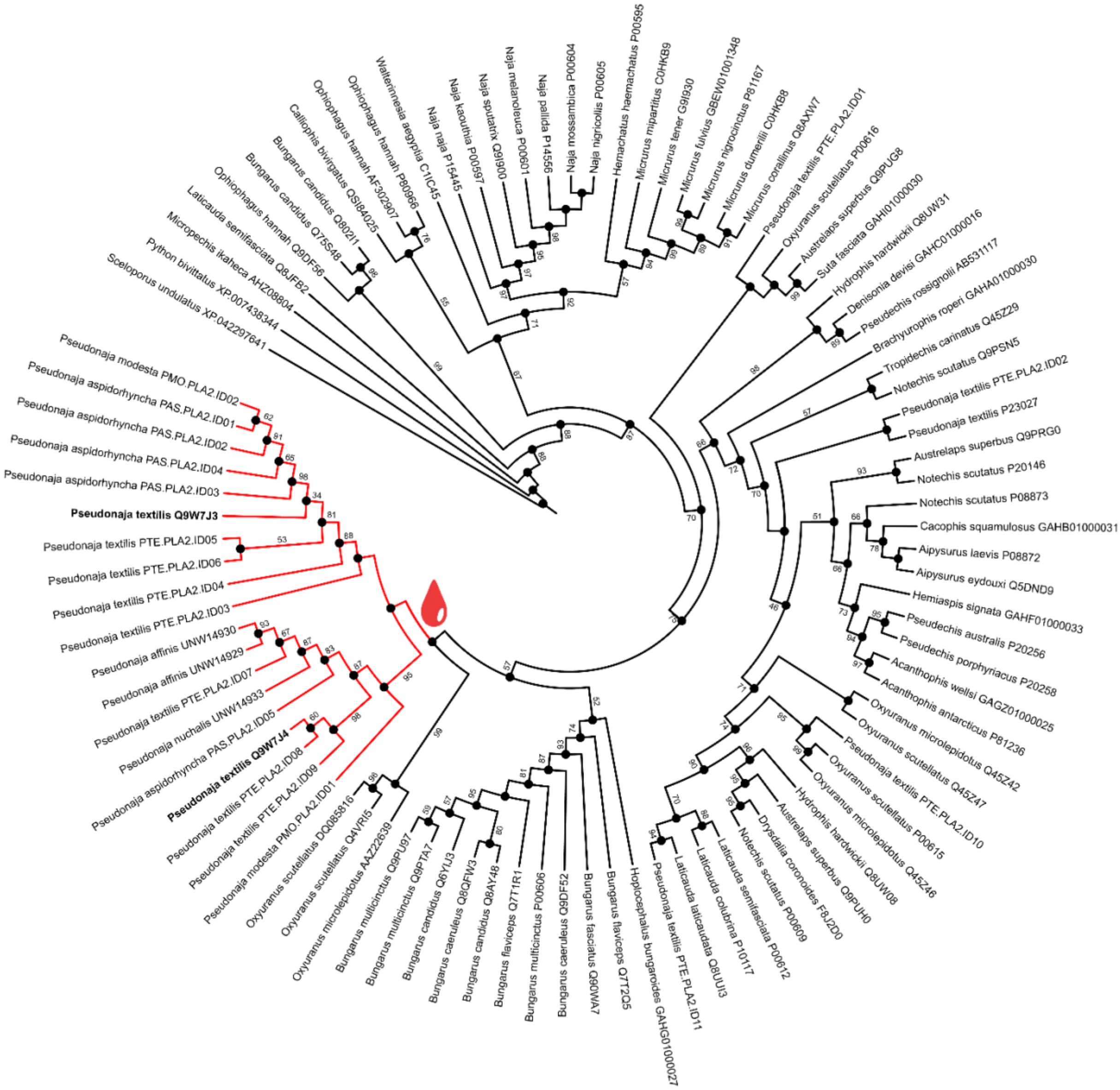

Fig. S11. Maximum-likelihood estimation-derived phylogeny of group I phospholipase A_2 sequences from elapid venoms. The majority of our group I phospholipase A_2 (PLA_2) transcripts clusters with procoagulant isoforms (Q9W7J3 and Q9W7J4). Red branches indicate the inferred procoagulant PLA_2 cluster. Only Bootstrap values below 100 are displayed. The tree was rooted using *Sceloporus undulatus* (eastern fence lizard) as a representative outgroup.

Kunitz-type serine protease inhibitors

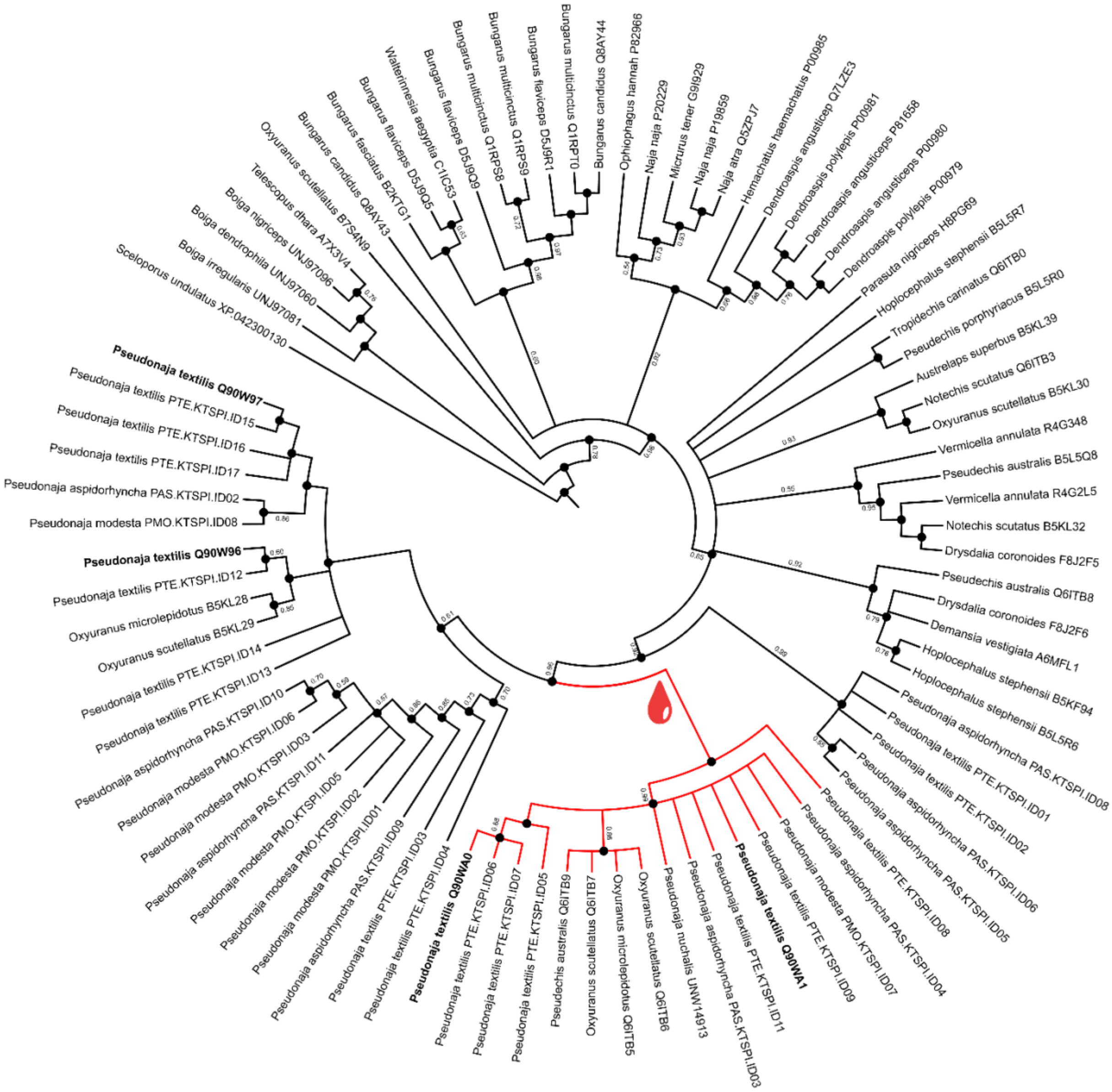

Fig. S12. Bayesian inference-derived phylogeny of Kunitz-type serine protease inhibitor sequences from elapid venoms. Several of our Kunitz-type serine protease inhibitor (KTSPI) transcripts cluster with plasmin-inhibiting isoforms (Q90WA1 and Q90WA0). Only this cluster contains the key amino acids for plasmin inhibition and specificity (i.e. arginine, 17R; and valine, 18V). Only Bayesian posterior probabilities below 1.0 are displayed. Red branches indicate plasmin inhibiting KTSPI sequences (with key amino acids) and black branches indicate non-plasmin inhibiting KTSPI

sequences (lack of key amino acids). The tree was rooted using *Sceloporus undulatus* (eastern fence lizard) as a representative outgroup.

Kunitz-type serine protease inhibitors

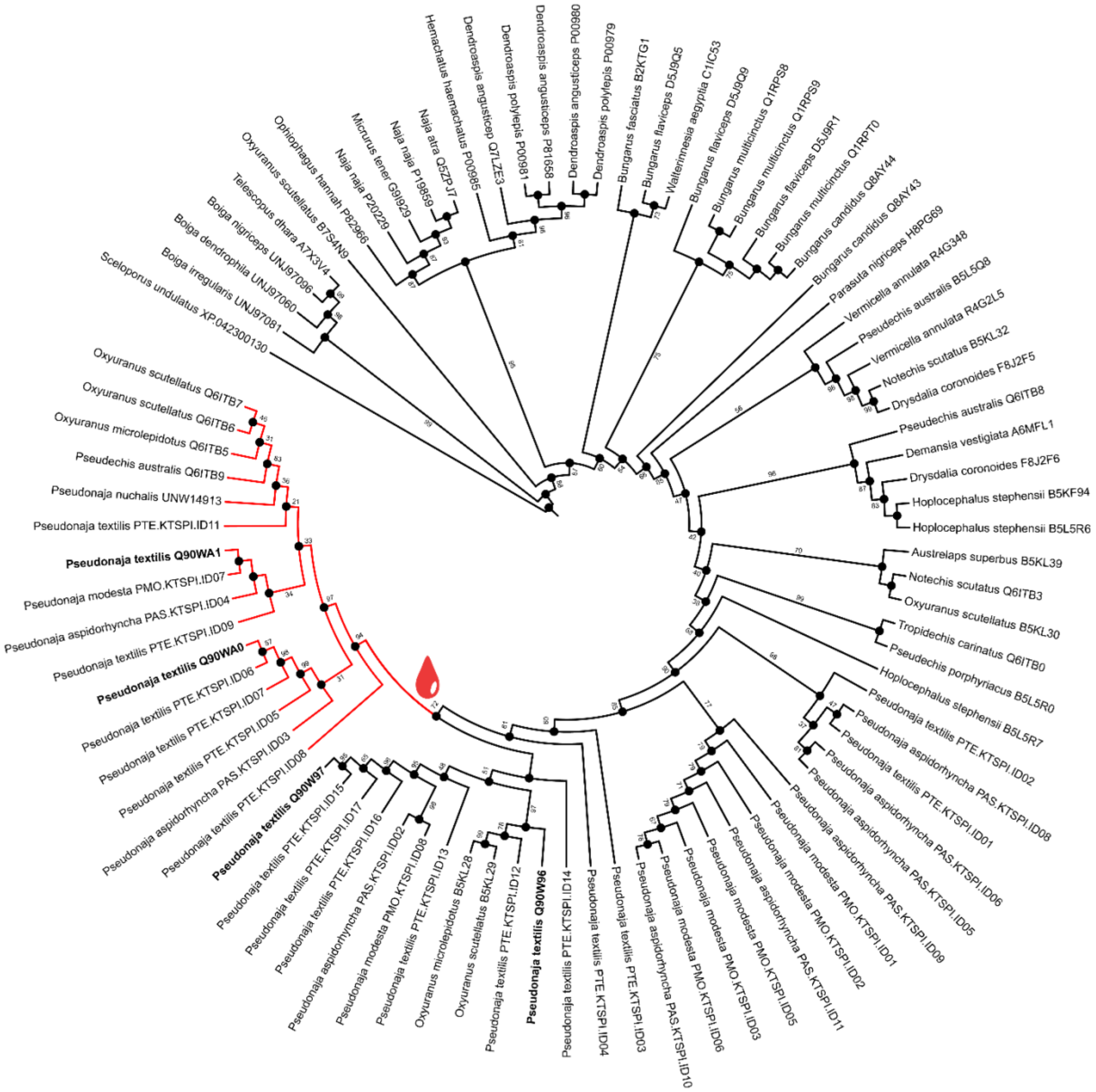

Fig. S13. Maximum-likelihood estimation-derived phylogeny of Kunitz-type serine protease inhibitor sequences from elapid venoms. Several of our Kunitz-type serine protease inhibitor (KTSPI) transcripts cluster with plasmin-inhibiting isoforms (Q90WA1 and Q90WA0). Only this cluster contains the key amino acids for plasmin inhibition and specificity (i.e. arginine, 17R; and valine, 18V). Only Bayesian posterior probabilities below 1.0 are displayed. Red branches indicate plasmin inhibiting KTSPI sequences (with key amino acids) and black branches indicate non-

plasmin inhibiting KTSPI sequences (lack of key amino acids). The tree was rooted using *Sceloporus undulatus* (eastern fence lizard) as a representative outgroup.

Short-chain 3FTx

```
AAD40967 PT-SNTX1        LTCYKGYHDT VVCKPHETIC YEYFIPATHG NAILARGCGT SCPGGIRPVC CRTDLCNK
PMO.SC-3FTX.ID32         LTCYKGYHDT VVCKPHETIC YEYFIPATHG NAILARGCGT SCPGGIRPVC CRTDLCNK
PTE.SC-3FTX.ID21         LTCYKGYHDT VVCKPHETIC YEYFIPATHG NAILARGCGT SCPGGIRPVC CRTDLCNK

AAD40968 PT-SNTX2        LTCYKGYHDT VVCKPHETIC YRYLIPATHG NAIPARGCGT SCPGGNHPVC CSTDLCNK
PTE.SC-3FTX.ID17         LTCYKGYHDT VVCKPHETIC YRYLIPATHG NAIPARGCGT SCPGGNHPVC CSTDLCNK

AAD40969 PT-SNTX3        LTCYKGYHDT VVCKPHETIC YRYLVPATHG NAIPARGCGT SCPGGNHPVC CSTDLCNK
PAS.SC-3FTX.ID18         LTCYKGYHDT VVCKPHETIC YRYLVPATHG NAIPARGCGT SCPGGNHPVC CSTDLCNK
PTE.SC-3FTX.ID19         LTCYKGYHDT VVCKPHETIC YRYLVPATHG NAIPARGCGT SCPGGNHPVC CSTDLCNK

AAD40973 PT-SNTX7        LTCYKRYFDT VVCKPQETIC YRYIIPATHG NAITYRGCST SCPSGIRLVC CSTDLCNK
PTE.SC-3FTX.ID09         LTCYKRYFDT VVCKPQETIC YRYIIPATHG NAITYRGCST SCPSGIRLVC CSTDLCNK
```

Long-chain-like 3FTx

```
A8HDK6 PT-LNTX-1         LICYLDFSVP HTCAPGEKLC YTRTWNDGRG TRIERGCAAT CPIPKKPEIH VTCCSTDRCN
PAS.LCL-3FTX.ID01        LICYLDFSVP HTCAPGEKLC YTRTWNDGRG TRIERGCAAT CPIPKKPEIH VTCCSTDRCN
PTE.LCL-3FTX.ID01        LICYLDFSVP HTCAPGEKLC YTRTWNDGRG TRIERGCAAT CPIPKKPEIH VTCCSTDRCN

A8HDK6 PT-LNTX-1         PHPKQKPH
PAS.LCL-3FTX.ID01        PHPKQKPH
PTE.LCL-3FTX.ID01        PHPKQKPH
```

Long-chain 3FTx

```
AAK15774 PT-PTb          RTCFITPDVK SKPCPPGQEV CYTKTWCDGF CGIRGKRVDL GCAATCPTPK KTGIDIICCS
PMO.LC-3FTX.ID13         RTCFITPDVK SKPCPPGQEV CYTKTWCDGF CGIRGKRVDL GCAATCPTPK KTGIDIICCS
PAS.LC-3FTX.ID10         RTCFITPDVK SKPCPPGQEV CYTKTWCDGF CGIRGKRVDL GCAATCPTPK KTGIDIICCS
PTE.LC-3FTX.ID07         RTCFITPDVK SKPCPPGQEV CYTKTWCDGF CGIRGKRVDL GCAATCPTPK KTGIDIICCS

AAK15774 PT-PTb          TDDCNTFPLR PRGRLSSIKD
PMO.LC-3FTX.ID13         TDDCNTFPLR PRGRLSSIKD
PAS.LC-3FTX.ID10         TDDCNTFPLR PRGRLSSIKD
PTE.LC-3FTX.ID07         TDDCNTFPLR PRGRLSSIKD
```

Fig. S14. Sequence alignment of three-finger toxins with identical, functionally confirmed neurotoxins. Our *de novo* three-finger toxin (3FTx) transcripts were aligned to identical, functionally confirmed neurotoxin sequences. The neurotoxin references are highlighted in bold. The following studies described the neurotoxins: short-chain 3FTx references: AAD40967, AAD40968, AAD40969, and AAD40973; long-chain-like 3FTx reference: A8HDK6; long-chain 3FTx reference: AAK15774. Note: only the *de novo* 3FTx transcripts that matched 100% identity are displayed above.

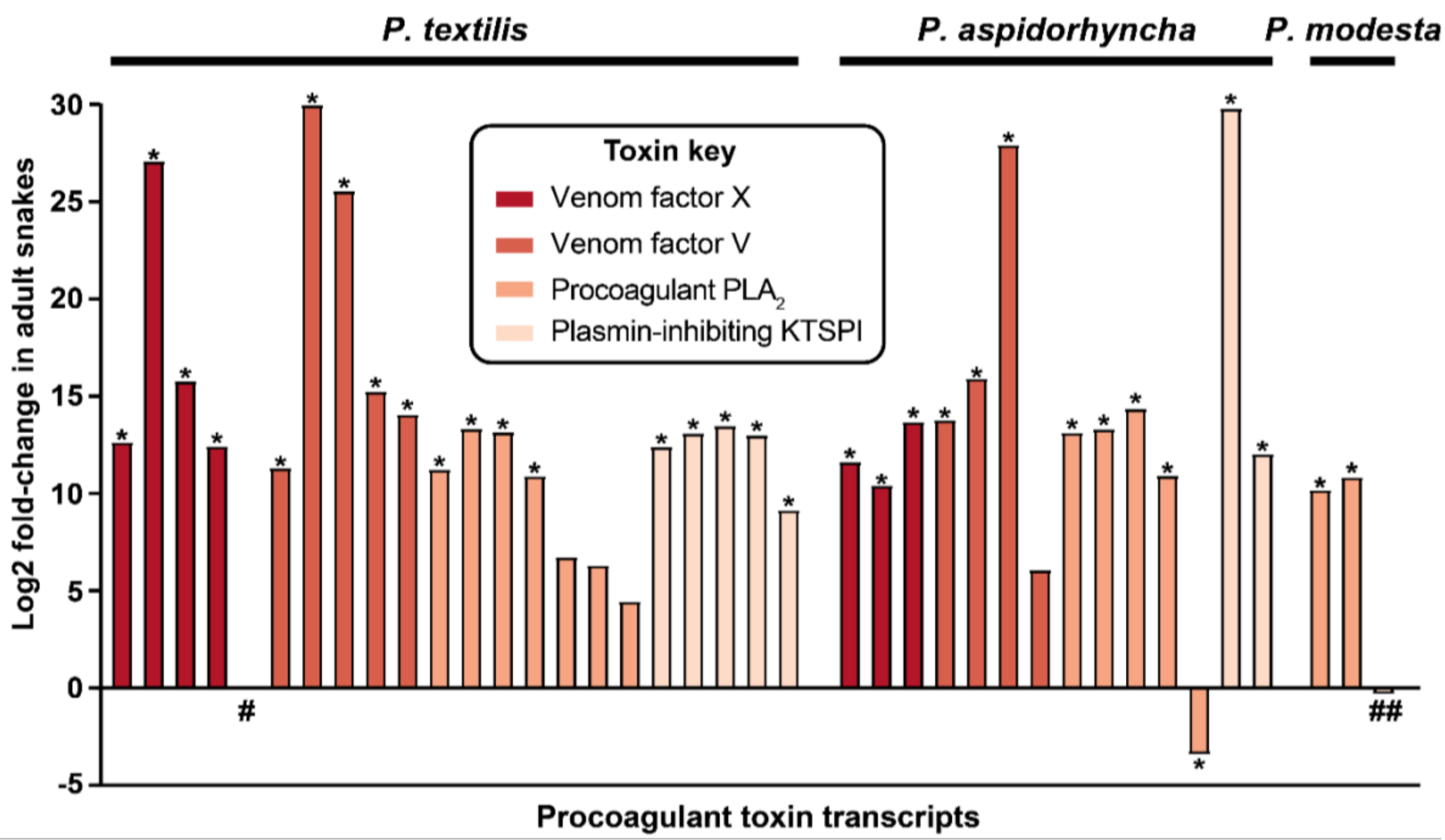

Fig. S15. Log2FoldChange of procoagulant toxin transcripts in adult venom gland tissue of Australian brown snakes (*Pseudonaja* spp.). DESeq2 differential gene expression analyses revealed that nearly all procoagulant toxin transcripts are upregulated in adult venom glands compared to hatchlings. Only one procoagulant PLA_2 transcript is significantly downregulated in *P. aspidorhyncha*. The asterisks (*) indicates statistically significant Log2FoldChange (adjusted $p < 0.05$). The single hashtag (#) indicates the presence of a venom factor Xa transcript that did not make a log2 fold change. The double hashtag (##) indicates the presence of a plasmin-inhibiting KTSPI transcript that did not make a log2 fold change. Abbreviation: KTSPI, Kunitz-type serine protease inhibitor; PLA_2, phospholipase A_2; TPM, transcripts per million.

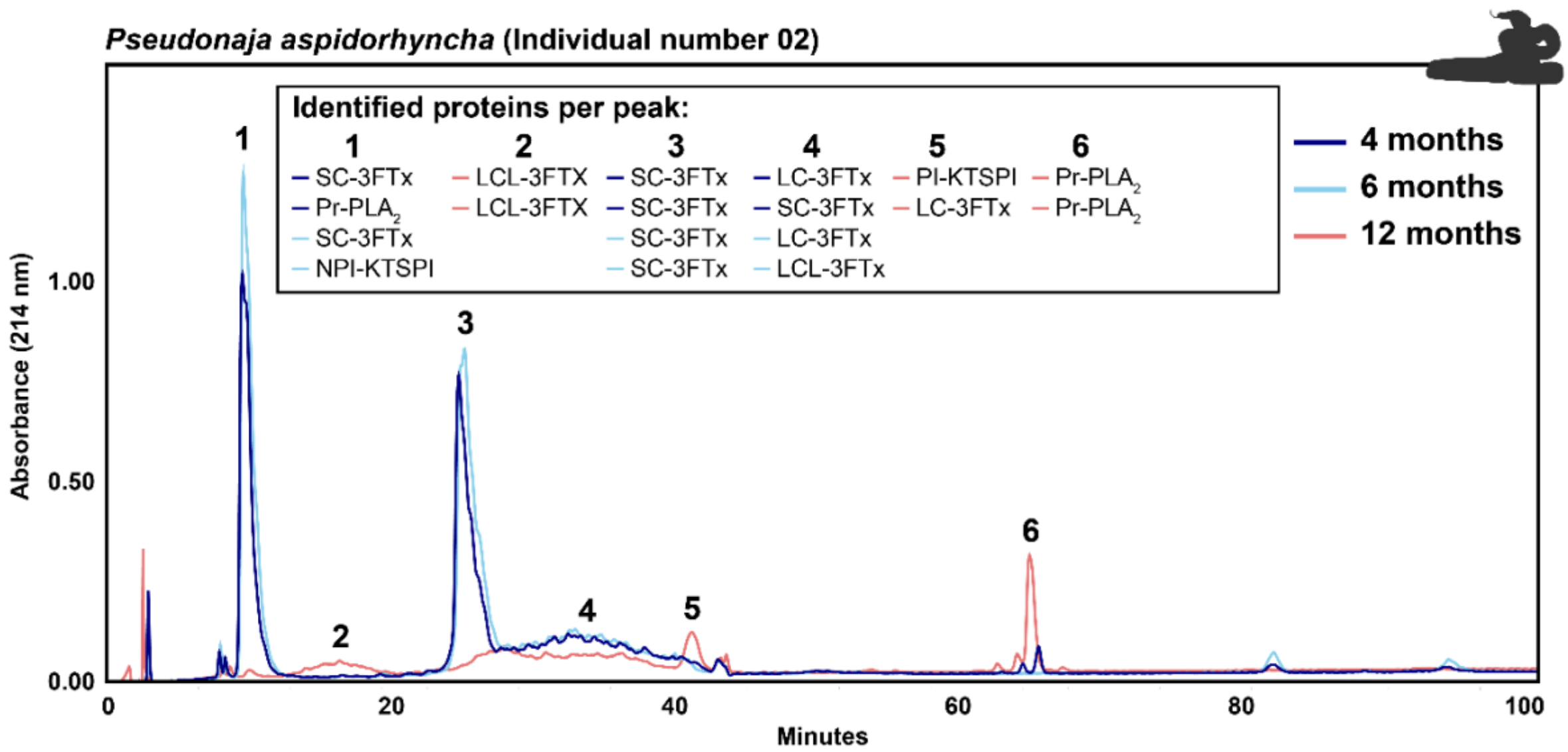

Fig. S16. Chromatographic venom profiles of Strap-snouted brown snake (*Pseudonaja aspidorhyncha*) individual number 02. Reverse-phase chromatographic separation of *P. aspidorhyncha* venom, followed by tandem mass spectrometry (liquid chromatography-mass spectrometry), revealed that, after the dietary shift, two out of four procoagulant toxin families were detectable. Specifically, only plasmin-inhibiting Kunitz-type serine protease inhibitors and procoagulant phospholipase A_2 were identified. The peak numbers corresponded to the two most abundant proteins observed in each respective peak. Notably, venom factor Va and venom factor Xa were absent from the detected peaks. Abbreviations: LC-3FTx, long-chain three-finger toxin; LCL-3FTx, long-chain-like three-finger toxin; NPI-KTSPI, non-plasmin-inhibiting Kunitz-type serine protease inhibitor; PI-KTSPI, plasmin-inhibiting Kunitz-type serine protease inhibitor; Pr-PLA2, procoagulant phospholipase A_2; SC-3FTx, short-chain three-finger toxin.

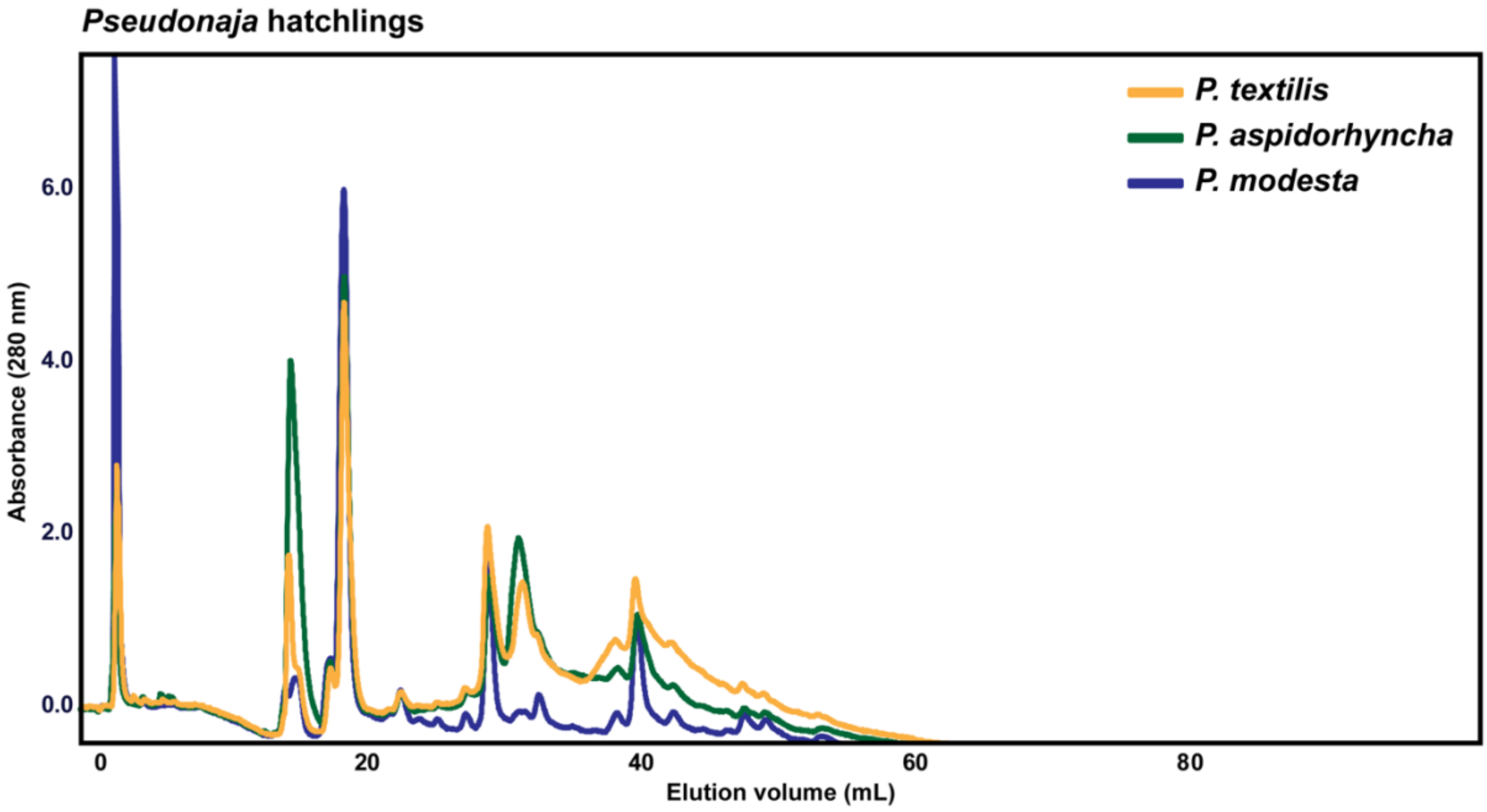

Fig. S17. Comparative chromatographic venom profiles of hatchling *Pseudonaja* species. Reverse-phase chromatographic separation of pooled hatchling venoms (n = 2 per species).

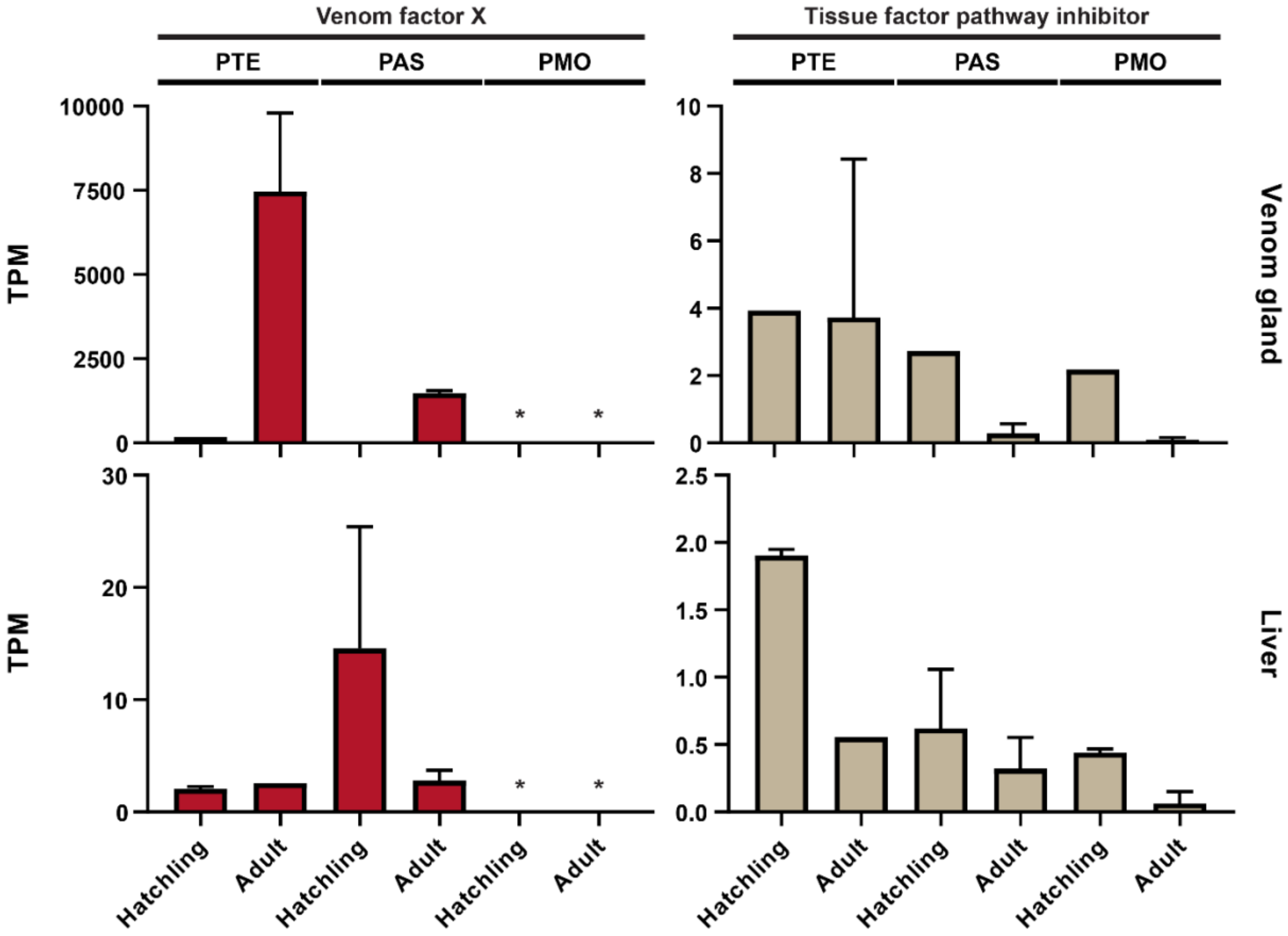

Fig. S18. Comparative expression levels of tissue factor pathway inhibitors in venom glands and livers. Total TPM value of tissue factor pathway inhibitors was calculated from each transcriptome, and the average was taken from each study group (i.e. hatchling or adult per species) and displayed together with the standard deviation. The expression levels of factor X only include the venom-specific isoforms. Each bar chart presents data from two biological replicates, except for the *Pseudonaja textilis* liver tissue, where only one measurement was taken. Asterisk indicates non-expression of genes (*). GenBank accession numbers: XM_026696930.1 and XM_026696931.1. Abbreviations: PAS, *Pseudonaja aspidorhyncha*; PTE, *Pseudonaja textilis*; PMO, *Pseudonaja modesta*; TPM, transcript per million.

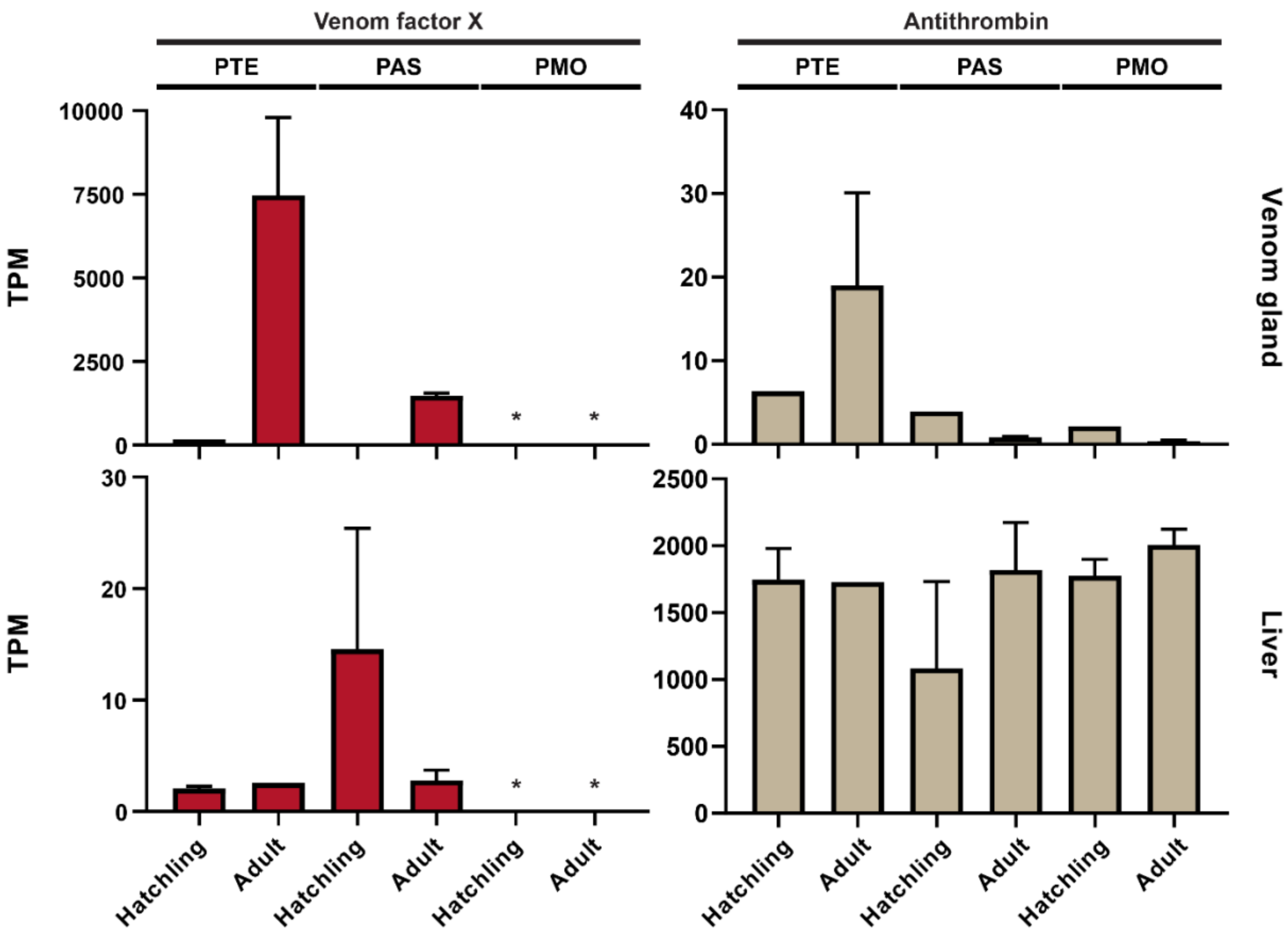

Fig. S19. Comparative expression levels of antithrombin in venom glands and livers. Total TPM value of antithrombin was calculated from each transcriptome, and the average was taken from each study group (i.e. hatchling or adult per species) and displayed together with the standard deviation. Each bar chart presents data from two biological replicates, except for the *Pseudonaja textilis* liver tissue, where only one measurement was taken. Asterisk indicates non-expression of genes (*). GenBank accession numbers: XM_026703355.1 and XM_026703356.1. Abbreviations: PAS, *Pseudonaja aspidorhyncha*; PTE, *Pseudonaja textilis*; PMO, *Pseudonaja modesta*; TPM, transcript per million.

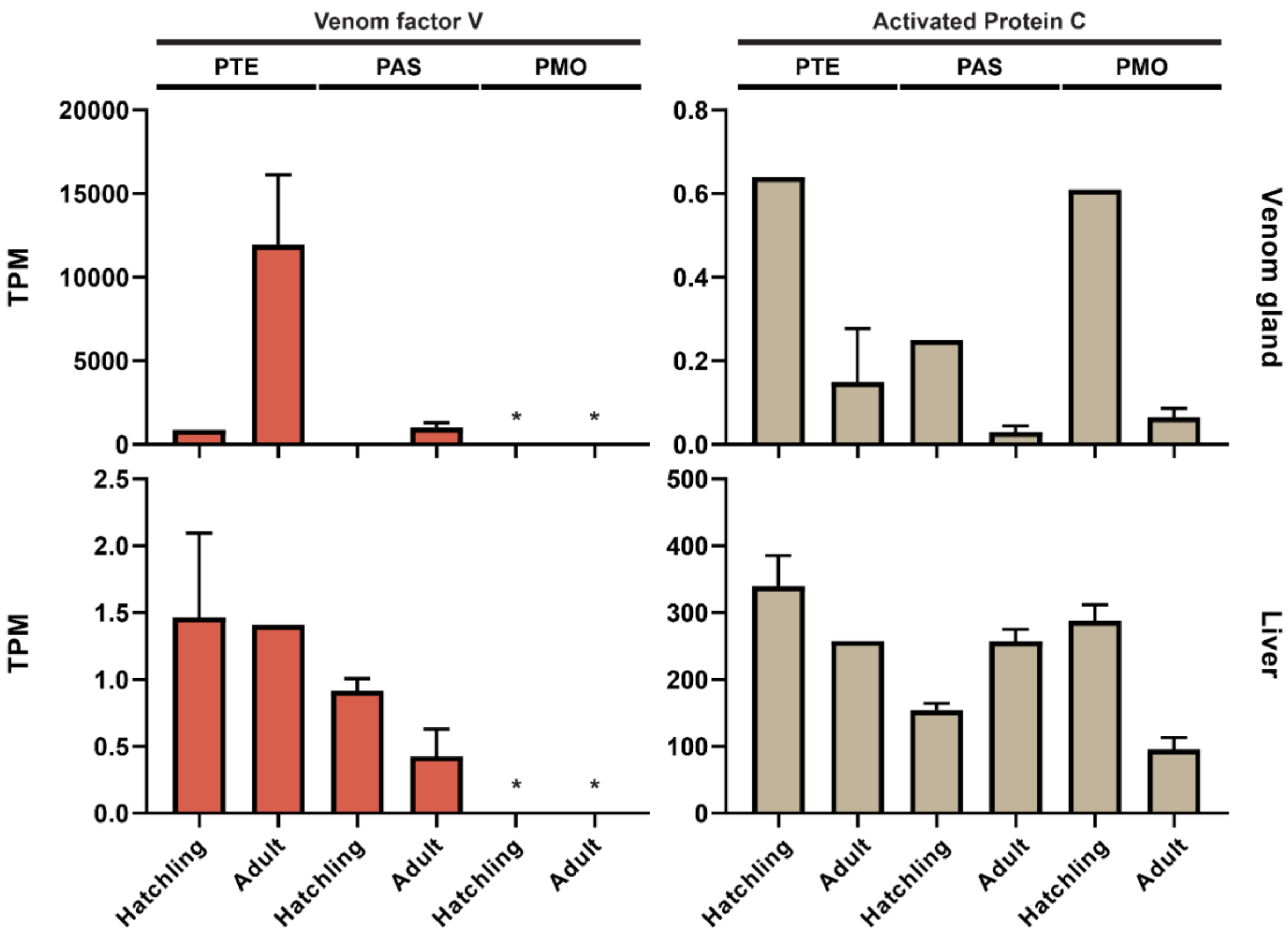

Fig. S20. Comparative expression levels of activated protein C in venom glands and livers. Total TPM value of activated protein C was calculated from each transcriptome, and the average was taken from each study group (i.e. hatchling or adult per species) and displayed together with the standard deviation. The expression levels of factor V only include the venom-specific isoforms. Each bar chart presents data from two biological replicates, except for the Pseudonaja textilis liver tissue, where only one measurement was taken. Asterisk indicates non-expression of genes (*). GenBank accession number: XM_026701308.1. Abbreviations: PAS, *Pseudonaja aspidorhyncha*; PTE, *Pseudonaja textilis*; PMO, *Pseudonaja modesta*; TPM, transcript per million.

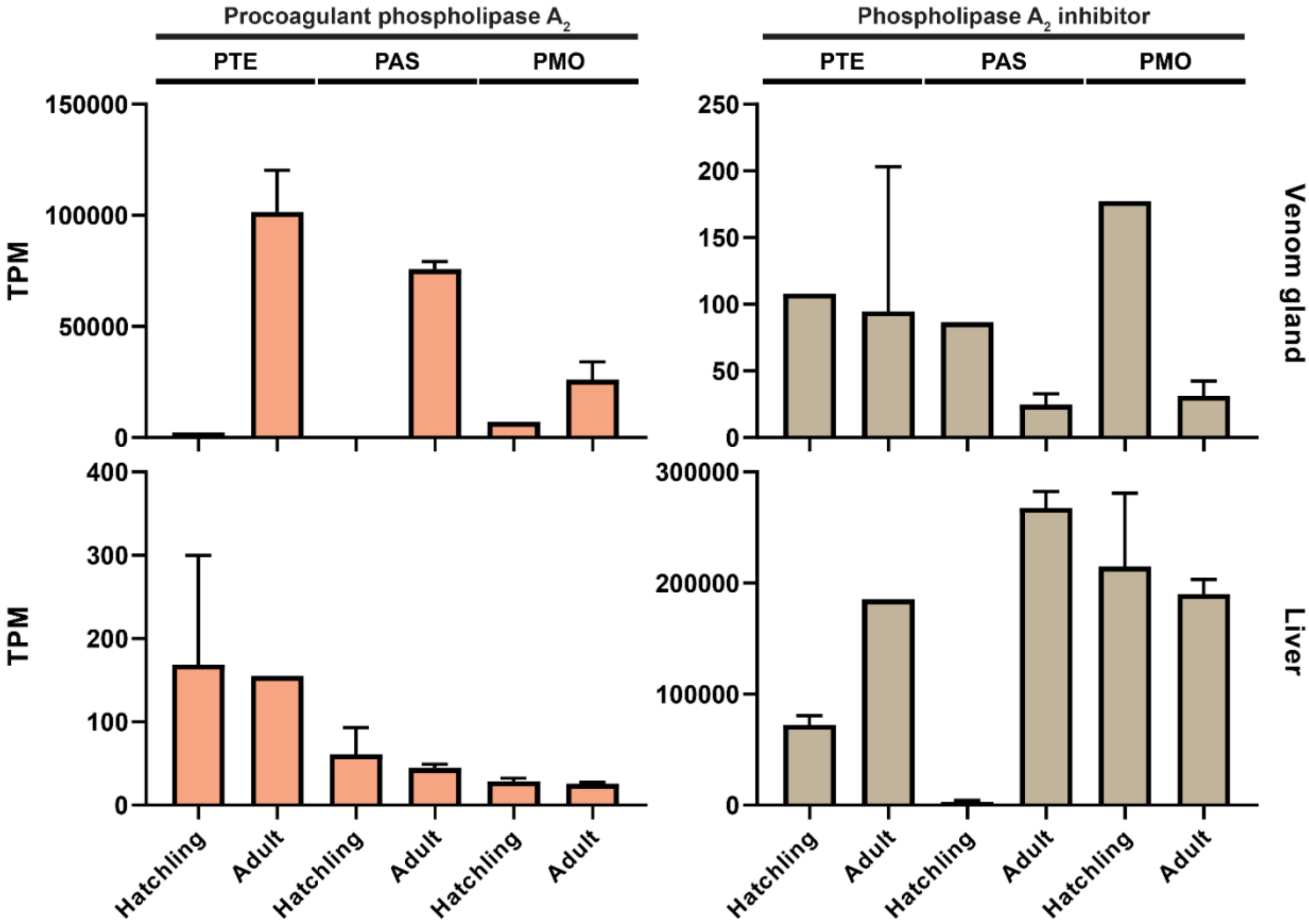

Fig. S21. Comparative expression levels of phospholipase A_2 inhibitors in venom glands and livers. Total TPM value of phospholipase A_2 inhibitors was calculated from each transcriptome, and the average was taken from each study group (i.e. hatchling or adult per species) and displayed together with the standard deviation. From the three structural classes of phospholipase A_2 inhibitors (PLA_2Is), only PLA_2I-β and PLA_2I-γ (both subunit A and B) were identified. PLA_2I-α was absent from both venom gland and liver transcriptomes. GenBank accession numbers: XM_026724407.1, XM_026726430.1 and XM_026724407.1 (PLA_2I-β, respectively) and XM_026725345.1, XM_026725750.1, XM_026725345.1, XM_026725634.1 and XM_026725634.1 (PLA_2I-γ, respectively).

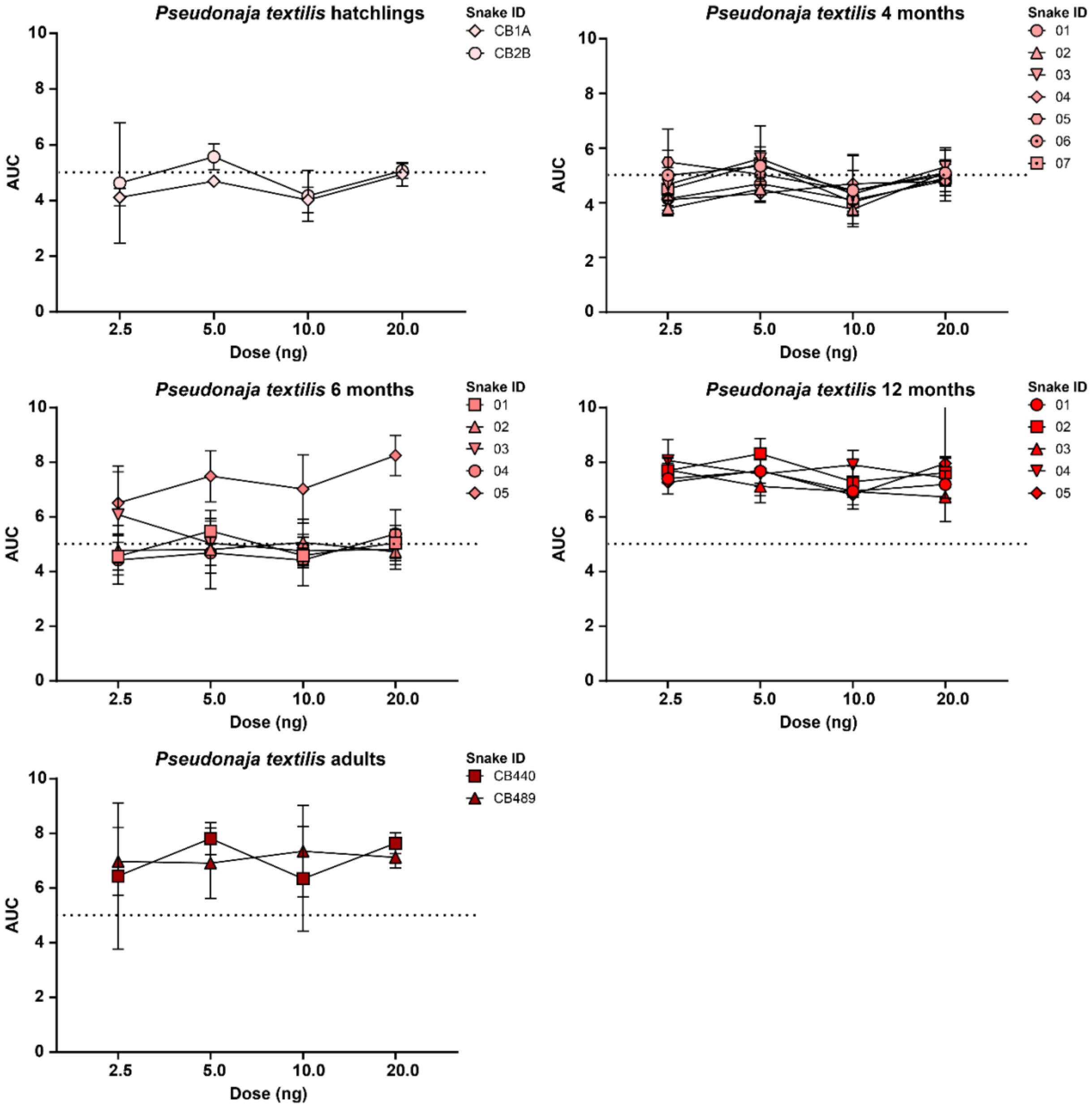

Fig. S22. Coagulation profiles for all Eastern brown snake (*Pseuodnaja textilis*) venoms included in this study. Each data point represents the mean area under the curve (AUC) ± standard deviation. The black dashed line represents the negative control (no venom added) to account for spontaneous clotting (N=5).

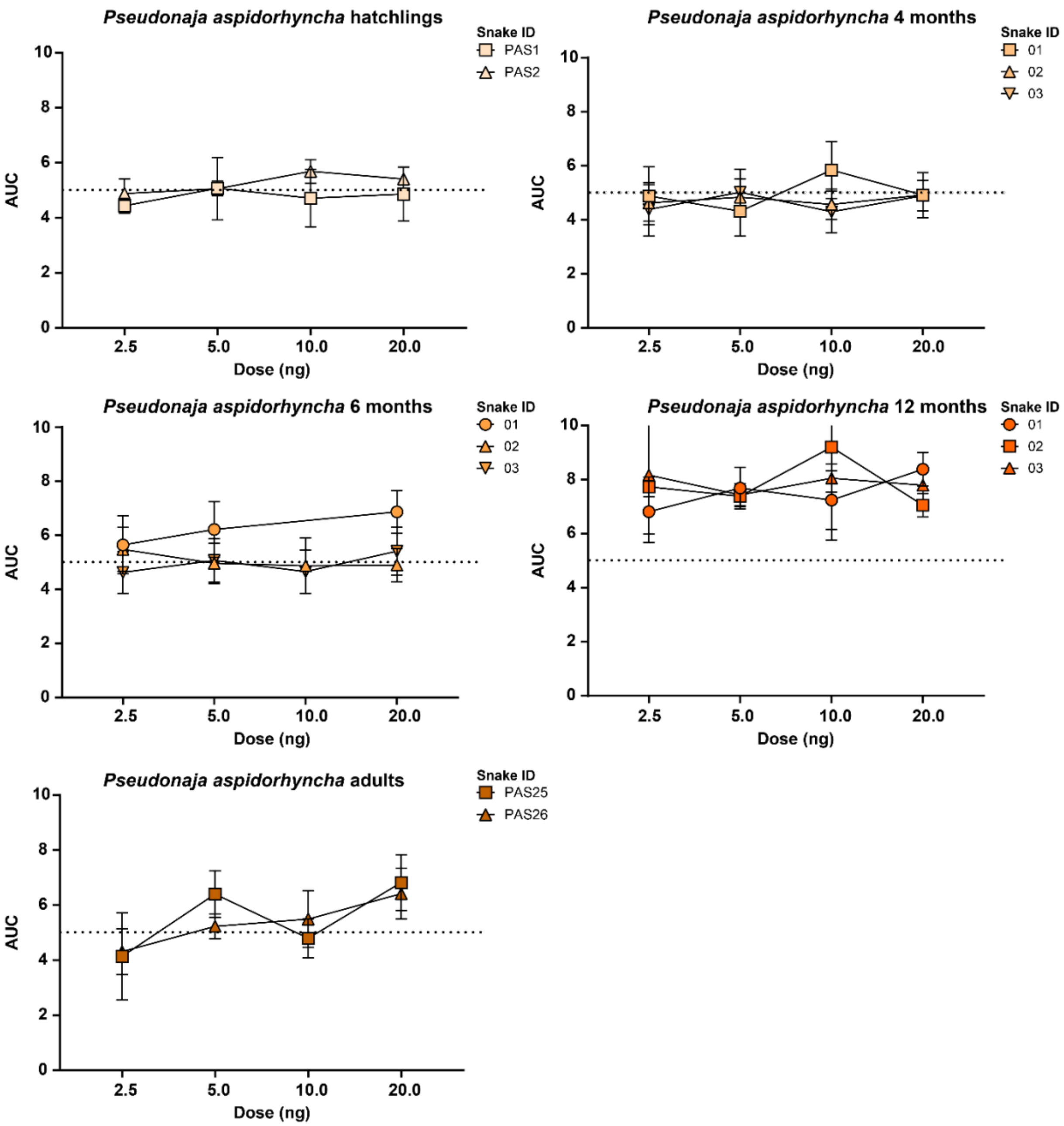

Fig. S23. Coagulation profiles for all strap-snouted brown snake (*Pseudonaja aspidorhyncha*) venoms included in this study. Each data point represents the mean of the area under the curve (AUC) ± standard deviation. The black dashed line represents the negative control (no venom added) to account for spontaneous clotting (N=5).

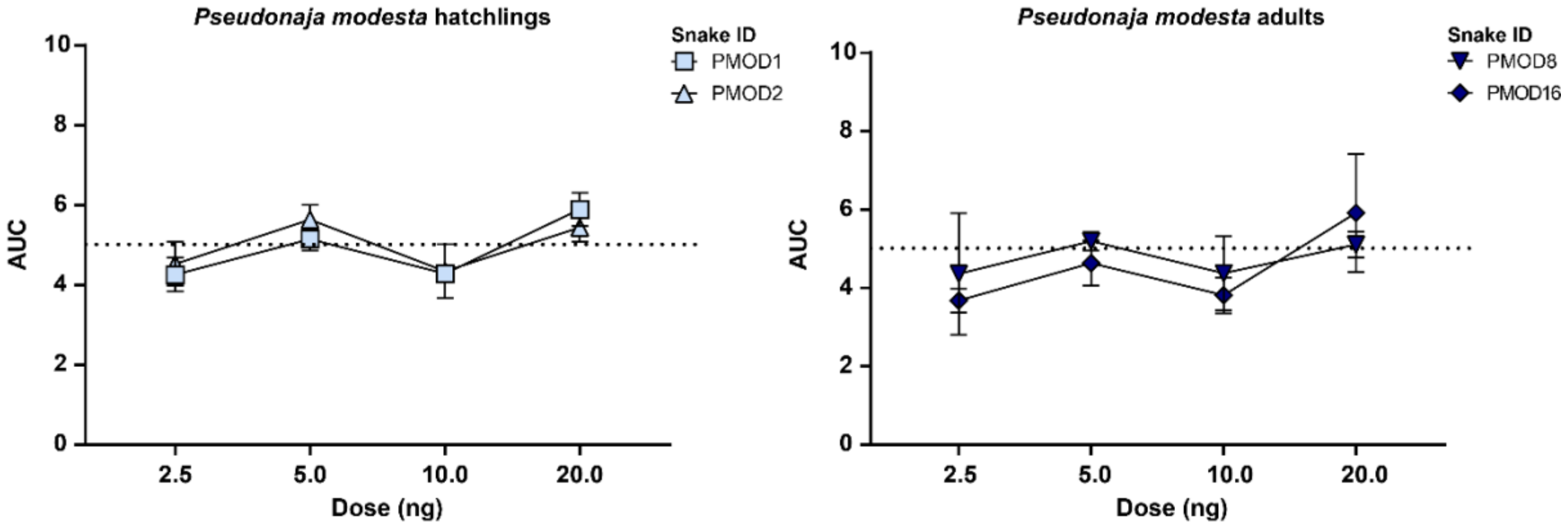

Fig. S24. Coagulation profiles for all ringed brown snake (*Pseudonaja modesta*) venoms included in this study. Each data point represents the mean of the area under the curve (AUC) ± standard deviation. The black dashed line represents the negative control (no venom added) to account for spontaneous clotting (N=5).

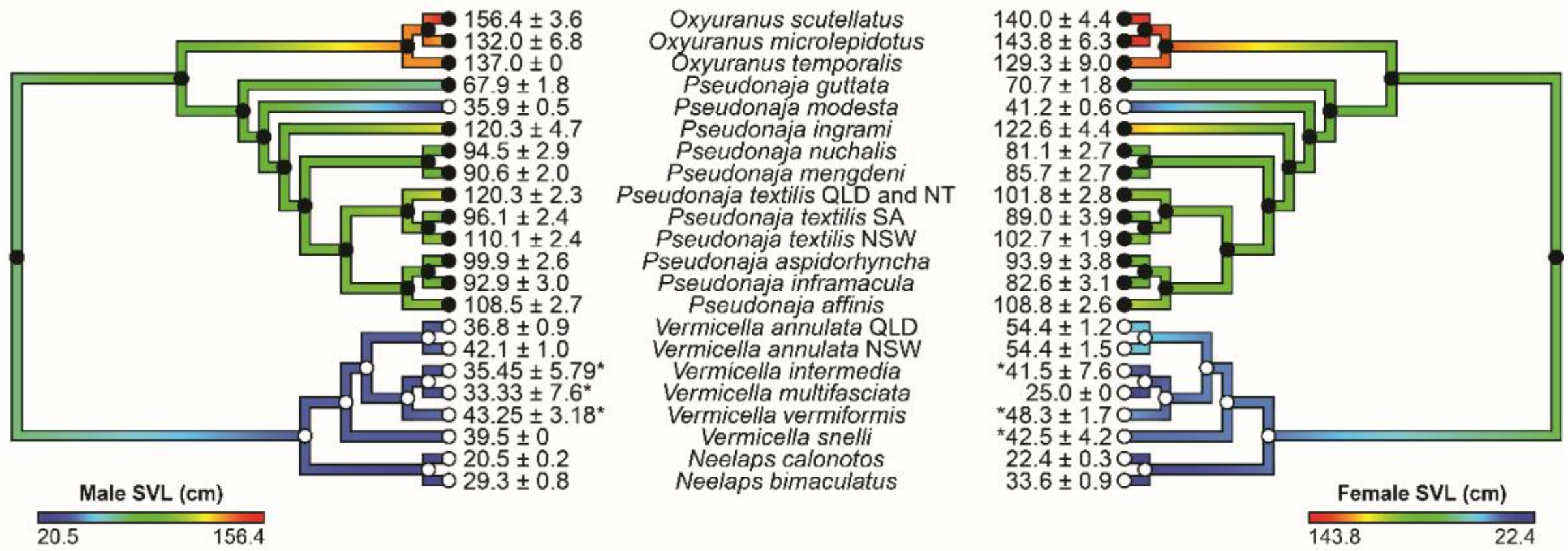

Fig. S25. Ancestral state estimation of adult body size in the monophyletic *Oxyuranus-Pseudonaja* clade reveals a significant reduction in the ringed brown snake (*Pseudonaja modesta*). Ancestral State Estimation (ASE) of the monophyletic *Oxyuranus*-*Pseudonaja clade* revealed that the reduction in adult body size in *P. modesta* corresponds with an evolutionary loss of mammalian prey. ASE was performed using snout–vent length (SVL) as a measure of adult body size, with the analysis rooted with the *Vermicella-Neelaps* sister clade. A color gradient, ranging from blue to red, represents SVL values, where blue indicates smaller SVL (reduced body size) and red indicates larger SVL (increased body size). The presence or absence of mammalian prey is denoted by a black circle (present) or a white circle (absent). An asterisk (*) denotes standard deviation rather than standard error. The analysis was performed separately on SVL data for males (left panel) and females (right panel) to account for sexual dimorphism. The asterisk (*) indicates standard deviation instead of standard error. Abbreviations: NSW, New South Wales; NT, Northern Territory, QLD, Queensland; SA, South Australia; SVL, snout–vent length.

Table S1. Presence and absence of venom gene families in our Australian brown snake species (*Pseudonaja* spp.). The presence of a toxin family, indicated by a black cross (X), is determined based on transcript expression level (i.e. transcriptomics) and protein abundance (i.e. proteomics) in any hatchling or adult brown snake. The transcriptomic threshold of being included was 10 transcripts per million (TPM) in any of the specimens. The proteomic threshold of being included was higher than 1% of the total intensity in any of the specimens. Abbreviations: 3FTx, three-finger toxin; 5'-NT, 5'-nucleotidase; ACHE, acetylcholinesterase; CRISP, cysteine-rich secretory protein; CVF, cobra venom factor; HYLA, hyaluronidase; KTSPI, Kunitz-type serine protease inhibitor; NGF, nerve growth factor; NP, natriuretic peptide; PDE, phosphodiesterase; PLA_2, phospholipase A_2; PLB, phospholipase B; CTL, C-type lectins; SVMP, snake venom metalloproteinase; VEGFA, Vascular Endothelial Growth Factor A; vFVa, venom factor Va; vFXa, venom factor Xa.

		Pseudonaja textilis		***Pseudonaja aspidorhyncha***		***Pseudonaja modesta***	
		Transcriptomics	Proteomics	Transcriptomics	Proteomics	Transcriptomics	Proteomics
**1**	**3FTx**	X	X	X	X	X	X
**2**	**5'-NT**					X	
**3**	**ACHE**	X		X		X	
**4**	**CRISP**	X	X	X	X	X	X
**5**	**CVF**	X		X			
**6**	**HYLA**	X		X		X	
**7**	**KTSPI**	X	X	X	X	X	X
**8**	**NGF**	X		X		X	
**9**	**NP**	X		X		X	
**10**	**PDE**	X		X		X	
**11**	**PLA_2**	X	X	X	X	X	

**12**	**PLB**	X		X		X	
**13**	**CTL**	X	X	X	X	X	X
**14**	**SVMP**	X		X		X	
**15**	**VEGFA**	X		X		X	
**16**	**vFVa**	X	X	X	X		
**17**	**vFXa**	X	X	X	X		
**18**	**WAPRIN**	X		X		X	

Table S2. Overview of differential venom gene expression analysis between hatchling and adults venom glands of Eastern brown snake (*Pseudonaja textilis*) identified by DESeq2 algorithm. Only the venom gene families that include procoagulant toxins are shown. Abbreviations: KTSPI, Kunitz-type serine protease inhibitor; PLA_2, phospholipase A_2; vFXa, venom factor Xa; vFVa, venom factor Va; TPM, transcripts per million.

	Toxin-ID	Venom transcript	Hatchlings TPM	Adult 1 TPM	Adult 2 TPM	log2FoldChange in adults	Adjusted p-value	Toxin activity
1	PTE.KTSPI.ID09	KTSPI	1032.77	83295.19	31470.01	12.39	6.51641E-40	Coagulopathy
2	PTE.VFV.ID05	vFVa	275.61	3647.13	2277.29	11.30	4.25374E-33	Coagulopathy
3	PTE.PLA2.ID06	PLA2	591.74	16230.42	8337.99	11.23	4.41857E-25	Coagulopathy
4	PTE.KTSPI.ID07	KTSPI	1414.02	48966.53	82891.25	13.08	8.14508E-20	Coagulopathy
5	PTE.PLA2.ID05	PLA2	522.43	19926.95	37287.86	13.32	1.02455E-19	Coagulopathy
6	PTE.KTSPI.ID16	KTSPI	68.44	5684.9	6033.26	13.70	6.17938E-19	Unknown
7	PTE.PLA2.ID08	PLA2	1093.78	29289.38	69169.63	13.15	3.31167E-18	Coagulopathy
8	PTE.KTSPI.ID01	KTSPI	0	5117.48	911.05	14.93	1.59921E-17	Unknown
9	PTE.VFX.ID02	vFXa	91.14	2557.06	5774.23	12.63	1.04791E-16	Coagulopathy
10	PTE.KTSPI.ID11	KTSPI	0	4082.31	602.97	13.47	7.50297E-15	Coagulopathy
11	PTE.PLA2.ID12	PLA2	0	3623.55	0.08	26.39	1.38943E-14	Neurotoxicity
12	PTE.KTSPI.ID12	KTSPI	0	6936.19	6783.1	16.61	6.76919E-14	Unknown
13	PTE.VFV.ID04	vFVa	3.83	3648.92	22.33	29.97	1.09379E-11	Coagulopathy
14	PTE.VFX.ID03	vFXa	0	1537.22	0	27.07	8.49885E-11	Coagulopathy
15	PTE.PLA2.ID01	PLA2	0	11143.29	0	26.52	1.45213E-10	Neurotoxicity
16	PTE.PLA2.ID11	PLA2	0	5257.04	0	26.25	1.80978E-10	Neurotoxicity
17	PTE.VFV.ID03	vFVa	0	135.19	0.01	25.54	3.84709E-10	Coagulopathy
18	PTE.PLA2.ID02	PLA2	0	4899.45	0	25.24	6.17376E-10	Neurotoxicity
19	PTE.KTSPI.ID13	KTSPI	0	13014.64	0	24.49	2.10203E-09	Unknown
20	PTE.VFV.ID02	vFVa	0.01	19.41	120.29	15.24	1.02582E-07	Coagulopathy
21	PTE.KTSPI.ID06	KTSPI	2233.64	262.38	123604.24	12.98	1.77478E-07	Coagulopathy
22	PTE.VFX.ID06	vFXa	0	5019.31	1.15	15.77	2.24634E-07	Coagulopathy
23	PTE.KTSPI.ID02	KTSPI	202	27.7	9912.88	12.74	3.50726E-07	Unknown
24	PTE.VFX.ID01	vFXa	74.99	0	54.45	12.42	1.86708E-06	Coagulopathy
25	PTE.PLA2.ID07	PLA2	166.15	22869.13	0.56	10.89	1.51211E-05	Coagulopathy
26	PTE.VFV.ID01	vFVa	156.85	0.1	2072.04	14.06	4.32801E-05	Coagulopathy
27	PTE.KTSPI.ID17	KTSPI	0	27.42	3731.9	12.89	8.05014E-05	Unknown
28	PTE.KTSPI.ID03	KTSPI	0	4361.37	0.21	12.25	0.000359305	Unknown
29	PTE.KTSPI.ID08	KTSPI	377.44	16617.46	2.12	9.13	0.000479738	Coagulopathy
30	PTE.PLA2.ID10	PLA2	0	14081.05	0.25	8.64	0.070450417	Neurotoxicity
31	PTE.PLA2.ID03	PLA2	0	0.63	5.84	6.72	0.12004098	Coagulopathy
32	PTE.PLA2.ID04	PLA2	0	1.34	4.65	6.30	0.131285302	Coagulopathy

33	PTE.PLA2.ID09	PLA2	0	5.53	13.11	4.44	NA	Coagulopathy
34	PTE.VFX.ID05	vFXa	16.57	1.01	0.03	0.10	NA	Coagulopathy

Table S3. Overview of differential venom gene expression analysis between hatchling and adults venom glands of strap-snouted brown snake (*Pseudonaja aspidorhyncha*) identified by DESeq2 algorithm. Only the venom gene families that include procoagulant toxins are shown. Abbreviations: KTSPI, Kunitz-type serine protease inhibitor; PLA_2, phospholipase A_2; vFXa, venom factor Xa; vFVa, venom factor Va; TPM, transcripts per million.

	Toxin-ID	Venom gene	Hatchlings TPM	Adult 1 TPM	Adult 2 TPM	log2FoldChange in adults	Adjusted p-value	Toxin activity
1	PAS.PLA2.ID5	PLA2	77.1	32667.58	35018.28	12.20	7,15086E-57	Coagulopathy
2	PAS.PLA2.ID4	PLA2	6.16	5736.19	5258.40	13.30	3,95109E-49	Coagulopathy
3	PAS.VFX.ID2	vFXa	2.05	539.51	355.78	11.61	7,22212E-30	Coagulopathy
4	PAS.VFV.ID1	vFVa	0.35	1185.2	253.97	13.76	1,25975E-22	Coagulopathy
5	PAS.KTSPI.ID6	KTSPI	7347.22	0	0.00	-12.89	1,03311E-13	Unknown
6	PAS.VFV.ID2	vFVa	0.01	26.14	5.51	15.90	4,51732E-10	Coagulopathy
7	PAS.VFX.ID3	vFXa	3.25	22.51	1063.08	10.41	4,9471E-08	Coagulopathy
8	PAS.KTSPI.ID3	KTSPI	0	10124.28	0.00	29.79	2,26961E-08	Coagulopathy
9	PAS.VFV.ID4	vFVa	0.01	0	35.52	27.90	8,32894E-08	Coagulopathy
10	PAS.VFX.ID1	vFXa	0.45	971.28	0.11	13.68	6,5524E-06	Coagulopathy
11	PAS.PLA2.ID1	PLA2	1.65	4010.58	0.21	14.35	5,84465E-06	Coagulopathy
12	PAS.PLA2.ID3	PLA2	16.17	1.3	10519.67	10.91	6,84579E-05	Coagulopathy
13	PAS.KTSPI.ID1	KTSPI	130.8	15574.02	217.03	8.69	0,000118794	Unknown
14	PAS.KTSPI.ID4	KTSPI	80.26	44580.93	2.61	12.02	0,000123778	Coagulopathy
15	PAS.KTSPI.ID9	KTSPI	6.04	13915.87	2.41	14.59	0,000238469	Unknown
16	PAS.PLA2.ID2	PLA2	33.19	0.24	0.45	-3.41	0,002145386	Coagulopathy
17	PAS.KTSPI.ID5	KTSPI	2439.83	4.49	0.00	-6.24	0,007912785	Unknown
18	PAS.KTSPI.ID2	KTSPI	1463.17	5735.28	131.54	5.08	0,030616594	Unknown
19	PAS.VFV.ID3	vFVa	26	0.51	504.58	6.06	0,055552905	Coagulopathy
20	PAS.KTSPI.ID7	KTSPI	1320.14	5835.96	317.22	4.92	0,146349415	Unknown
21	PAS.KTSPI.ID8	KTSPI	4307.6	1095.37	242.17	1.02	0,642680766	Unknown
22	PAS.KTSPI.ID11	KTSPI	20.84	0	3.26	1.11	0,643960331	Unknown
23	PAS.KTSPI.ID10	KTSPI	36.62	1554.32	1886.93	-1.02	#N/A	Unknown

Table S4. Overview of differential venom gene expression analysis between hatchling and adults venom glands of ringed brown snake (*Pseudonaja modesta*) identified by DESeq2 algorithm. Only the venom gene families that include procoagulant toxins are shown. Abbreviations: KTSPI, Kunitz-type serine protease inhibitor; PLA_2, phospholipase A_2; TPM, transcripts per million.

	Toxin-ID	Venom gene	Hatchlings TPM	Adult 1 TPM	Adult 2 TPM	log2FoldChange in adults	Adjusted p-value	Toxin activity
1	PMO.PLA2.ID01	PLA2	1156.69	4785.43	9655.76	10.18	4,78783E-92	Coagulopathy
2	PMO.KTSPI.ID03	KTSPI	113.98	5883.73	21596.84	14.49	1,26743E-64	Unknown
3	PMO.KTSPI.ID02	KTSPI	42428.23	0	0	-11.46	2,15257E-19	Unknown
4	PMO.KTSPI.ID06	KTSPI	0	78.82	260.08	12.80	2,06403E-12	Unknown
5	PMO.KTSPI.ID04	KTSPI	24762.36	3294.17	16221.63	6.20	7,50352E-12	Unknown
6	PMO.PLA2.ID02	PLA2	204.77	4775.93	9.46	10.83	6,84318E-09	Coagulopathy
7	PMO.KTSPI.ID05	KTSPI	0	10.47	83.75	10.73	6,27557E-08	Unknown
8	PMO.KTSPI.ID01	KTSPI	151.86	7639.96	2.14	11.51	0,000100521	Unknown
9	PMO.KTSPI.ID07	KTSPI	822.91	3.57	4.18	-0.31	0,75824653	Coagulopathy
10	PMO.KTSPI.ID08	KTSPI	476.42	0	0.93	-2.47	#N/A	Unknown

Table S5. Overview of venom and tissue samples included in this study. Abbreviations: QLD, Queensland; SA, South Australia; SVL, snout–vent length.

Species	Snake ID	Locality	Developmental life stage	Size (SVL; cm)	Included in following venom analysis:			
					Proteomics	Transcriptomics	Clotting assay	Prothrombin assay
P. aspidorhyncha	PAS1	Cunnamulla, QLD	Hatchling	NA	Yes	Yes	Yes	Yes
P. aspidorhyncha	PAS2	Cunnamulla, QLD	Hatchling	NA	Yes	Yes	Yes	Yes
P. modesta	PMOD1	Mixed heritage	Hatchling	NA	Yes	Yes	Yes	No
P. modesta	PMOD2	Mixed heritage	Hatchling	NA	Yes	Yes	Yes	No
P. textilis	CB1A	Myola, QLD	Hatchling	NA	Yes	Yes	Yes	Yes
P. textilis	CB2B	Myola, QLD	Hatchling	NA	Yes	Yes	Yes	Yes
P. aspidorhyncha	01	Cunnamulla, QLD	4 months	NA	Yes	No	Yes	Yes
P. aspidorhyncha	02	Cunnamulla, QLD	4 months	NA	Yes	No	Yes	Yes
P. aspidorhyncha	03	Cunnamulla, QLD	4 months	NA	Yes	No	Yes	Yes
P. textilis	01	Myola, QLD	4 months	NA	Yes	No	Yes	Yes
P. textilis	02	Myola, QLD	4 months	NA	Yes	No	Yes	Yes
P. textilis	03	Myola, QLD	4 months	NA	Yes	No	Yes	Yes
P. textilis	04	Myola, QLD	4 months	NA	Yes	No	Yes	Yes
P. textilis	05	Myola, QLD	4 months	NA	Yes	No	Yes	Yes
P. textilis	06	Myola, QLD	4 months	NA	Yes	No	Yes	No
P. textilis	07	Myola, QLD	4 months	NA	Yes	No	Yes	No
P. aspidorhyncha	01	Cunnamulla, QLD	6 months	41.0	Yes	No	Yes	Yes
P. aspidorhyncha	02	Cunnamulla, QLD	6 months	34.2	Yes	No	Yes	Yes
P. aspidorhyncha	03	Cunnamulla, QLD	6 months	37.1	Yes	No	Yes	Yes
P. textilis	01	Myola, QLD	6 months	35.5	Yes	No	Yes	Yes
P. textilis	02	Myola, QLD	6 months	37.0	Yes	No	Yes	Yes
P. textilis	03	Myola, QLD	6 months	35.6	Yes	No	Yes	Yes
P. textilis	04	Myola, QLD	6 months	38.4	Yes	No	Yes	Yes
P. textilis	05	Myola, QLD	6 months	39.6	Yes	No	Yes	Yes

P. aspidorhyncha	01	Cunnamulla, QLD	12 months	62.0	Yes	No	Yes	Yes
P. aspidorhyncha	02	Cunnamulla, QLD	12 months	57.5	Yes	No	Yes	Yes
P. aspidorhyncha	03	Cunnamulla, QLD	12 months	55.5	Yes	No	Yes	Yes
P. textilis	01	Myola, QLD	12 months	75.5	Yes	No	Yes	Yes
P. textilis	02	Myola, QLD	12 months	67.0	Yes	No	Yes	Yes
P. textilis	03	Myola, QLD	12 months	71.0	Yes	No	Yes	Yes
P. textilis	04	Myola, QLD	12 months	69.5	Yes	No	Yes	Yes
P. textilis	05	Myola, QLD	12 months	73.0	Yes	No	Yes	Yes
P. aspidorhyncha	PAS25	Middleback Ranges, SA	Adult	NA	Yes	Yes	Yes	Yes
P. aspidorhyncha	PAS26	Middleback Ranges, SA	Adult	NA	Yes	Yes	Yes	Yes
P. modesta	PMOD8	Mixed heritage	Adult	NA	Yes	Yes	Yes	No
P. modesta	PMOD16	Mixed heritage	Adult	NA	Yes	Yes	Yes	No
P. textilis	CB440	Adelaide region, SA	Adult	NA	Yes	Yes	Yes	Yes
P. textilis	CB489	Adelaide region, SA	Adult	NA	Yes	Yes	Yes	Yes

Table S6. Overview of snout–vent length data for ancestral state estimation analysis. The snout–vent length data for *Pseudonaja mengdeni* (previously mentioned as *P*. "*nuchalis*" "Orange with black head“), *Pseudonaja nuchalis* (previously mentioned as *P*. "*nuchalis*" "Darwin morph“) and *Pseudonaja aspidorhyncha* (previously mentioned as *P*. "*nuchalis*" "Southern morph“ South Australia) was based on their previously descripted subspecies within the former *P. nuchalis* clade. Asterisk (*) indicates standard deviation instead of standard error. Abbreviations: NSW, New South Wales; NT, Northern Territory, QLD, Queensland; SA, South Australia; SE, standard error; SVL, snout–vent length.

	**Adult males**			**Adult females**		
**Species**	**Mean SVL (cm)**	**SE (cm)**	**Sample size (N)**	**Mean SVL (cm)**	**SE (cm)**	**Sample size (N)**
Oxyuranus scutellatus	156.4	3.6	55	140.0	4.4	23
Oxyuranus microlepidotus	132.0	6.8	13	143.8	6.3	6
Oxyuranus temporalis	137.0	0	1	129.3	9.0	3
Pseudonaja guttata	67.9	1.8	65	70.7	1.8	52
Pseudonaja modesta	35.9	0.5	127	41.2	0.6	38
Pseudonaja ingrami	120.3	4.7	9	122.6	4.4	6
Pseudonaja mengdeni	90.6	2.0	54	85.7	2.7	27
Pseudonaja nuchalis	94.5	2.9	22	81.1	2.7	19
Pseudonaja textilis NSW	110.1	2.4	90	102.7	1.9	45

Pseudonaja textilis QLD and NT	120.3	2.3	76	101.8	2.8	54
Pseudonaja textilis SA	96.1	2.4	44	89.0	3.9	38
Pseudonaja inframacula	92.9	3.0	34	82.6	3.1	22
Pseudonaja aspidorhyncha	99.9	2.6	46	93.9	3.8	19
Pseudonaja affinis	108.5	2.7	49	108.8	2.6	35
Vermicella annulata NSW	42.1	1.0	38	54.4	1.5	38
Vermicella annulata QLD	36.8	0.9	46	54.4	1.2	42
Vermicella intermedia	35.45	5.79*	13	41.5	7.6*	15
Vermicella multifasciata	33.33	7.6*	3	25.0	0*	1
Vermicella snelli	39.5	0*	1	42.5	4.2*	5
Vermicella vermiformis	43.25	3.18*	2	48.3	1.7*	3
Neelaps bimaculatus	29.3	0.8	25	33.6	0.9	22
Neelaps calonotos	20.5	0.2	30	22.4	0.3	32
